# Supplementary figures and images for: Mapping mutational effects along the evolutionary landscape of HIV envelope
Source: eLife. 2018 Mar 28;7:e34420. doi: 10.7554/eLife.34420 (PMC5910023; doi:10.7554/eLife.34420)

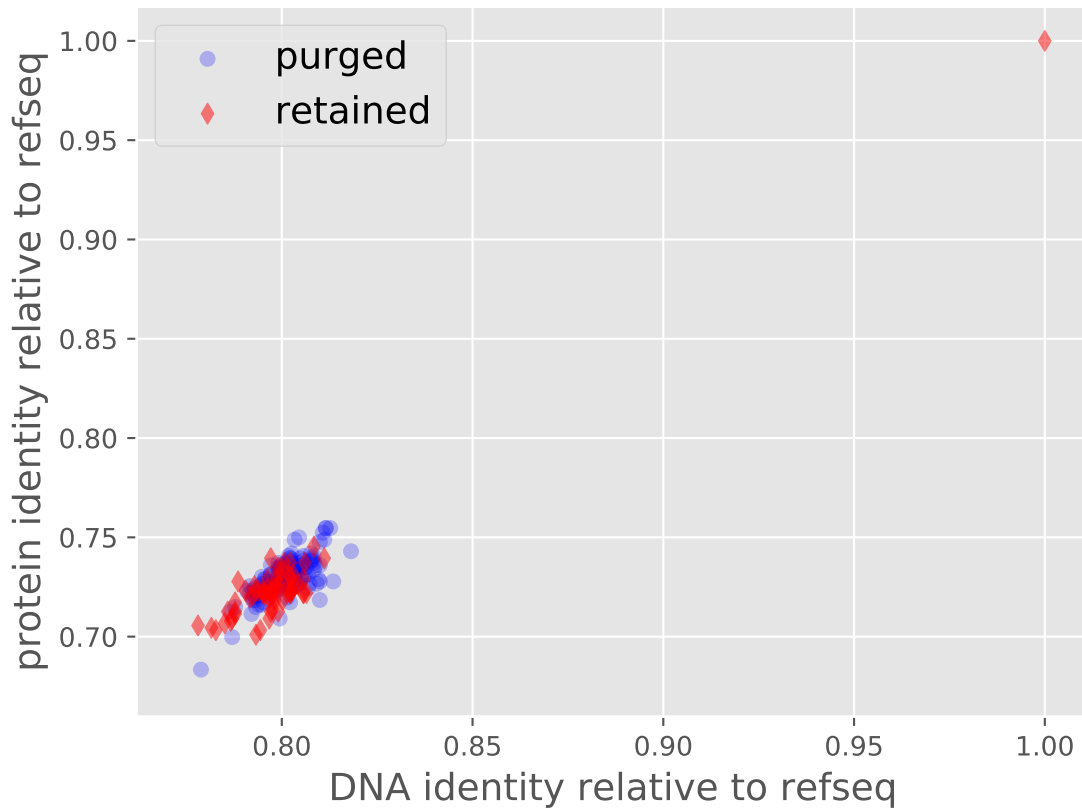

Supplement: Supplementary file 1. — Specifically, this file contains a Jupyter notebook that performs the analysis, all required input data, and all reasonably sized output files. The Jupyter notebook downloads the deep sequencing data, processes it with the dms_tools2 software (Bloom, 2015, https://jbloomlab.github.io/dms_tools2/), and also performs a variety of downstream analyses that generate most of the figures for this paper. [file elife-34420-supp1.zip › analysis_code/results/alignments/cladeA_alignment.pdf]

# clade A protein identity at alignable sites

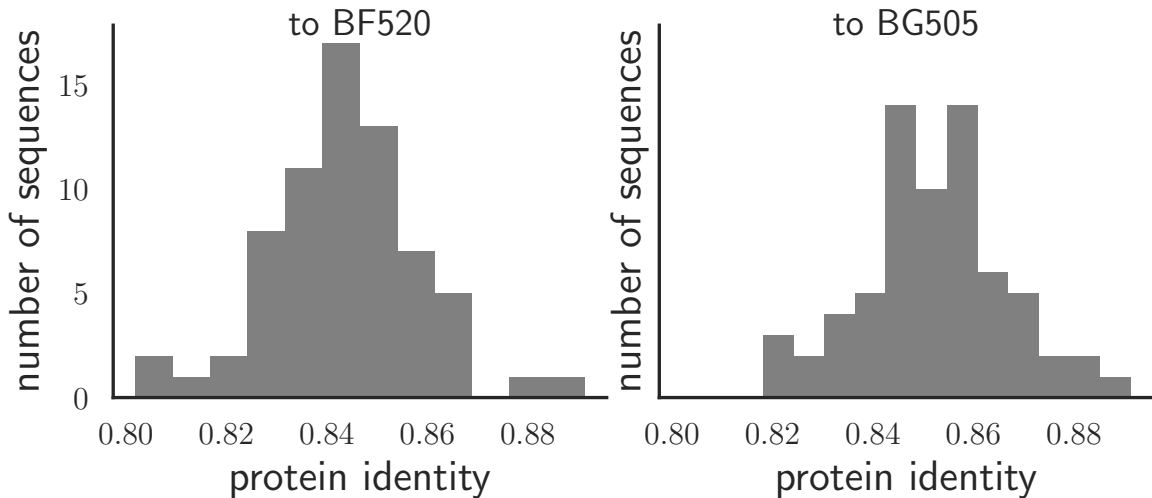

Supplement: Supplementary file 1. — Specifically, this file contains a Jupyter notebook that performs the analysis, all required input data, and all reasonably sized output files. The Jupyter notebook downloads the deep sequencing data, processes it with the dms_tools2 software (Bloom, 2015, https://jbloomlab.github.io/dms_tools2/), and also performs a variety of downstream analyses that generate most of the figures for this paper. [file elife-34420-supp1.zip › analysis_code/results/alignments/masked_alignment_identity.pdf]

number of barcodes

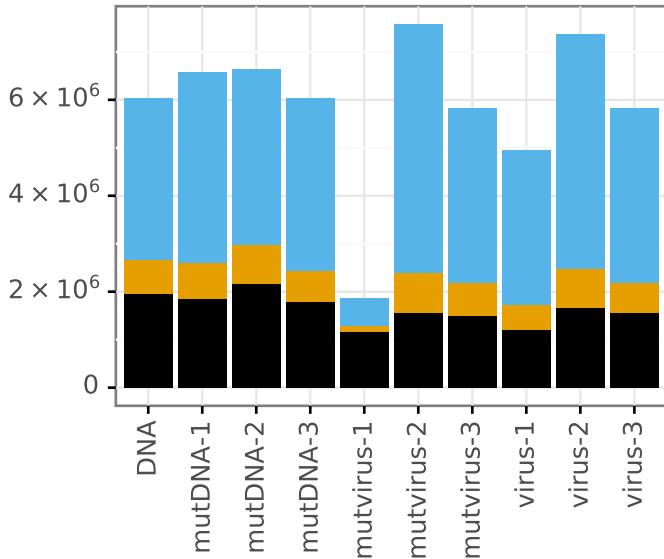

barcode fate

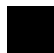

aligned

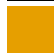

not alignable

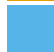

too few reads

Supplement: Supplementary file 1. — Specifically, this file contains a Jupyter notebook that performs the analysis, all required input data, and all reasonably sized output files. The Jupyter notebook downloads the deep sequencing data, processes it with the dms_tools2 software (Bloom, 2015, https://jbloomlab.github.io/dms_tools2/), and also performs a variety of downstream analyses that generate most of the figures for this paper. [file elife-34420-supp1.zip › analysis_code/results/codoncounts/BF520/summary_bcstats.pdf]

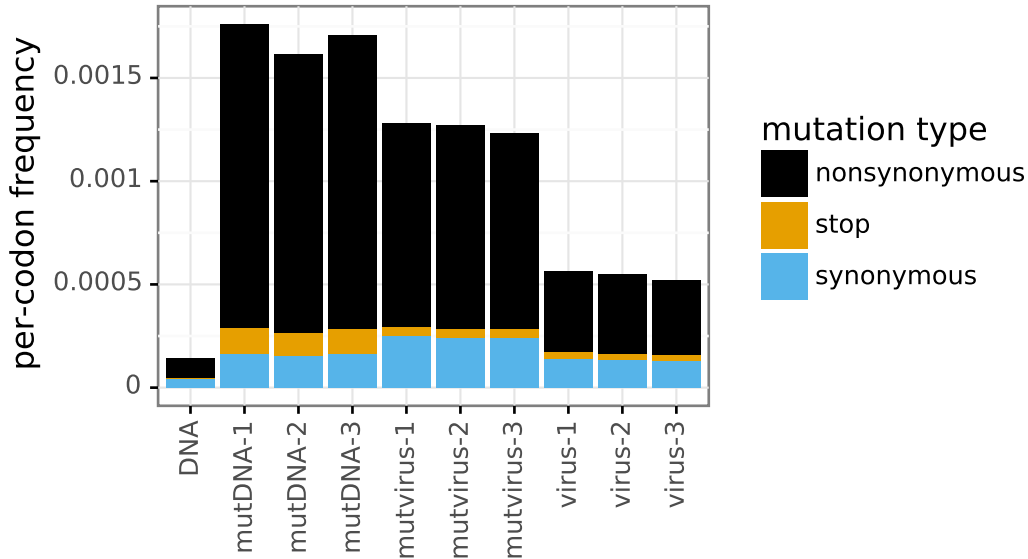

Supplement: Supplementary file 1. — Specifically, this file contains a Jupyter notebook that performs the analysis, all required input data, and all reasonably sized output files. The Jupyter notebook downloads the deep sequencing data, processes it with the dms_tools2 software (Bloom, 2015, https://jbloomlab.github.io/dms_tools2/), and also performs a variety of downstream analyses that generate most of the figures for this paper. [file elife-34420-supp1.zip › analysis_code/results/codoncounts/BF520/summary_codonmuttypes.pdf]

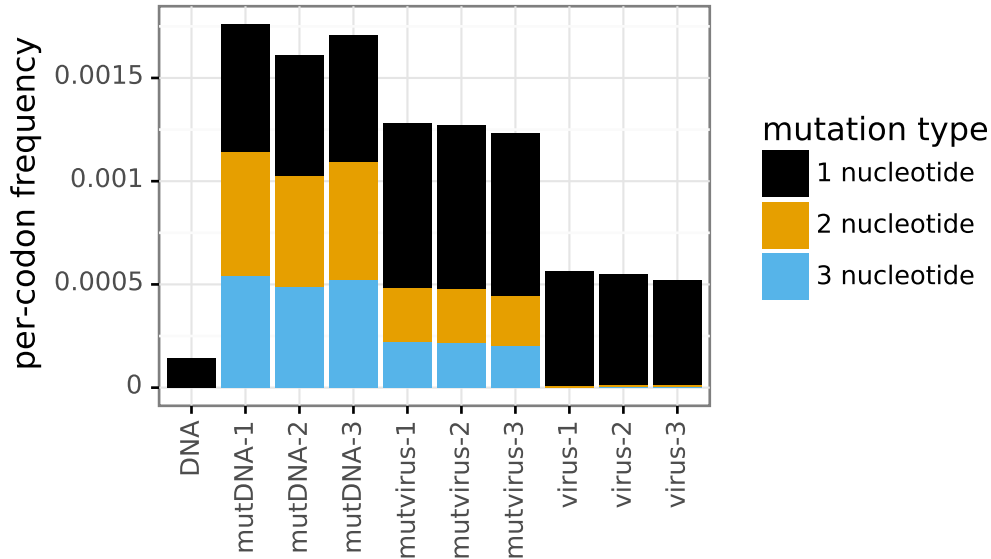

Supplement: Supplementary file 1. — Specifically, this file contains a Jupyter notebook that performs the analysis, all required input data, and all reasonably sized output files. The Jupyter notebook downloads the deep sequencing data, processes it with the dms_tools2 software (Bloom, 2015, https://jbloomlab.github.io/dms_tools2/), and also performs a variety of downstream analyses that generate most of the figures for this paper. [file elife-34420-supp1.zip › analysis_code/results/codoncounts/BF520/summary_codonntchanges.pdf]

— amino acids — codons

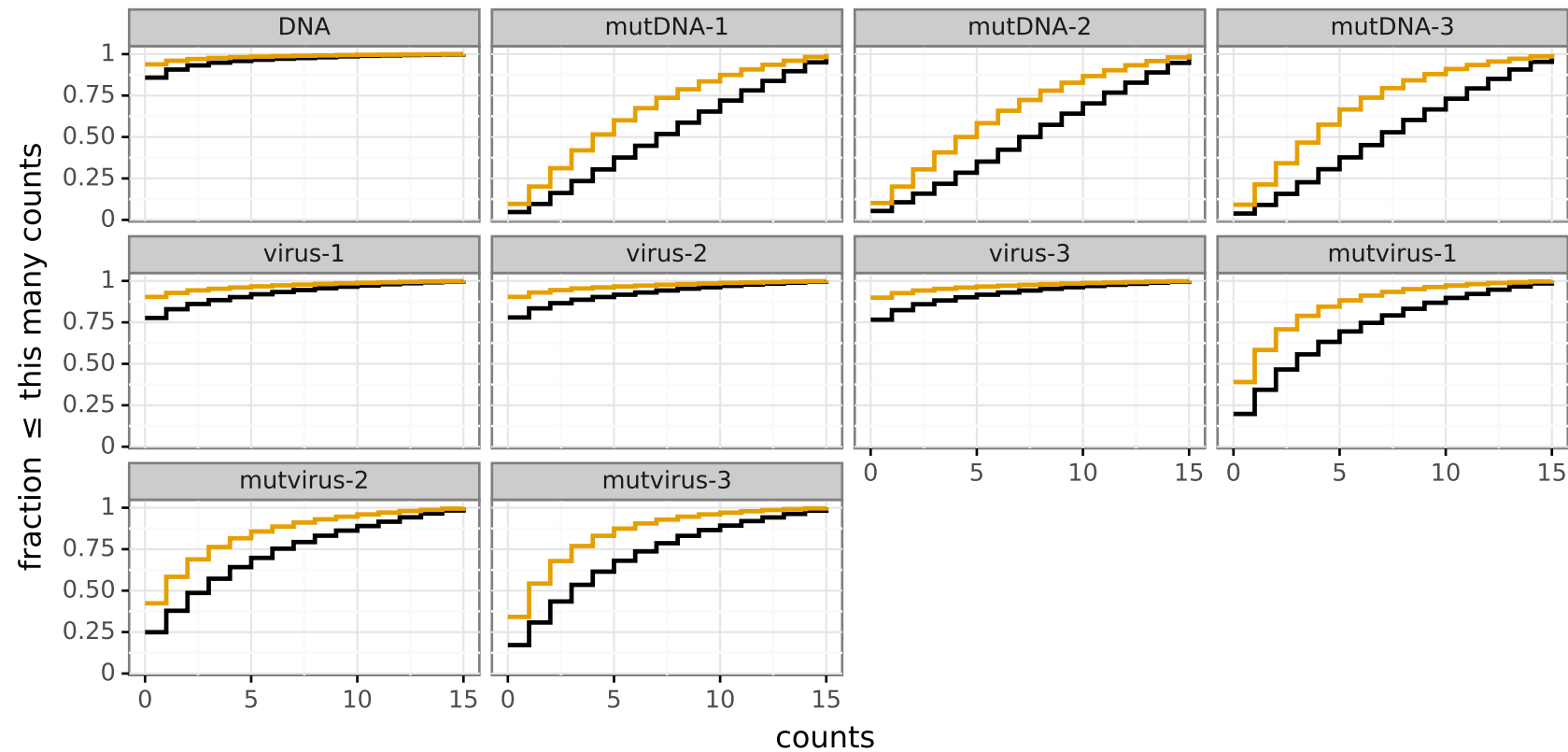

Supplement: Supplementary file 1. — Specifically, this file contains a Jupyter notebook that performs the analysis, all required input data, and all reasonably sized output files. The Jupyter notebook downloads the deep sequencing data, processes it with the dms_tools2 software (Bloom, 2015, https://jbloomlab.github.io/dms_tools2/), and also performs a variety of downstream analyses that generate most of the figures for this paper. [file elife-34420-supp1.zip › analysis_code/results/codoncounts/BF520/summary_cumulmutcounts.pdf]

number of counts

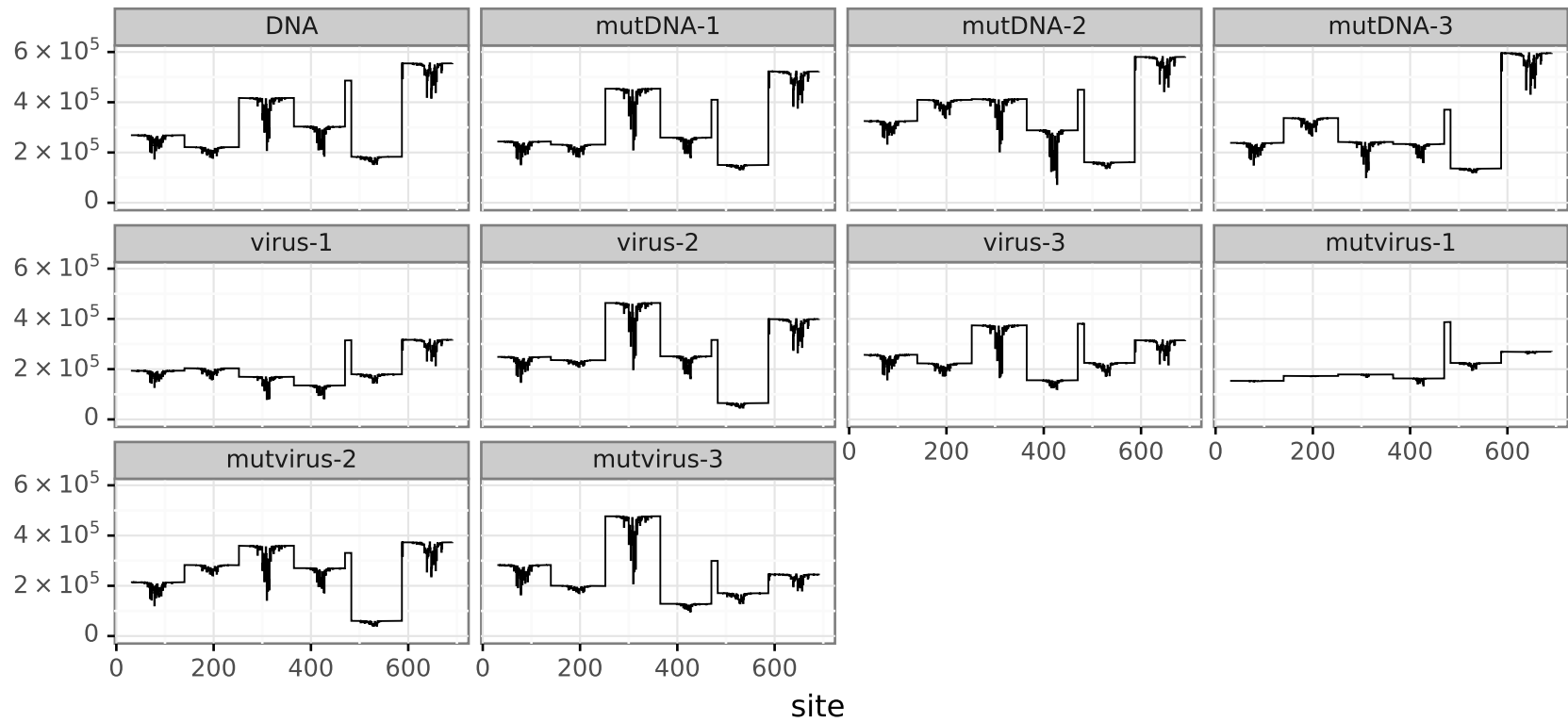

Supplement: Supplementary file 1. — Specifically, this file contains a Jupyter notebook that performs the analysis, all required input data, and all reasonably sized output files. The Jupyter notebook downloads the deep sequencing data, processes it with the dms_tools2 software (Bloom, 2015, https://jbloomlab.github.io/dms_tools2/), and also performs a variety of downstream analyses that generate most of the figures for this paper. [file elife-34420-supp1.zip › analysis_code/results/codoncounts/BF520/summary_depth.pdf]

mutation frequency

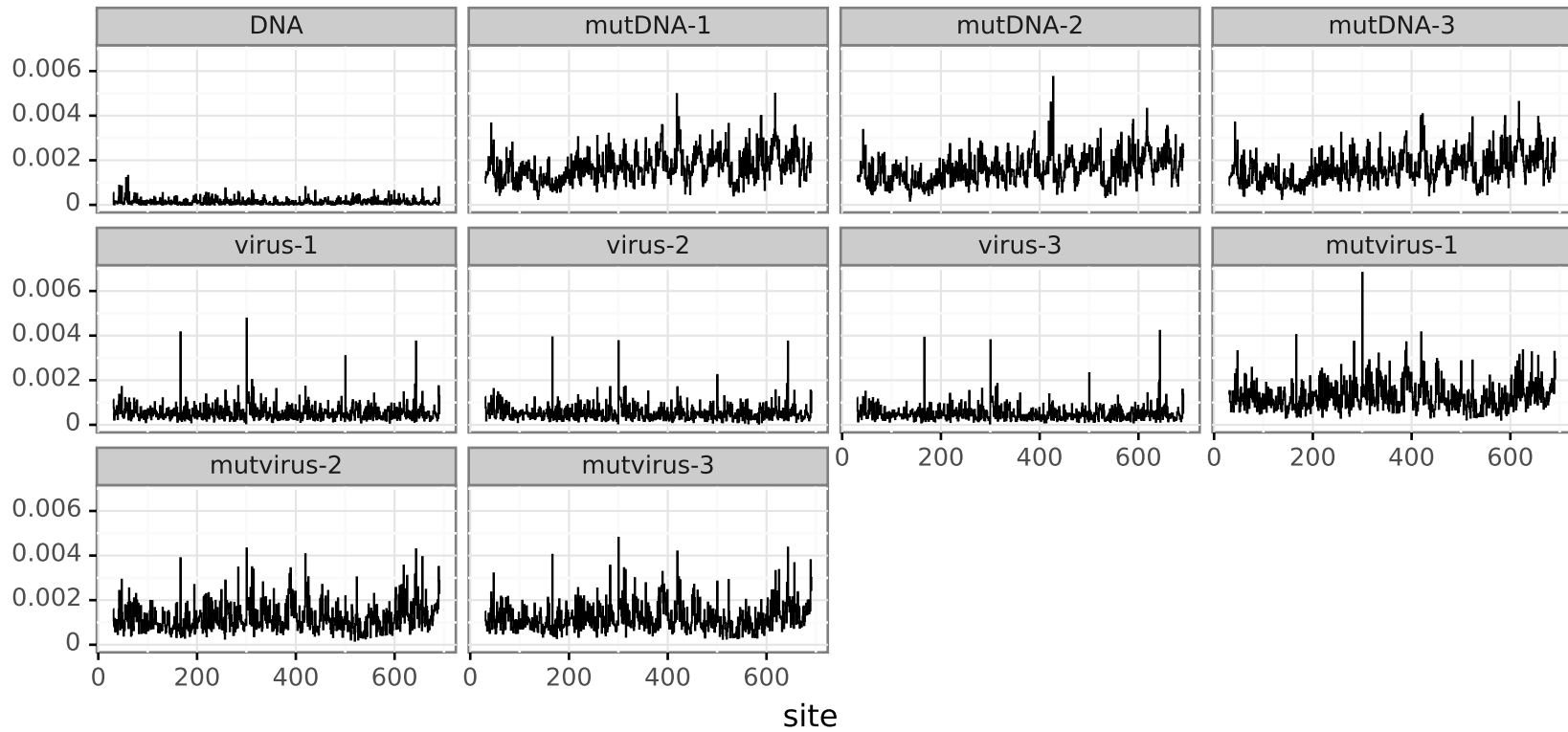

Supplement: Supplementary file 1. — Specifically, this file contains a Jupyter notebook that performs the analysis, all required input data, and all reasonably sized output files. The Jupyter notebook downloads the deep sequencing data, processes it with the dms_tools2 software (Bloom, 2015, https://jbloomlab.github.io/dms_tools2/), and also performs a variety of downstream analyses that generate most of the figures for this paper. [file elife-34420-supp1.zip › analysis_code/results/codoncounts/BF520/summary_mutfreq.pdf]

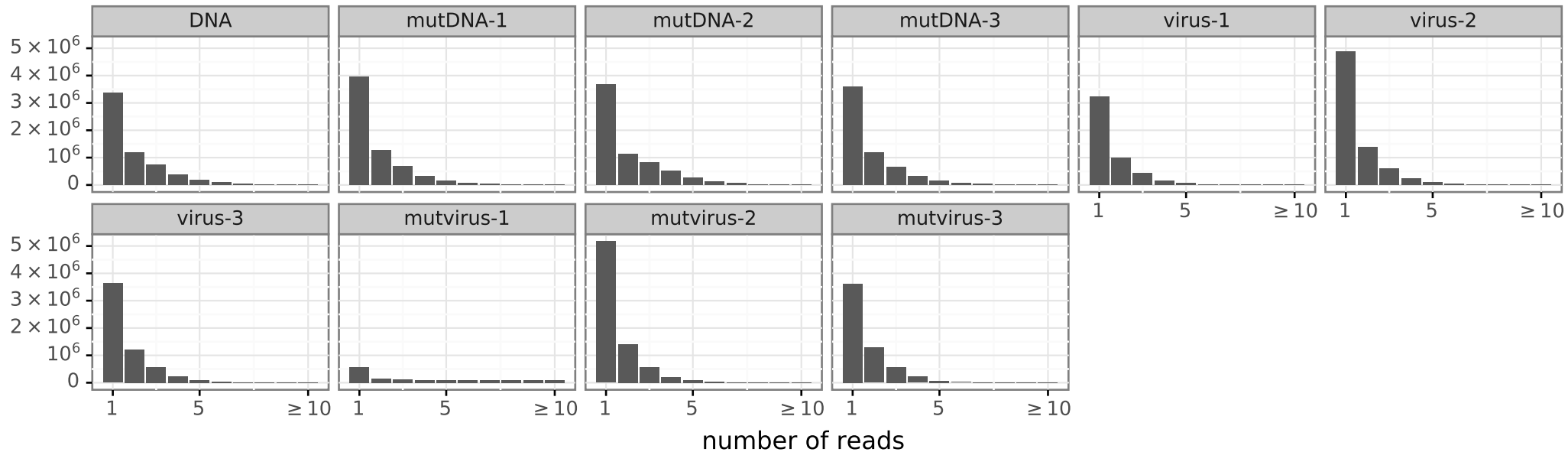

Supplement: Supplementary file 1. — Specifically, this file contains a Jupyter notebook that performs the analysis, all required input data, and all reasonably sized output files. The Jupyter notebook downloads the deep sequencing data, processes it with the dms_tools2 software (Bloom, 2015, https://jbloomlab.github.io/dms_tools2/), and also performs a variety of downstream analyses that generate most of the figures for this paper. [file elife-34420-supp1.zip › analysis_code/results/codoncounts/BF520/summary_readsperbc.pdf]

number of reads

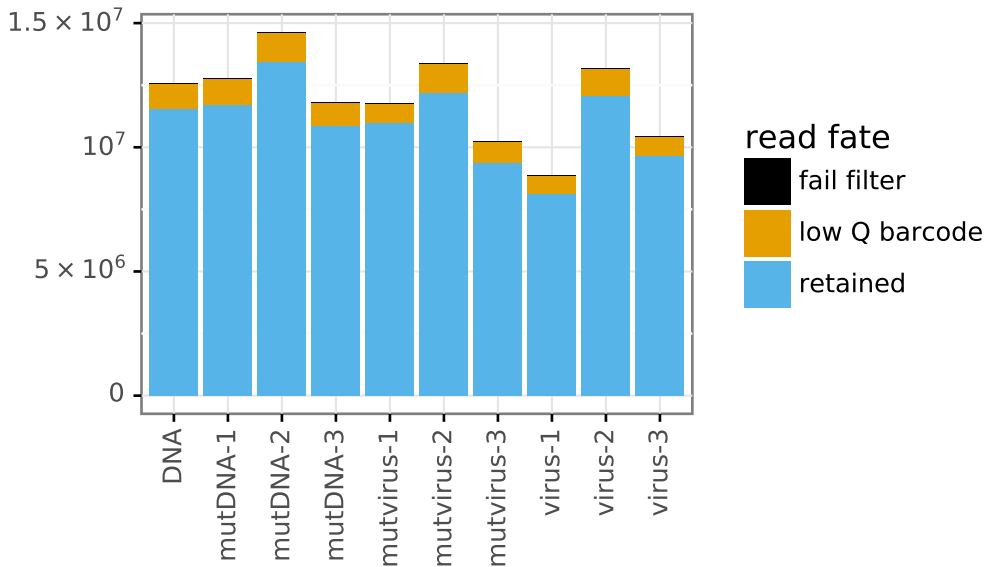

Supplement: Supplementary file 1. — Specifically, this file contains a Jupyter notebook that performs the analysis, all required input data, and all reasonably sized output files. The Jupyter notebook downloads the deep sequencing data, processes it with the dms_tools2 software (Bloom, 2015, https://jbloomlab.github.io/dms_tools2/), and also performs a variety of downstream analyses that generate most of the figures for this paper. [file elife-34420-supp1.zip › analysis_code/results/codoncounts/BF520/summary_readstats.pdf]

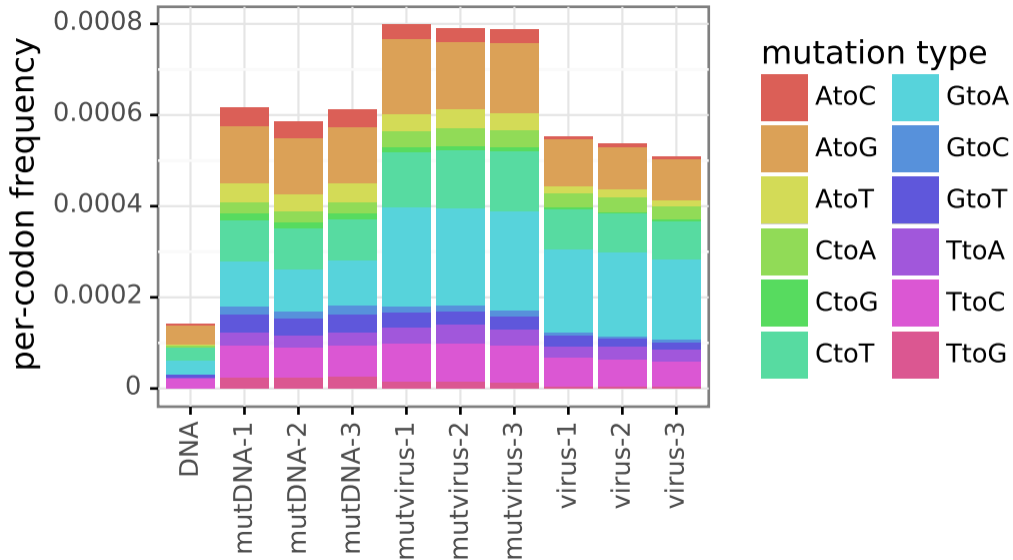

Supplement: Supplementary file 1. — Specifically, this file contains a Jupyter notebook that performs the analysis, all required input data, and all reasonably sized output files. The Jupyter notebook downloads the deep sequencing data, processes it with the dms_tools2 software (Bloom, 2015, https://jbloomlab.github.io/dms_tools2/), and also performs a variety of downstream analyses that generate most of the figures for this paper. [file elife-34420-supp1.zip › analysis_code/results/codoncounts/BF520/summary_singlentchanges.pdf]

number of barcodes

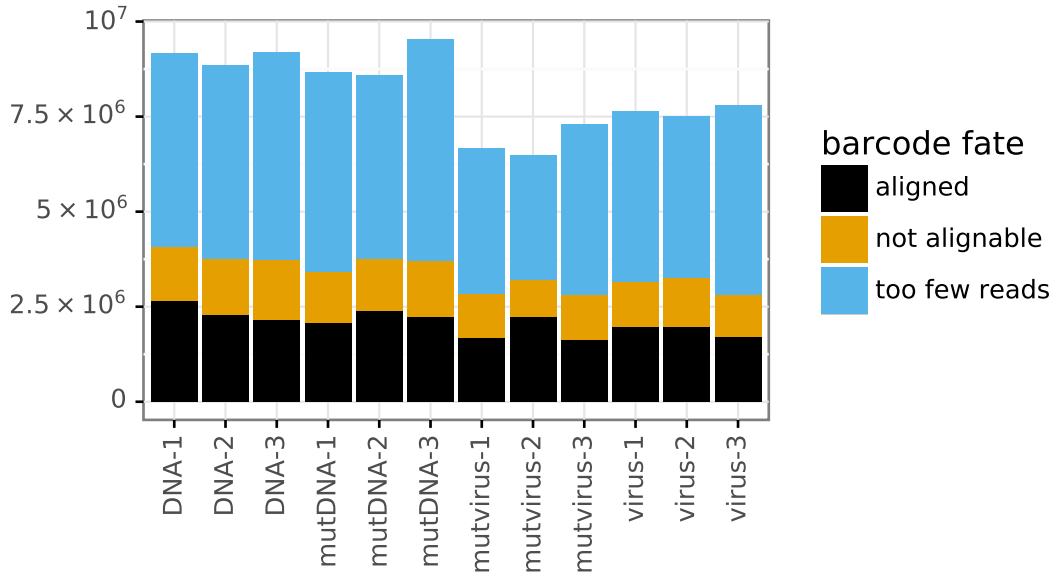

Supplement: Supplementary file 1. — Specifically, this file contains a Jupyter notebook that performs the analysis, all required input data, and all reasonably sized output files. The Jupyter notebook downloads the deep sequencing data, processes it with the dms_tools2 software (Bloom, 2015, https://jbloomlab.github.io/dms_tools2/), and also performs a variety of downstream analyses that generate most of the figures for this paper. [file elife-34420-supp1.zip › analysis_code/results/codoncounts/BG505/summary_bcstats.pdf]

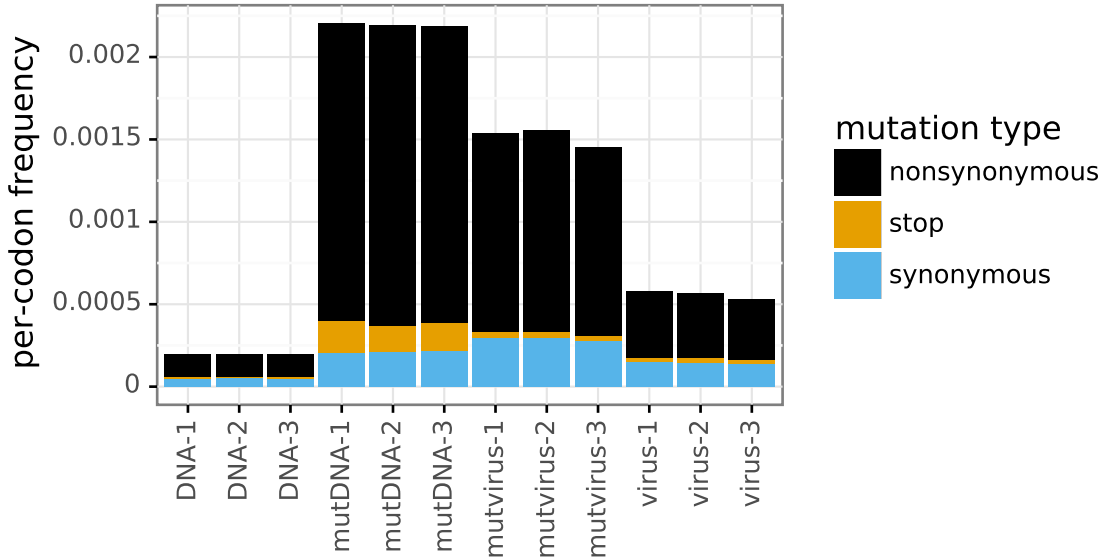

Supplement: Supplementary file 1. — Specifically, this file contains a Jupyter notebook that performs the analysis, all required input data, and all reasonably sized output files. The Jupyter notebook downloads the deep sequencing data, processes it with the dms_tools2 software (Bloom, 2015, https://jbloomlab.github.io/dms_tools2/), and also performs a variety of downstream analyses that generate most of the figures for this paper. [file elife-34420-supp1.zip › analysis_code/results/codoncounts/BG505/summary_codonmuttypes.pdf]

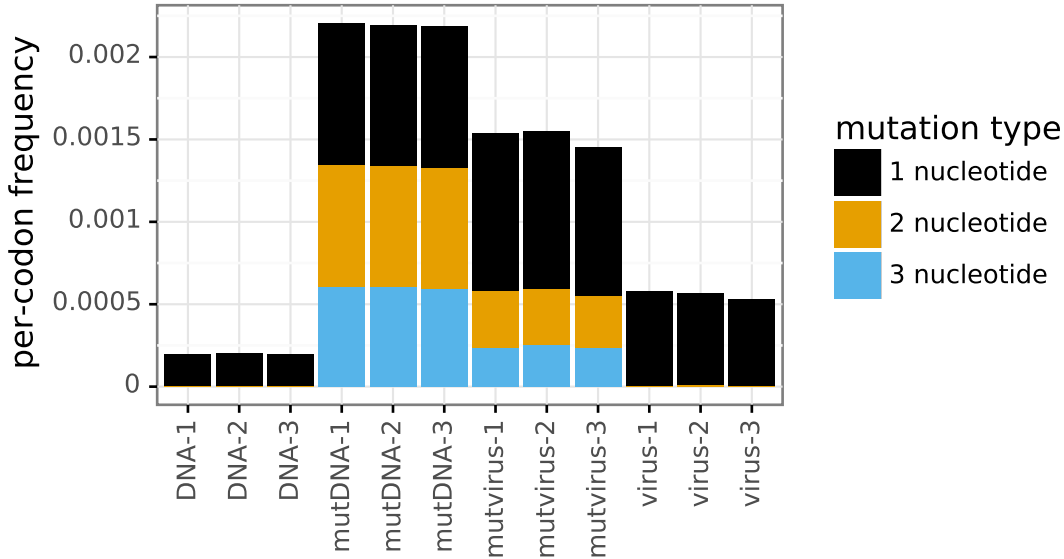

Supplement: Supplementary file 1. — Specifically, this file contains a Jupyter notebook that performs the analysis, all required input data, and all reasonably sized output files. The Jupyter notebook downloads the deep sequencing data, processes it with the dms_tools2 software (Bloom, 2015, https://jbloomlab.github.io/dms_tools2/), and also performs a variety of downstream analyses that generate most of the figures for this paper. [file elife-34420-supp1.zip › analysis_code/results/codoncounts/BG505/summary_codonntchanges.pdf]

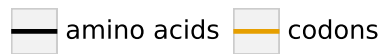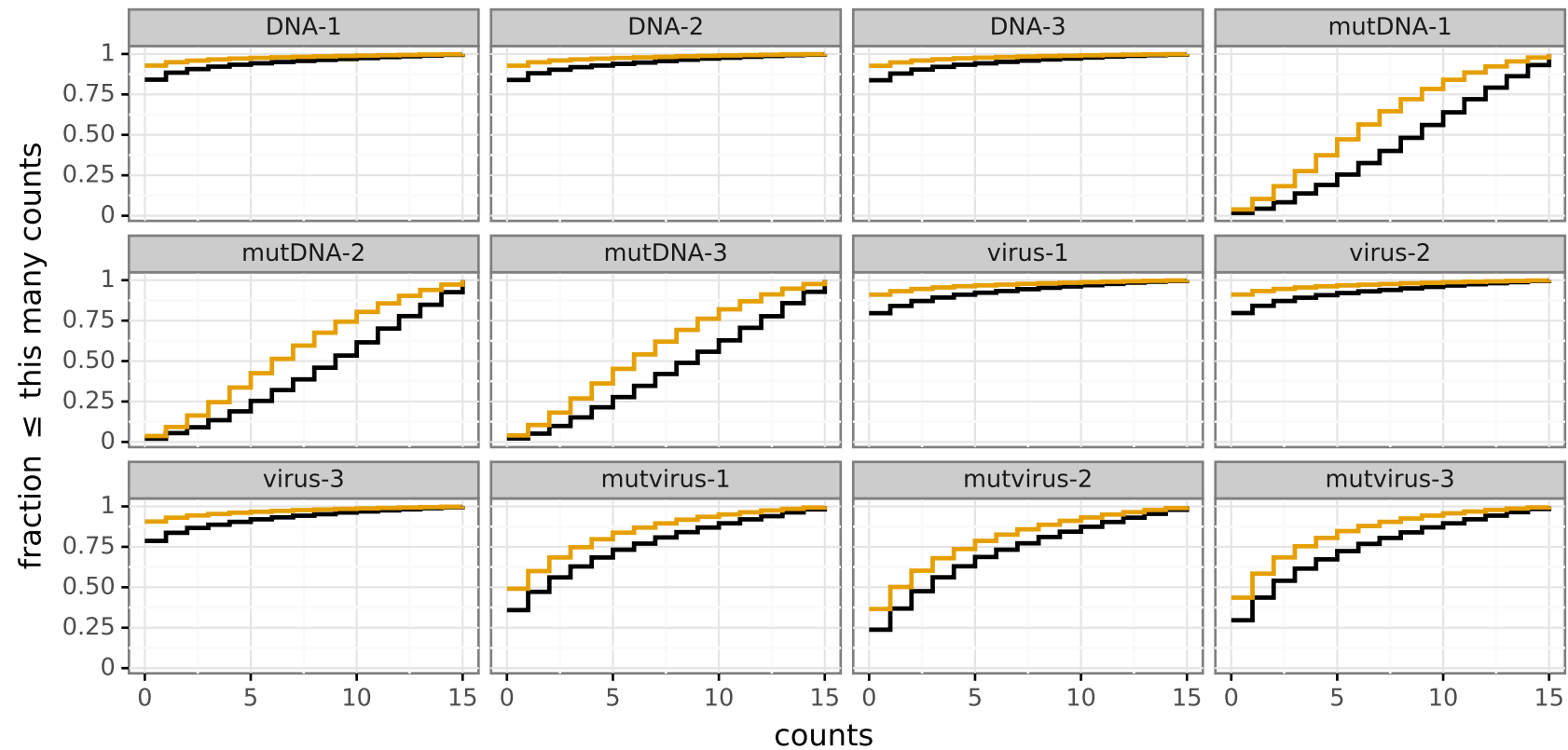

Supplement: Supplementary file 1. — Specifically, this file contains a Jupyter notebook that performs the analysis, all required input data, and all reasonably sized output files. The Jupyter notebook downloads the deep sequencing data, processes it with the dms_tools2 software (Bloom, 2015, https://jbloomlab.github.io/dms_tools2/), and also performs a variety of downstream analyses that generate most of the figures for this paper. [file elife-34420-supp1.zip › analysis_code/results/codoncounts/BG505/summary_cumulmutcounts.pdf]

number of counts

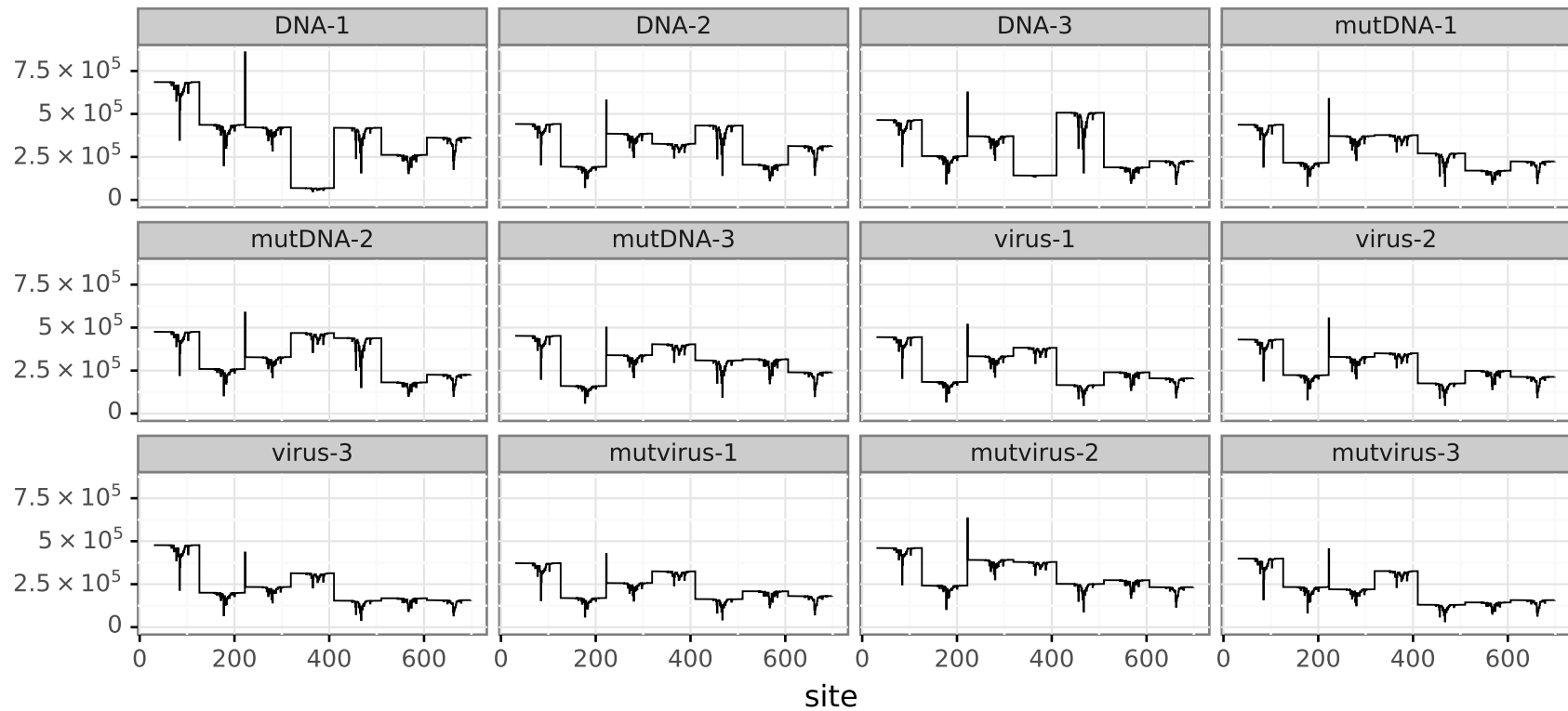

Supplement: Supplementary file 1. — Specifically, this file contains a Jupyter notebook that performs the analysis, all required input data, and all reasonably sized output files. The Jupyter notebook downloads the deep sequencing data, processes it with the dms_tools2 software (Bloom, 2015, https://jbloomlab.github.io/dms_tools2/), and also performs a variety of downstream analyses that generate most of the figures for this paper. [file elife-34420-supp1.zip › analysis_code/results/codoncounts/BG505/summary_depth.pdf]

mutation frequency

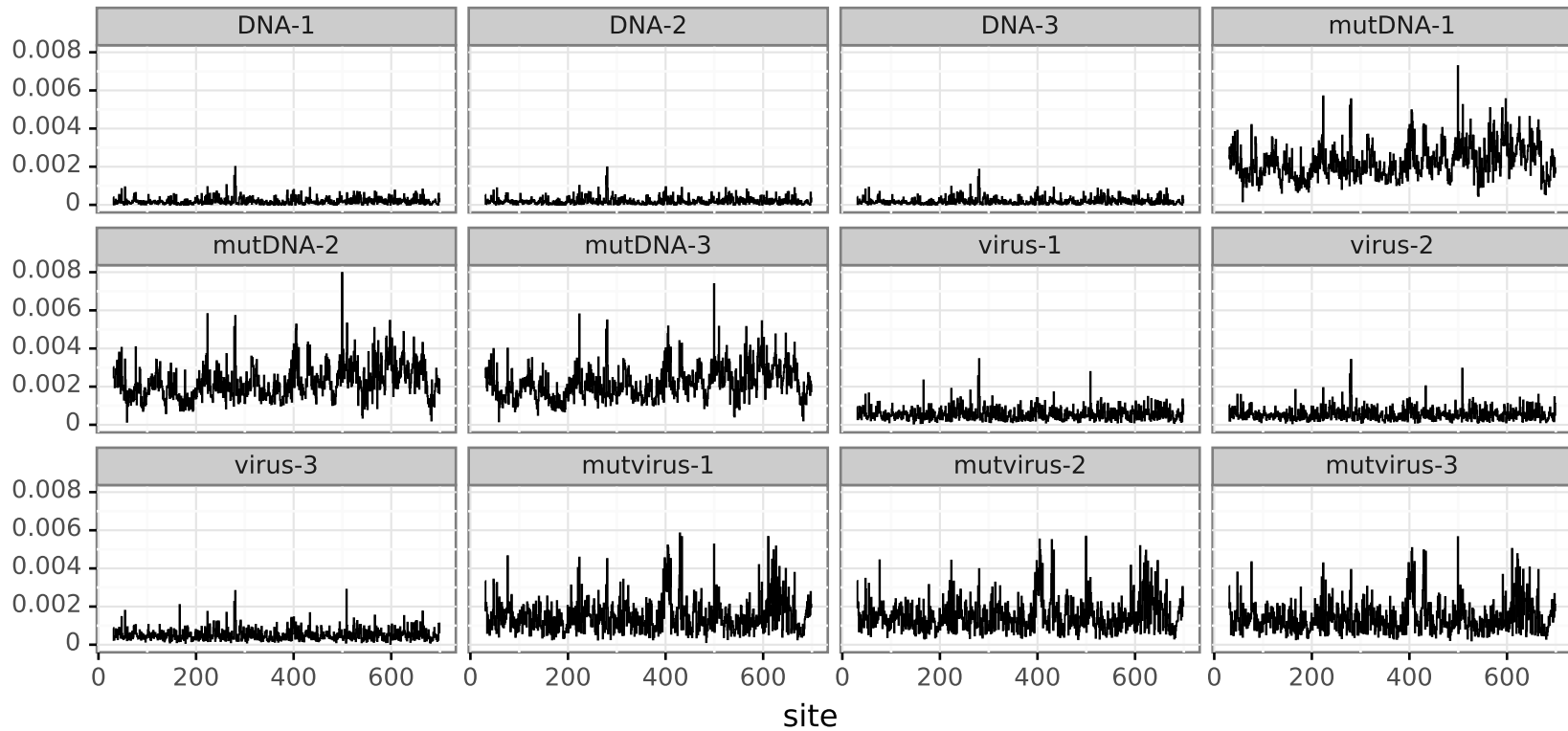

Supplement: Supplementary file 1. — Specifically, this file contains a Jupyter notebook that performs the analysis, all required input data, and all reasonably sized output files. The Jupyter notebook downloads the deep sequencing data, processes it with the dms_tools2 software (Bloom, 2015, https://jbloomlab.github.io/dms_tools2/), and also performs a variety of downstream analyses that generate most of the figures for this paper. [file elife-34420-supp1.zip › analysis_code/results/codoncounts/BG505/summary_mutfreq.pdf]

number of barcodes

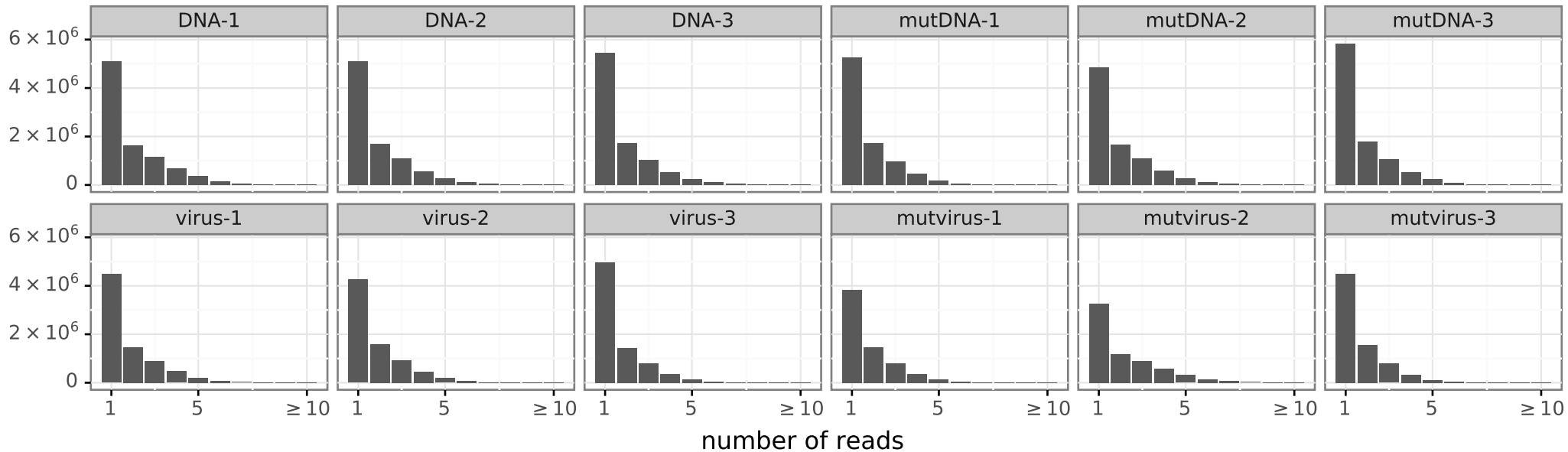

Supplement: Supplementary file 1. — Specifically, this file contains a Jupyter notebook that performs the analysis, all required input data, and all reasonably sized output files. The Jupyter notebook downloads the deep sequencing data, processes it with the dms_tools2 software (Bloom, 2015, https://jbloomlab.github.io/dms_tools2/), and also performs a variety of downstream analyses that generate most of the figures for this paper. [file elife-34420-supp1.zip › analysis_code/results/codoncounts/BG505/summary_readsperbc.pdf]

number of reads

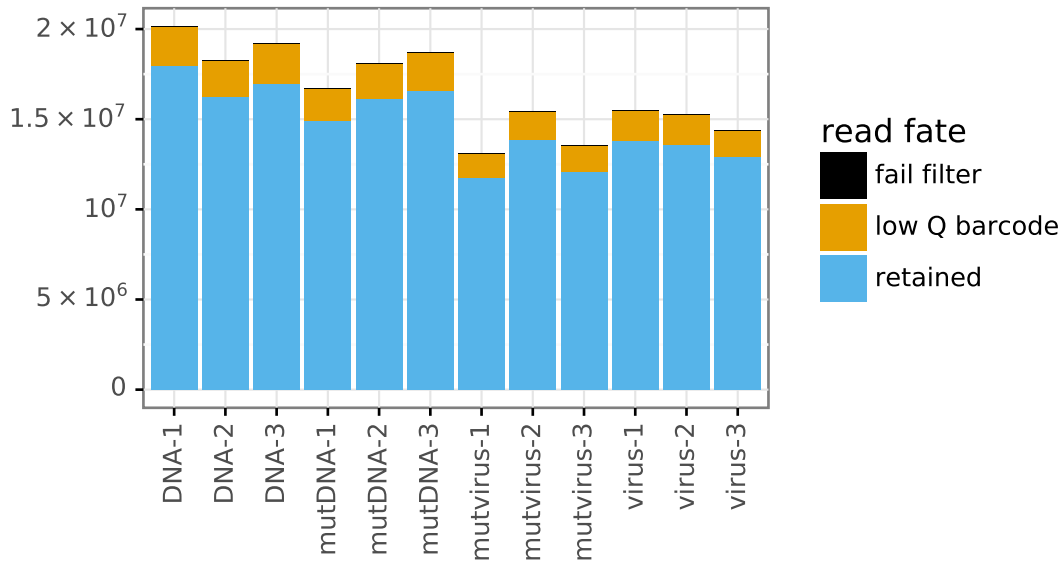

Supplement: Supplementary file 1. — Specifically, this file contains a Jupyter notebook that performs the analysis, all required input data, and all reasonably sized output files. The Jupyter notebook downloads the deep sequencing data, processes it with the dms_tools2 software (Bloom, 2015, https://jbloomlab.github.io/dms_tools2/), and also performs a variety of downstream analyses that generate most of the figures for this paper. [file elife-34420-supp1.zip › analysis_code/results/codoncounts/BG505/summary_readstats.pdf]

per-codon frequency

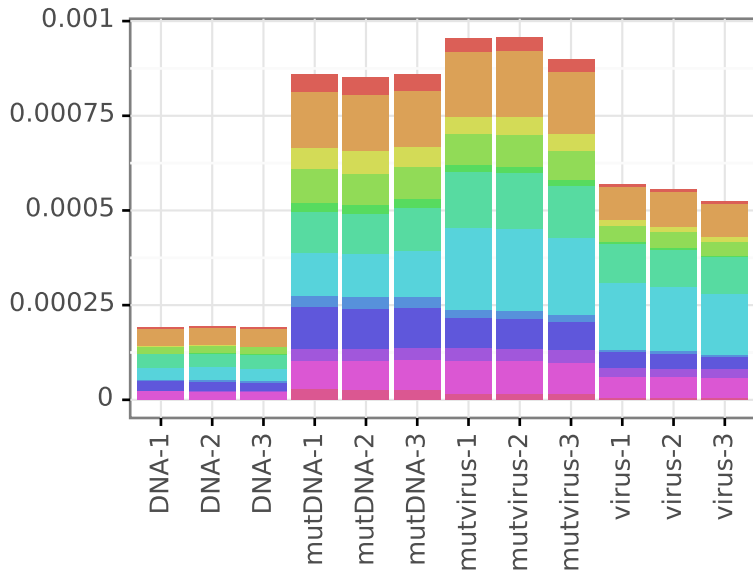

mutation type

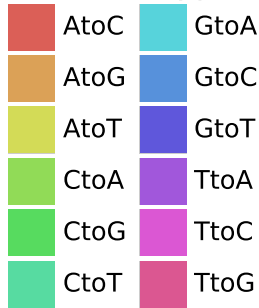

Supplement: Supplementary file 1. — Specifically, this file contains a Jupyter notebook that performs the analysis, all required input data, and all reasonably sized output files. The Jupyter notebook downloads the deep sequencing data, processes it with the dms_tools2 software (Bloom, 2015, https://jbloomlab.github.io/dms_tools2/), and also performs a variety of downstream analyses that generate most of the figures for this paper. [file elife-34420-supp1.zip › analysis_code/results/codoncounts/BG505/summary_singlentchanges.pdf]

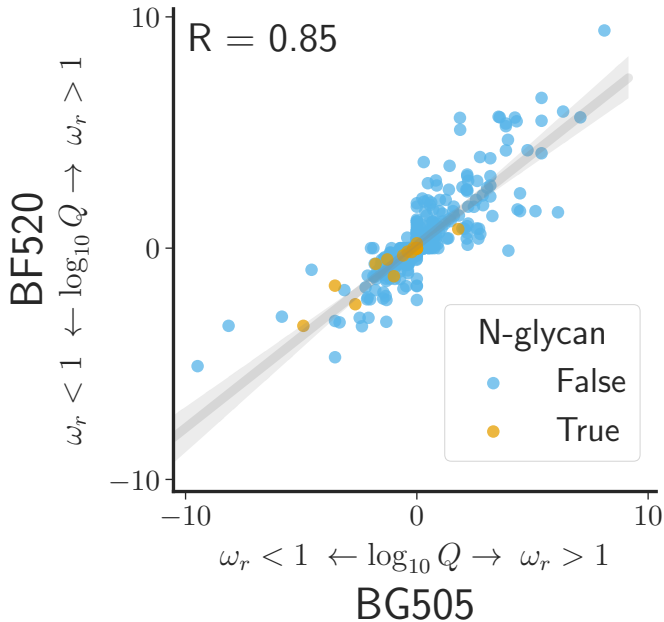

Supplement: Supplementary file 1. — Specifically, this file contains a Jupyter notebook that performs the analysis, all required input data, and all reasonably sized output files. The Jupyter notebook downloads the deep sequencing data, processes it with the dms_tools2 software (Bloom, 2015, https://jbloomlab.github.io/dms_tools2/), and also performs a variety of downstream analyses that generate most of the figures for this paper. [file elife-34420-supp1.zip › analysis_code/results/phydms_analysis/diversifying_selection_corr.pdf]
