# Supplementary material for: Mapping mutational effects along the evolutionary landscape of HIV envelope
Source: eLife. 2018 Mar 28;7:e34420. doi: 10.7554/eLife.34420 (PMC5910023; doi:10.7554/eLife.34420)
Supplement: Supplementary file 2. — The actual notebook is in Supplementary file 1, but if you just want to look at the analysis rather than run it, then you may prefer this file instead. In particular, the notebook contains plots detailing the deep sequencing data analysis as generated using the dms_tools2 software (Bloom, 2015, https://jbloomlab.github.io/dms_tools2/). [file elife-34420-supp2.zip › elife-34420-supp2-v3.html]

analysis\_notebook


# Table of Contents

- Comparing mutational effects among Env homologs
  - Overview
  - Import modules and define common paths
  - Env sequences and numbering
    - Read in Env sequences
    - Create mapping from sequential to HXB2 numbering
  - Download deep sequencing data from the Sequence Read Archive
  - Align deep sequencing reads and count mutations
    - Barcoded-subamplicon alignment
    - Plots summarizing read alignments
      - Number of aligned reads and barcodes
      - Sequencing depth and mutation frequencies across sequence
      - Average mutation frequencies
      - Completenes of mutation sampling
      - Check for oxidative damage
  - Re-number to HXB2 numbering
  - Amino-acid preferences
    - Estimate the amino-acid preferences
    - Look at the correlations among replicates
  - Comparison to HIV evolution in nature
    - Curating Env alignment
      - Build clade A alignment
      - Mask poorly aligned sites
      - Build masked alignment
      - Plot amino-acid divergence from each homolog
    - Phylogenetic analysis
      - Create masked preference sets
      - Run `phydms`
      - Substitution model parameters and diversifying selection
      - Compare sites of diversifying selection among homologs.
      - Visualize phylogenetic tree
  - Visualize amino-acid preferences
    - Logo plots for entire Env
  - Further analysis of diversifying / purifying selection
    - Logo plots of diversifying / purifying selection sites
    - Glycosylation sites
    - Solvent accessibility of sites of diversifying and purifying selection
  - Compare preferences between homologs
    - Comparison of the Env homologs to each other
      - Compute distances between preferences
      - Plot example sites that illustrate the principle of the preferences comparison
      - Collect information about wildtype amino acids
      - Logo plot of scaled differences between homologs at each site
    - Analyze distribution of preference differences
      - Compute distributions of preference differences
      - Estimate significant differences
      - Plot distributions of shifts
      - Plot preferences for sites with significant shifts
    - Analyze features of sites with shifted preferences
      - Aggregate information on shifts and conservation
      - Get structural information (distance matrix)
      - Shifts and residue / residue-neighbor conservation
      - Shifts and residue / residue neighbor conservation, take two
      - Shifts and structural location
    - Examine "entrenchment"
  - Copy files to paper figures directory

# Comparing mutational effects among Env homologs¶

## Overview¶

This Jupyter notebook compares the effects of mutations between two homologs of HIV Env, the BG505 and BF520 strains.
The goal is to quantify how much the effects of mutations have shifted between the viral strains.

This analysis was done by Hugh Haddox, Adam Dingens, Sarah Hilton, and Jesse Bloom.

## Import modules and define common paths¶

First we import Python modules used throughout the notebook, and define key directory paths.

In [1]:

```
import os
import re
import math
import string
import itertools
import multiprocessing
import glob
import operator
import collections
import pickle

import warnings
warnings.simplefilter('ignore') # ignore warnings

import numpy
import scipy.stats
import pandas
import Bio.SeqIO
import Bio.Seq
import Bio.SeqRecord
import Bio.Phylo
import matplotlib
import matplotlib.pyplot as plt
plt.rc('text', usetex=True)
import seaborn
from IPython.display import display, HTML, Markdown
import joypy
import statsmodels.stats.multitest

import dms_tools2
print("Using dms_tools2 version {0}".format(dms_tools2.__version__))
from dms_tools2 import AAS
from dms_tools2.ipython_utils import showPDF
import dms_tools2.sra
import dms_tools2.utils
import dms_tools2.prefs
import dms_tools2.plot
import dms_tools2.dssp
import dms_tools2.compareprefs
import dms_tools2.protstruct
import dms_tools2.rplot
print(dms_tools2.rplot.versionInfo())
import phydmslib
print("Using phydms version {0}".format(phydmslib.__version__))
import phydmslib.utils

# define directories in which results are placed
resultsdir = './results/'
if not os.path.isdir(resultsdir):
    os.mkdir(resultsdir)
    
# do we use existing program output if already present?
use_existing = 'yes'

# Use up to 15 CPUs, more uses too much memory for Hutch server
ncpus = min(15, multiprocessing.cpu_count())
```

```
Using dms_tools2 version 2.2.4
Using `rpy2` 2.9.2 with ``R`` 4.3
Using phydms version 2.2.dev2
```

## Env sequences and numbering¶

A major complexity is how to number and align the two Env homologs.
For computational purposes, it is often convenient to number the sequences sequentially as 1, 2, ... beginning with the N-terminal methionine.
However, the standard numbering scheme for HIV Env is the HXB2 numbering scheme.
In this notebook, we process the FASTQ files and count the mutations in sequential numbering, and then map these numbers to the HXB2 numbering scheme for all subsequent steps.

In addition, we need to align the Env homologs, and take into account that we only mutagenized a portion of Env (we excluded the N-terminal signal peptide and the C-terminal cytoplasmic tail).

### Read in Env sequences¶

First, we define the files holding the Env coding sequences (these are ./data/BG505\_env.fasta and ./data/BF520\_env.fasta) as well as the regions of these sequences that were mutagenized in sequential 1, 2, ... numbering.

In [2]:

```
# the homologs that we are examining
homologs = ['BG505', 'BF520']

# files with codon sequences
wtseqfiles = dict([(env, './data/{0}_env.fasta'.format(env)) for env in homologs])

# the mutagenized codon sites in 1, 2, ... numbering
# for both this is 31-702 in HXB2 numbering
mutagenizedsites = {
        'BG505':list(range(30, 699 + 1)),
        'BF520':list(range(30, 691 + 1))
        }
```

### Create mapping from sequential to HXB2 numbering¶

Now we read in the aligned sequences of the Env homologs and HXB2.
This alignment was created with mafft and then manually edited by Hugh Haddox in some of the variable loop regions that align very poorly.
The manually edited alignment is in ./data/Env\_protalignment\_manualtweaks.fasta.

In [3]:

```
protalignmentfile = './data/Env_protalignment_manualtweaks.fasta'
alignedprots = dict([(seq.name, str(seq.seq)) for seq in 
        Bio.SeqIO.parse(protalignmentfile, 'fasta')])
```

Now we create files that map each residue in our Env homologs to comparable residue sequential numbering of HXB2.
This creates a mapping to the HXB2 numbering scheme that we will use later.
The renumbering files have columns named:

- *original* (sequential 1, 2, ... numbering of homolog)
- *new* (HXB2 numbering)
- *wildtype* (wildtype amino acid in our homolog)
- *N-glycan* (*True* if the Asn of an N-linked glycosylation motif, *False* otherwise).

In [4]:

```
renumbdir = os.path.join(resultsdir, 'HXB2_numbering')
if not os.path.isdir(renumbdir):
    os.mkdir(renumbdir)

# keyed by homolog, value is renumbering file
renumbfile = {}
    
for env in homologs:
    
    # first build numbering with HXB2 numbers potentially repeated
    hxb2_r = 1
    hxb2_nums = [] # holds HXB2 numbers
    wildtype_aas = []
    nglycan = []
    alignedenv = alignedprots[env]
    envlen = len(alignedenv)
    for (r, (hxb2_aa, aa)) in enumerate(zip(alignedprots['HXB2'], alignedenv)):
        if aa != '-':
            hxb2_nums.append(str(hxb2_r - int(hxb2_aa == '-')))
            wildtype_aas.append(aa)
            if aa == 'N' and r + 2 < envlen and alignedenv[r + 2] in {'S', 'T'}:
                nglycan.append(True)
            else:
                nglycan.append(False)
        if hxb2_aa != '-':
            hxb2_r += 1
            
    # now take all runs of HXB2 numbers and make them 10, 10a, 10b, etc...
    for num in set(hxb2_nums):
        n_num = hxb2_nums.count(num)
        firstnum = hxb2_nums.index(num)
        for i in range(n_num - 1):
            hxb2_nums[firstnum + i + 1] = num + string.ascii_lowercase[i]
                
    # now write renumbering file
    renumbfile[env] = os.path.join(renumbdir, '{0}_to_HXB2.csv'.format(env))
    print("Writing HXB2 renumbering file for {0} to {1}".format(
            env, renumbfile[env]))
    with open(renumbfile[env], 'w') as f:
        f.write('original,new,wildtype,N-glycan\n')
        for (r, new) in enumerate(hxb2_nums):
            f.write('{0},{1},{2},{3}\n'.format(r + 1, new, wildtype_aas[r],
                    nglycan[r]))
```

```
Writing HXB2 renumbering file for BG505 to ./results/HXB2_numbering/BG505_to_HXB2.csv
Writing HXB2 renumbering file for BF520 to ./results/HXB2_numbering/BF520_to_HXB2.csv
```

## Download deep sequencing data from the Sequence Read Archive¶

The deep sequencing reads have been submitted to the Sequence Read Archive (SRA), so we download them from there:

- The BF520 reads have the BioSample Accession number SAMN06313000 and BioProject number PRJNA371844.
- The BG505 reads have the BioSample Accession number SAMN07718028 and BioProject number PRJNA412552.

We download these files using the dms\_tools2.sra.fastqFromSRA function from the dms\_tools2 Python API. Note that the call to this function below uses two external programs that you must ensure are installed on the computer that you are using:

1. The fastq-dump program from the SRA Toolkit. If you do not already have this toolkit installed, you will need to install it by following these instructions. You need a relatively recent version.
2. The Aspera Connect program for rapid downloads. You need both the executable `ascp` and an Aspera Key file. Installing Aspera Connect and a key can be somewhat complex, so if you do not want to do this then just set `aspera=None` in the command below and `fastq-dump` will do the downloads (albeit more slowly).

For each homolog, there are the following samples:

- the wildtype plasmid controls (*DNA*)
- the mutant plasmid DNA libraries (*mutDNA*)
- the wildtype virus controls (*virus*)
- the mutant virus samples (*mutvirus*)

There are three replicates of all of these samples for each homolog, except that the BF520 homolog only has one *DNA* control.

In [5]:

```
# specify names of all samples to download
samples = pandas.DataFrame.from_records(
        [('BF520', 'DNA', 'SRR5241717'),
         ('BF520', 'mutDNA-1', 'SRR5241726'),
         ('BF520', 'mutDNA-2', 'SRR5241725'),
         ('BF520', 'mutDNA-3', 'SRR5241724'),
         ('BF520', 'virus-1', 'SRR5241716'),
         ('BF520', 'virus-2', 'SRR5241715'),
         ('BF520', 'virus-3', 'SRR5241714'),
         ('BF520', 'mutvirus-1', 'SRR5241723'),
         ('BF520', 'mutvirus-2', 'SRR5241721'),
         ('BF520', 'mutvirus-3', 'SRR5241719'),
         ('BG505', 'DNA-1', 'SRR6117033'),
         ('BG505', 'DNA-2', 'SRR6117029'),
         ('BG505', 'DNA-3', 'SRR6117027'),
         ('BG505', 'mutDNA-1', 'SRR6117032'),
         ('BG505', 'mutDNA-2', 'SRR6117028'),
         ('BG505', 'mutDNA-3', 'SRR6117026'),
         ('BG505', 'virus-1', 'SRR6117034'),
         ('BG505', 'virus-2', 'SRR6117030'),
         ('BG505', 'virus-3', 'SRR6117024'),
         ('BG505', 'mutvirus-1', 'SRR6117035'),
         ('BG505', 'mutvirus-2', 'SRR6117031'),
         ('BG505', 'mutvirus-3', 'SRR6117025')
        ],
        columns=['homolog', 'sample_name', 'run']
        )
samples['name'] = samples['homolog'] + '-' + samples['sample_name']

# download to this directory
fastqdir = os.path.join(resultsdir, 'FASTQ_files/')

print("Downloading FASTQ files from the SRA...")
dms_tools2.sra.fastqFromSRA(
        samples=samples,
        fastq_dump='fastq-dump', # valid path to this program on the Hutch server
        fastqdir=fastqdir,
        aspera=(
            '/app/aspera-connect/3.5.1/bin/ascp', # valid path to ascp on Hutch server
            '/app/aspera-connect/3.5.1/etc/asperaweb_id_dsa.openssh' # Aspera key on Hutch server
            ),
        )
print("Here are the names of the downloaded files now found in {0}".format(fastqdir))
display(HTML(samples.to_html(index=False)))
```

```
Downloading FASTQ files from the SRA...
Here are the names of the downloaded files now found in ./results/FASTQ_files/
```

| homolog | sample\_name | run | name | R1 | R2 |
| --- | --- | --- | --- | --- | --- |
| BF520 | DNA | SRR5241717 | BF520-DNA | BF520-DNA\_R1.fastq.gz | BF520-DNA\_R2.fastq.gz |
| BF520 | mutDNA-1 | SRR5241726 | BF520-mutDNA-1 | BF520-mutDNA-1\_R1.fastq.gz | BF520-mutDNA-1\_R2.fastq.gz |
| BF520 | mutDNA-2 | SRR5241725 | BF520-mutDNA-2 | BF520-mutDNA-2\_R1.fastq.gz | BF520-mutDNA-2\_R2.fastq.gz |
| BF520 | mutDNA-3 | SRR5241724 | BF520-mutDNA-3 | BF520-mutDNA-3\_R1.fastq.gz | BF520-mutDNA-3\_R2.fastq.gz |
| BF520 | virus-1 | SRR5241716 | BF520-virus-1 | BF520-virus-1\_R1.fastq.gz | BF520-virus-1\_R2.fastq.gz |
| BF520 | virus-2 | SRR5241715 | BF520-virus-2 | BF520-virus-2\_R1.fastq.gz | BF520-virus-2\_R2.fastq.gz |
| BF520 | virus-3 | SRR5241714 | BF520-virus-3 | BF520-virus-3\_R1.fastq.gz | BF520-virus-3\_R2.fastq.gz |
| BF520 | mutvirus-1 | SRR5241723 | BF520-mutvirus-1 | BF520-mutvirus-1\_R1.fastq.gz | BF520-mutvirus-1\_R2.fastq.gz |
| BF520 | mutvirus-2 | SRR5241721 | BF520-mutvirus-2 | BF520-mutvirus-2\_R1.fastq.gz | BF520-mutvirus-2\_R2.fastq.gz |
| BF520 | mutvirus-3 | SRR5241719 | BF520-mutvirus-3 | BF520-mutvirus-3\_R1.fastq.gz | BF520-mutvirus-3\_R2.fastq.gz |
| BG505 | DNA-1 | SRR6117033 | BG505-DNA-1 | BG505-DNA-1\_R1.fastq.gz | BG505-DNA-1\_R2.fastq.gz |
| BG505 | DNA-2 | SRR6117029 | BG505-DNA-2 | BG505-DNA-2\_R1.fastq.gz | BG505-DNA-2\_R2.fastq.gz |
| BG505 | DNA-3 | SRR6117027 | BG505-DNA-3 | BG505-DNA-3\_R1.fastq.gz | BG505-DNA-3\_R2.fastq.gz |
| BG505 | mutDNA-1 | SRR6117032 | BG505-mutDNA-1 | BG505-mutDNA-1\_R1.fastq.gz | BG505-mutDNA-1\_R2.fastq.gz |
| BG505 | mutDNA-2 | SRR6117028 | BG505-mutDNA-2 | BG505-mutDNA-2\_R1.fastq.gz | BG505-mutDNA-2\_R2.fastq.gz |
| BG505 | mutDNA-3 | SRR6117026 | BG505-mutDNA-3 | BG505-mutDNA-3\_R1.fastq.gz | BG505-mutDNA-3\_R2.fastq.gz |
| BG505 | virus-1 | SRR6117034 | BG505-virus-1 | BG505-virus-1\_R1.fastq.gz | BG505-virus-1\_R2.fastq.gz |
| BG505 | virus-2 | SRR6117030 | BG505-virus-2 | BG505-virus-2\_R1.fastq.gz | BG505-virus-2\_R2.fastq.gz |
| BG505 | virus-3 | SRR6117024 | BG505-virus-3 | BG505-virus-3\_R1.fastq.gz | BG505-virus-3\_R2.fastq.gz |
| BG505 | mutvirus-1 | SRR6117035 | BG505-mutvirus-1 | BG505-mutvirus-1\_R1.fastq.gz | BG505-mutvirus-1\_R2.fastq.gz |
| BG505 | mutvirus-2 | SRR6117031 | BG505-mutvirus-2 | BG505-mutvirus-2\_R1.fastq.gz | BG505-mutvirus-2\_R2.fastq.gz |
| BG505 | mutvirus-3 | SRR6117025 | BG505-mutvirus-3 | BG505-mutvirus-3\_R1.fastq.gz | BG505-mutvirus-3\_R2.fastq.gz |

## Align deep sequencing reads and count mutations¶

We used barcoded-subamplicon sequencing to deep sequence the above samples.
So we need to align the reads to count mutations.

### Barcoded-subamplicon alignment¶

We use dms2\_batch\_bcsubamp to process the FASTQ files to counts of each mutant codon at each site.
Note that we create a site mask so that the alignments are only done for the mutagenized sites.

In [6]:

```
# alignment specs for each homolog
alignspecs = {
        'BG505':' '.join(
                   ['87,375,39,36',
                    '376,666,36,39',
                    '663,954,33,41',
                    '955,1228,33,37',
                    '1228,1527,34,35',
                    '1527,1815,32,39',
                    '1816,2098,36,41']),
        'BF520':' '.join(
                   ['88,417,40,39', 
                    '418,753,39,35',
                    '754,1092,36,39',
                    '1093,1447,35,29',
                    '1407,1758,35,32',
                    '1759,2097,30,36'])
        }

# read trimming for each homolog
r1trim = {'BG505':'200', 'BF520':'205'}
r2trim = {'BG505':'170', 'BF520':'212'}

# define counts directory containing output for each homolog
basecountsdir = os.path.join(resultsdir, 'codoncounts')
if not os.path.isdir(basecountsdir):
    os.mkdir(basecountsdir)
countsdir = {}
for env in homologs:
    countsdir[env] = os.path.join(basecountsdir, env)
    if not os.path.isdir(countsdir[env]):
        os.mkdir(countsdir[env])

# align each homolog
for env in homologs:
    print('\nAligning reads and counting mutations for {0}, '
          'placing results in {1}'.format(env, countsdir[env]))
    
    countsbatchfile = os.path.join(countsdir[env], 'batch.csv')
    print("Here is the batch file that we use as input:")
    countsbatch = (samples.query('homolog == @env')
                          [['sample_name', 'R1']]
                          .rename(columns={'sample_name':'name'})
                          )
    display(HTML(countsbatch.to_html(index=False)))
    countsbatch.to_csv(countsbatchfile, index=False)
    
    # make a sitemask that includes only mutagenized sites
    sitemask = os.path.join(countsdir[env], 'sitemask.csv')
    with open(sitemask, 'w') as f:
        f.write('\n'.join(map(str, ['site'] + mutagenizedsites[env])))

    print('\nNow running dms2_batch_bcsubamp...')
    log = !dms2_batch_bcsubamp \
            --batchfile {countsbatchfile} \
            --refseq {wtseqfiles[env]} \
            --alignspecs {alignspecs[env]} \
            --outdir {countsdir[env]} \
            --summaryprefix summary \
            --R1trim {r1trim[env]} \
            --R2trim {r2trim[env]} \
            --fastqdir {fastqdir} \
            --ncpus {ncpus} \
            --sitemask {sitemask} \
            --use_existing {use_existing} 
    print("Completed dms2_batch_bcsubamp.")
```

```
Aligning reads and counting mutations for BG505, placing results in ./results/codoncounts/BG505
Here is the batch file that we use as input:
```

| name | R1 |
| --- | --- |
| DNA-1 | BG505-DNA-1\_R1.fastq.gz |
| DNA-2 | BG505-DNA-2\_R1.fastq.gz |
| DNA-3 | BG505-DNA-3\_R1.fastq.gz |
| mutDNA-1 | BG505-mutDNA-1\_R1.fastq.gz |
| mutDNA-2 | BG505-mutDNA-2\_R1.fastq.gz |
| mutDNA-3 | BG505-mutDNA-3\_R1.fastq.gz |
| virus-1 | BG505-virus-1\_R1.fastq.gz |
| virus-2 | BG505-virus-2\_R1.fastq.gz |
| virus-3 | BG505-virus-3\_R1.fastq.gz |
| mutvirus-1 | BG505-mutvirus-1\_R1.fastq.gz |
| mutvirus-2 | BG505-mutvirus-2\_R1.fastq.gz |
| mutvirus-3 | BG505-mutvirus-3\_R1.fastq.gz |

```
Now running dms2_batch_bcsubamp...
Completed dms2_batch_bcsubamp.

Aligning reads and counting mutations for BF520, placing results in ./results/codoncounts/BF520
Here is the batch file that we use as input:
```

| name | R1 |
| --- | --- |
| DNA | BF520-DNA\_R1.fastq.gz |
| mutDNA-1 | BF520-mutDNA-1\_R1.fastq.gz |
| mutDNA-2 | BF520-mutDNA-2\_R1.fastq.gz |
| mutDNA-3 | BF520-mutDNA-3\_R1.fastq.gz |
| virus-1 | BF520-virus-1\_R1.fastq.gz |
| virus-2 | BF520-virus-2\_R1.fastq.gz |
| virus-3 | BF520-virus-3\_R1.fastq.gz |
| mutvirus-1 | BF520-mutvirus-1\_R1.fastq.gz |
| mutvirus-2 | BF520-mutvirus-2\_R1.fastq.gz |
| mutvirus-3 | BF520-mutvirus-3\_R1.fastq.gz |

```
Now running dms2_batch_bcsubamp...
Completed dms2_batch_bcsubamp.
```

### Plots summarizing read alignments¶

Now let's look at the results of the alignments and codon counts.
These are produced by dms2\_batch\_bcsubamp.
First, we define the prefixes for these plots:

In [7]:

```
countsplotprefix = dict([(env, '{0}/summary_'.format(countsdir[env])) 
        for env in homologs])
```

#### Number of aligned reads and barcodes¶

Below are the number of aligned reads and barcodes for each homolog.
These plots indicate that there are a good number of aligned barcodes for all samples, and that most were sequenced to a fairly appropriate amount.
One (*BF520 mutvirus-1*) appears to have been over-sequenced, but the rest could probably yield even more data if sequenced to greater depth:

In [8]:

```
for env in homologs:
    print("\nRead and barcode stats for {0}:".format(env))
    showPDF([countsplotprefix[env] + suffix for suffix in 
            ['readstats.pdf', 'bcstats.pdf']])
    showPDF(countsplotprefix[env] + 'readsperbc.pdf')
```

```
Read and barcode stats for BG505:
```

```
Read and barcode stats for BF520:
```

#### Sequencing depth and mutation frequencies across sequence¶

Now let's look at the sequencing depth and mutation frequencies across the mutagenized regions of the genes for both homologs.
These seem mostly OK.
Most sites in most samples are sequenced to $>2\times 10^5$ codon counts, with only a few subamplicons dipping below this.
The mutation frequencies generally are pretty uniform for the wildtype controls with the exception of a few spikes for the BF520 wildtype *virus* samples.

In [9]:

```
for env in homologs:
    print("\nDepth and mutation frequency across sequence for {0}:".format(env))
    showPDF(countsplotprefix[env] + 'depth.pdf')
    showPDF(countsplotprefix[env] + 'mutfreq.pdf')
```

```
Depth and mutation frequency across sequence for BG505:
```

```
Depth and mutation frequency across sequence for BF520:
```

#### Average mutation frequencies¶

Now let's look at the **average** mutation frequencies across sites for each homolog.
In addition to looking at the plots, we also print out a dataframe with the average mutation rates.
Overall, these look quite good -- we see the expected purifying selection in the *mutvirus* samples:

In [10]:

```
for env in homologs:
    print("\nAverage mutation frequencies for {0}:".format(env))
    showPDF([countsplotprefix[env] + suffix for suffix in 
            ['codonmuttypes.pdf', 'codonntchanges.pdf']])
```

```
Average mutation frequencies for BG505:
```

```
Average mutation frequencies for BF520:
```

In [11]:

```
mutfreqs = pandas.concat([
        pandas.read_csv(countsplotprefix[env] + 'codonmuttypes.csv')
              .assign(homolog=env)
        for env in homologs])
display(HTML(mutfreqs.to_html(index=False, float_format=lambda x: '{0:.3g}'.format(x))))
```

| homolog | name | nonsynonymous | stop | synonymous |
| --- | --- | --- | --- | --- |
| BG505 | DNA-1 | 0.000137 | 1.01e-05 | 4.94e-05 |
| BG505 | DNA-2 | 0.000137 | 1.1e-05 | 5.04e-05 |
| BG505 | DNA-3 | 0.000134 | 9.55e-06 | 5.01e-05 |
| BG505 | mutDNA-1 | 0.00181 | 0.000188 | 0.00021 |
| BG505 | mutDNA-2 | 0.00182 | 0.000162 | 0.000209 |
| BG505 | mutDNA-3 | 0.0018 | 0.00017 | 0.000217 |
| BG505 | mutvirus-1 | 0.0012 | 3.38e-05 | 0.000296 |
| BG505 | mutvirus-2 | 0.00122 | 3.56e-05 | 0.000296 |
| BG505 | mutvirus-3 | 0.00114 | 3.04e-05 | 0.00028 |
| BG505 | virus-1 | 0.000402 | 2.86e-05 | 0.000149 |
| BG505 | virus-2 | 0.000392 | 2.62e-05 | 0.000146 |
| BG505 | virus-3 | 0.000367 | 2.62e-05 | 0.000139 |
| BF520 | DNA | 9.41e-05 | 6.92e-06 | 4.27e-05 |
| BF520 | mutDNA-1 | 0.00147 | 0.000123 | 0.000166 |
| BF520 | mutDNA-2 | 0.00135 | 0.000107 | 0.000157 |
| BF520 | mutDNA-3 | 0.00142 | 0.000125 | 0.000162 |
| BF520 | mutvirus-1 | 0.000986 | 4.77e-05 | 0.000249 |
| BF520 | mutvirus-2 | 0.000986 | 4.33e-05 | 0.00024 |
| BF520 | mutvirus-3 | 0.000946 | 4.62e-05 | 0.000241 |
| BF520 | virus-1 | 0.00039 | 3.05e-05 | 0.000141 |
| BF520 | virus-2 | 0.000385 | 2.91e-05 | 0.000136 |
| BF520 | virus-3 | 0.000362 | 2.72e-05 | 0.00013 |

Now we calculate the **corrected** mutation average mutation frequencies by subtracting the appropriate error control for each sample:

- The **pre**-selection frequency is *mutDNA* - *DNA*
- The **post**-selection frequency is *mutvirus* - *virus*

Note that since we only have one *DNA* sample for BF520, we have to use that sample as the control for all three BF520 replicates.

We then calculate the percent of each mutation type remaining post-selection.

In [12]:

```
corr_mutfreqs = (mutfreqs
                 .append(pandas.concat( 
                     [mutfreqs.query('homolog == "BF520" and name == "DNA"')
                              .assign(name='DNA-{0}'.format(i + 1)) 
                     for i in range(3)])
                     )
                 .assign(sample=lambda x: x.name.str.split('-').str[0],
                         experiment=lambda x: x.homolog + '-' + x.name.str.split('-').str[1])
                 .dropna()
                 .rename(columns={'nonsynonymous':'nonsyn', 'synonymous':'syn'})
                 .melt(id_vars=['experiment', 'sample'],
                       value_vars=['nonsyn', 'syn', 'stop'],
                       var_name='muttype',
                       value_name='mutfreq')
                 .pivot_table(index=['experiment', 'muttype'], columns='sample', values='mutfreq')
                 .assign(pre=lambda x: x.mutDNA - x.DNA,
                         post=lambda x: x.mutvirus - x.virus)
                 .assign(percent=lambda x: 100 * x.post / x.pre)
                 )

display(HTML(corr_mutfreqs.to_html(float_format=lambda x: '{0:.3g}'.format(x))))
```

|  | sample | DNA | mutDNA | mutvirus | virus | pre | post | percent |
| --- | --- | --- | --- | --- | --- | --- | --- | --- |
| experiment | muttype |  |  |  |  |  |  |  |
| BF520-1 | nonsyn | 9.41e-05 | 0.00147 | 0.000986 | 0.00039 | 0.00138 | 0.000596 | 43.3 |
|  | stop | 6.92e-06 | 0.000123 | 4.77e-05 | 3.05e-05 | 0.000116 | 1.72e-05 | 14.8 |
|  | syn | 4.27e-05 | 0.000166 | 0.000249 | 0.000141 | 0.000124 | 0.000107 | 86.6 |
| BF520-2 | nonsyn | 9.41e-05 | 0.00135 | 0.000986 | 0.000385 | 0.00125 | 0.000601 | 48 |
|  | stop | 6.92e-06 | 0.000107 | 4.33e-05 | 2.91e-05 | 0.0001 | 1.42e-05 | 14.2 |
|  | syn | 4.27e-05 | 0.000157 | 0.00024 | 0.000136 | 0.000114 | 0.000103 | 90.4 |
| BF520-3 | nonsyn | 9.41e-05 | 0.00142 | 0.000946 | 0.000362 | 0.00133 | 0.000584 | 44 |
|  | stop | 6.92e-06 | 0.000125 | 4.62e-05 | 2.72e-05 | 0.000118 | 1.9e-05 | 16.2 |
|  | syn | 4.27e-05 | 0.000162 | 0.000241 | 0.00013 | 0.000119 | 0.000111 | 93.4 |
| BG505-1 | nonsyn | 0.000137 | 0.00181 | 0.0012 | 0.000402 | 0.00167 | 0.000802 | 48 |
|  | stop | 1.01e-05 | 0.000188 | 3.38e-05 | 2.86e-05 | 0.000178 | 5.21e-06 | 2.92 |
|  | syn | 4.94e-05 | 0.00021 | 0.000296 | 0.000149 | 0.00016 | 0.000147 | 91.9 |
| BG505-2 | nonsyn | 0.000137 | 0.00182 | 0.00122 | 0.000392 | 0.00168 | 0.000829 | 49.2 |
|  | stop | 1.1e-05 | 0.000162 | 3.56e-05 | 2.62e-05 | 0.000151 | 9.44e-06 | 6.25 |
|  | syn | 5.04e-05 | 0.000209 | 0.000296 | 0.000146 | 0.000159 | 0.00015 | 94.5 |
| BG505-3 | nonsyn | 0.000134 | 0.0018 | 0.00114 | 0.000367 | 0.00166 | 0.000774 | 46.5 |
|  | stop | 9.55e-06 | 0.00017 | 3.04e-05 | 2.62e-05 | 0.00016 | 4.2e-06 | 2.62 |
|  | syn | 5.01e-05 | 0.000217 | 0.00028 | 0.000139 | 0.000167 | 0.000141 | 84.7 |

#### Completenes of mutation sampling¶

Next we look at cumulative fraction plots telling us how completely the different possible mutations were sampled.
We can see that almost all amino-acid mutations were sampled for both homologs in **each** of the triplicate mutant libraries.

In [13]:

```
for env in homologs:
    print("\nMutation sampling completeness for {0}:".format(env))
    showPDF(countsplotprefix[env] + 'cumulmutcounts.pdf')
```

```
Mutation sampling completeness for BG505:
```

```
Mutation sampling completeness for BF520:
```

#### Check for oxidative damage¶

Oxidative damage is characterized by an excess of `C -> A` or `G -> T` mutations.
We see now evidence of an excess of such mutations.
Instead, as expected, the most common mutations are transitions (`G <-> A` or `C <-> T`).

In [14]:

```
for env in homologs:
    print("\nSingle-nucleotide change types for {0}:".format(env))
    showPDF(countsplotprefix[env] + 'singlentchanges.pdf', width=500)
```

```
Single-nucleotide change types for BG505:
```

```
Single-nucleotide change types for BF520:
```

## Re-number to HXB2 numbering¶

As mentioned above, we want to perform **all subsequent steps** using sites numbered in the HXB2 numbering scheme.
We have already defined renumbering files above to enable this.

Here we create a new results subdirectory that has the re-numbered codon counts for each homolog:

In [15]:

```
baserenumbcountsdir = os.path.join(resultsdir, 'renumbered_codoncounts')
if not os.path.isdir(baserenumbcountsdir):
    os.mkdir(baserenumbcountsdir)

renumbcountsdir = {}
for env in homologs:
    renumbcountsdir[env] = os.path.join(baserenumbcountsdir, env)
    print("Renumber counts for {0} will go in {1}".format(
            env, renumbcountsdir[env]))
    if not os.path.isdir(renumbcountsdir[env]):
        os.mkdir(renumbcountsdir[env])
    dms_tools2.utils.renumberSites(
            renumbfile[env],
            list(glob.glob(os.path.join(countsdir[env], '*codoncounts.csv'))),
            missing='drop',
            outdir=renumbcountsdir[env]
            )
```

```
Renumber counts for BG505 will go in ./results/renumbered_codoncounts/BG505
Renumber counts for BF520 will go in ./results/renumbered_codoncounts/BF520
```

## Amino-acid preferences¶

We now want to use the counts to estimate the amino-acid preferences for each homolog.

### Estimate the amino-acid preferences¶

We do this using the program dms2\_batch\_prefs.

In [16]:

```
prefsdir = os.path.join(resultsdir, 'prefs')
if not os.path.isdir(prefsdir):
    os.mkdir(prefsdir)

# batch file skeleton for dms2_batch_prefs
prefsbatch = pandas.DataFrame(
        columns=['name', 'pre', 'post', 'errpre', 'errpost'],
        data=[('1', 'mutDNA-1', 'mutvirus-1', 'DNA-1', 'virus-1'),
              ('2', 'mutDNA-2', 'mutvirus-2', 'DNA-2', 'virus-2'),
              ('3', 'mutDNA-3', 'mutvirus-3', 'DNA-3', 'virus-3')]
        )

prefsfiles = {} # holds names of the preferences files 
for env in homologs:
    print('\nInferring amino-acid preferences for {0}'.format(env))
    
    envprefsbatch = prefsbatch.copy()
    envprefsbatch['name'] = env + '-' + envprefsbatch['name']
    if env == 'BF520':
        # only one DNA control for this sample
        envprefsbatch['errpre'] = 'DNA'
    print("Here is the batch file for dms2_batch_prefs:")
    display(HTML(envprefsbatch.to_html(index=False)))
    prefsbatchfile = os.path.join(prefsdir, '{0}_batch.csv'.format(env))
    envprefsbatch.to_csv(prefsbatchfile, index=False)
    
    print("Running dms2_batch_prefs...")
    log = !dms2_batch_prefs \
            --indir {renumbcountsdir[env]} \
            --batchfile {prefsbatchfile} \
            --outdir {prefsdir} \
            --summaryprefix {env} \
            --use_existing {use_existing} 
    print("Completed running dms2_batch_prefs")
    
    for name in envprefsbatch.name:
        prefsfiles[name] = os.path.join(prefsdir, name + '_prefs.csv')
    prefsfiles[env + '-avg'] = os.path.join(prefsdir, env + '_avgprefs.csv')
```

```
Inferring amino-acid preferences for BG505
Here is the batch file for dms2_batch_prefs:
```

| name | pre | post | errpre | errpost |
| --- | --- | --- | --- | --- |
| BG505-1 | mutDNA-1 | mutvirus-1 | DNA-1 | virus-1 |
| BG505-2 | mutDNA-2 | mutvirus-2 | DNA-2 | virus-2 |
| BG505-3 | mutDNA-3 | mutvirus-3 | DNA-3 | virus-3 |

```
Running dms2_batch_prefs...
Completed running dms2_batch_prefs

Inferring amino-acid preferences for BF520
Here is the batch file for dms2_batch_prefs:
```

| name | pre | post | errpre | errpost |
| --- | --- | --- | --- | --- |
| BF520-1 | mutDNA-1 | mutvirus-1 | DNA | virus-1 |
| BF520-2 | mutDNA-2 | mutvirus-2 | DNA | virus-2 |
| BF520-3 | mutDNA-3 | mutvirus-3 | DNA | virus-3 |

```
Running dms2_batch_prefs...
Completed running dms2_batch_prefs
```

The preferences are now in the following files (one for each replicate plus one with the average for each homolog).
We do not visually display these in logo plots right now; we will wait to do that until after we have re-scaled them to match the stringency of selection in nature.

In [17]:

```
print("The preferences are in the following files:\n{0}"
        .format('\n'.join(sorted(prefsfiles.values()))))
assert all(map(os.path.isfile, prefsfiles.values()))
```

```
The preferences are in the following files:
./results/prefs/BF520-1_prefs.csv
./results/prefs/BF520-2_prefs.csv
./results/prefs/BF520-3_prefs.csv
./results/prefs/BF520_avgprefs.csv
./results/prefs/BG505-1_prefs.csv
./results/prefs/BG505-2_prefs.csv
./results/prefs/BG505-3_prefs.csv
./results/prefs/BG505_avgprefs.csv
```

### Look at the correlations among replicates¶

Now we look at the correlations of the inferred amino-acid preferences among replicates for each homolog.
The correlations are pretty good, much better than in our original HIV deep mutational scanning of the LAI strain described in Haddox et al (2016).
These improvements may be due to some combination of using different viral strains and various improvements to the deep mutational scanning protocol.
In any case, these improvements are why we will **not** also compare to the old LAI data -- the current data are just much cleaner.

Here are plots showing the correlations between pairs of replicates for the same homolog:

In [18]:

```
prefcorrs = [os.path.join(prefsdir, env + '_prefscorr.pdf') for env in homologs]
showPDF(prefcorrs, width=600)
```

Now here is the correlation between all the replicates of all homologs:

In [19]:

```
allprefscorr = os.path.join(prefsdir, 'allprefscorr.pdf')
names = sorted([name for name in prefsfiles if 'avg' not in name])

# how to color scatter plots depending on which homologs
# this will break for different numbers of homologs
colors = ['darkblue'] * 3 + ['gray'] * 6 + ['darkred'] + ['gray'] * 3 + ['darkred'] * 2
dms_tools2.plot.plotCorrMatrix(
        names=names,
        infiles=[prefsfiles[name] for name in names],
        plotfile=allprefscorr,
        datatype='prefs',
        colors=colors,
        )
showPDF(allprefscorr, width=700)
```

The points are obviously over-plotted on the above plot.
One thing the reviewers asked is whether we could remedy this problem with a contour plot.
Here we make such a plot, but show that it is actually less useful.
The reason is that the density is so heavily piled around the origin that all the density shows up there.

In [20]:

```
allprefscorrcontour = os.path.join(prefsdir, 'allprefscorr_contour.pdf')

dms_tools2.plot.plotCorrMatrix(
        names=names,
        infiles=[prefsfiles[name] for name in names],
        plotfile=allprefscorrcontour,
        datatype='prefs',
        contour=True,
        ncontours=25,
        )
showPDF(allprefscorrcontour, width=700)
```

## Comparison to HIV evolution in nature¶

### Curating Env alignment¶

We curate an alignment of HIV Env coding sequences to use for our phylogenetic analyses.

#### Build clade A alignment¶

First we build an alignment of clade A sequences.
We choose this clade, because that is what our BG505 and BF520 homologs belong to.

1. We start with an Env coding sequence alignment (group M sequences) downloaded from the Los Alamos HIV Database.
   This alignment is in ./data/HIV1\_FLT\_2016\_env\_DNA.fasta.
2. We extract just the **clade A** sequences from this alignment, removing BG505 and BF520 as we will add them back later. We also keep HXB2 as we need it for numbering conversions.
3. We then use phydms\_prepalignment to curate this alignment down to a reasonable number of sequences by using `--minuniqueness` option.
4. We remove HXB2 (which is clade B), and add the exact BG505 and BF520 sequences used in our deep mutational scanning.

In [21]:

```
aligndir = os.path.join(resultsdir, 'alignments')
if not os.path.isdir(aligndir):
    os.mkdir(aligndir)

# downloaded alignment with all group M sequences
allgroupM = 'data/HIV1_FLT_2016_env_DNA.fasta'

# get just clade A, also keep HXB2 for alignments and remove later
allcladeA = os.path.join(aligndir, 'cladeA_' + os.path.basename(allgroupM))
Bio.SeqIO.write(
        [s for s in Bio.SeqIO.parse(allgroupM, 'fasta') if 
                s.name[0] == 'A' or 'HXB2' in s.name],
        allcladeA,
        'fasta'
        )

# use phydms_prepalignment to get reasonable number of clean unique seqs
alignmentfile = os.path.join(aligndir, 'cladeA_alignment.fasta')
log = !phydms_prepalignment \
        {allcladeA} \
        {alignmentfile} \
        HXB2 \
        --minuniqueness {165} \
        --prealigned \
        --purgeseqs BG505 BF520
        
# check HXB2 matches sequence used for numbering conversions above
alignment = list(Bio.SeqIO.parse(alignmentfile, 'fasta'))
hxb2aligned = [s for s in alignment if 'HXB2' in s.name]
assert len(hxb2aligned) == 1, "did not find exactly one HXB2 in alignment"
hxb2alignedprot = hxb2aligned[0].seq.translate()
assert hxb2alignedprot == alignedprots['HXB2'].replace('-', ''),\
        "HXB2 in large alignment doesn't match that used for numbering conversions"
    
# get alignment without HXB2 (which is not clade A)
alignment = [s for s in alignment if 'HXB2' not in s.name]

# now add our homologs to alignment, aligning via numbering schemes defined above
for env in homologs:
    seq = str(Bio.SeqIO.read(wtseqfiles[env], 'fasta').seq)
    # get dict mapping 1, 2, ... HXB2 number to 1, 2, ... numbering of homolog
    renumb = (pandas.read_csv(renumbfile[env])
             .convert_objects(convert_numeric=True)
             .dropna()
             .set_index('new')['original']
             .to_dict()
             )
    alignedseq = []
    for r in range(1, len(hxb2alignedprot) + 1):
        if r in renumb:
            r_env = renumb[r] 
            alignedseq.append(seq[3 * (r_env - 1) : 3 * r_env])
        else:
            alignedseq.append('---')
    alignment.append(Bio.SeqRecord.SeqRecord(Bio.Seq.Seq(''.join(alignedseq)), 
            id=env, description=''))
    
Bio.SeqIO.write(alignment, alignmentfile, 'fasta')
print("Alignment of {0} sequences has been written to {1}".format(
        len(alignment), alignmentfile))
```

```
Alignment of 69 sequences has been written to ./results/alignments/cladeA_alignment.fasta
```

#### Mask poorly aligned sites¶

Some parts of Env align very poorly, particularly in the variable loops.
We therefore mask these sites as improperly aligned sites can confound phylogenetic analyses.

We mask the following sites (selected by Hugh Haddox), all of which are referred to in 1, 2, ... HXB2 numbering.

- sites for which there is no deep mutational scanning data:
  - signal peptide: 1-30
  - cytoplasmic tail: 703-856
- sites in the variable loops V1, V2, V4, and V5 that, by eye, look like low-confidence regions due to many indels:
  - part of V1: 132-151
  - part of V2: 185-190
  - part of V4: 396-413
  - part of V5: 460-465
- sites where >5% of sequences in our clade A alignment have a gap. These sites are computationally determined below.

For the masking, we create a data frame that has the HXB2 number, the corresponding number (if any) for each homolog in HXB2 numbering, and whether or not the site needs to be masked.
We use the same mask for both homologs so that they can all be analyzed in the context of the same alignment.

In [22]:

```
maskfile = os.path.join(aligndir, 'alignment_mask.csv')

# first create data frame that lists all HXB2 sites
sites = list(range(1, len(hxb2alignedprot) + 1)) # list of all sites in HXB2
mask = (pandas.DataFrame({'HXB2':sites}).set_index('HXB2'))
mask.index = mask.index.astype('str') # handle sites as strings as some have letters

# now add columns for each homolog
for env in homologs:
    env_df = (pandas.read_csv(renumbfile[env])
              [['new', 'original']]
              .rename(columns={'new':'HXB2', 'original':env})
              .set_index('HXB2')
              )
    env_df.index = env_df.index.astype('str')
    env_df[env] = env_df[env].astype('str')
    mask = mask.join(env_df)

# specify sites to mask
# first, sites specified manually
maskedsites = set([])
for (x, y) in [(1, 30), (703, 856), (132, 151), (185, 190), (396, 413), (460, 465)]:
    maskedsites = maskedsites.union(set(range(x, y + 1))) 
# now sites with too many gaps
maxgapfrac = 0.05
maxgaps = maxgapfrac * len(alignment) 
for r in sites:
    ngaps = sum([s.seq[3 * (r - 1) : 3 * r] == '---' for s in alignment])
    if ngaps > maxgaps:
        maskedsites.add(r)
# now add gapped sites to df
mask['masked'] = [True if r in maskedsites else False for r in sites] 

# add to mask any sites that don't align to HXB2 for any homolog
for env in homologs:
    mask['masked'] = mask[env].isnull() | mask['masked']
    
# unmaskedsites in HXB2 numbering
unmaskedsites = mask[~mask['masked']].index.astype('int')

print("Masked a total of {0} sites".format(sum(mask['masked'])))
print("Writing the alignment mask to {0}".format(maskfile))
mask.to_csv(maskfile)
```

```
Masked a total of 240 sites
Writing the alignment mask to ./results/alignments/alignment_mask.csv
```

#### Build masked alignment¶

Finally, we need to build an alignment of only the non-masked sites that we can use for subsequent analyses:

In [23]:

```
maskedalignmentfile = os.path.join(aligndir, 'masked_alignment.fasta')

maskedalignment = []
for s in alignment:
    maskedalignment.append(Bio.SeqRecord.SeqRecord(
            Bio.Seq.Seq(''.join([str(s.seq)[3 * (r - 1) : 3 * r] for r in unmaskedsites])),
            id=s.id,
            description='',
            ))
    
print("The masked alignment consists of {0} sequences each {1} codons long."
        .format(len(maskedalignment), len(unmaskedsites)))
Bio.SeqIO.write(maskedalignment, maskedalignmentfile, 'fasta')
print("Writing this alignment to {0}".format(maskedalignmentfile))
```

```
The masked alignment consists of 69 sequences each 616 codons long.
Writing this alignment to ./results/alignments/masked_alignment.fasta
```

#### Plot amino-acid divergence from each homolog¶

First, we compute the pairwise identities between the homologs among both all sites and using just the sites not masked in the alignment.

Then we plot the amino-acid divergence of each sequence in our masked alignment from each homolog, considering only the non-masked sites.

We count an alignment of a gap to a non-gap character as a difference in computing these identities.

In [24]:

```
prots = dict([(s.id, s.seq.translate(gap='-')) for s in alignment])
maskedprots = dict([(s.id, s.seq.translate(gap='-')) for s in maskedalignment])

for (env1, env2) in itertools.combinations(homologs, 2):
    for (aligntype, iprots) in [('full', prots), ('masked', maskedprots)]:
        (s1, s2) = (iprots[env1], iprots[env2])
        sites = [r for r in range(len(s1)) if s1[r] != '-' and s2[r] != '-']
        nident = sum([s1[r] == s2[r] for r in sites])
        print("In {0} alignment, {1} and {2} proteins are {3:.3f} identical ({4} of {5} sites)"
                .format(aligntype, env1, env2, nident / float(len(sites)), nident, len(sites)))

identityplot = os.path.join(aligndir, 'masked_alignment_identity.pdf')

fracidents = dict([(env, []) for env in homologs])
for env in homologs:
    maskedenv = maskedprots[env]
    for (prot_id, prot) in maskedprots.items():
        if prot_id == env:
            continue
        nident = sum([aa == prot[r] for (r, aa) in enumerate(maskedenv)])
        fracident = nident / len(maskedenv)
        fracidents[env].append(fracident)

fracidents_df = (pandas.DataFrame(fracidents)
                 .melt(var_name='Env', value_name='protein identity')
                 )

matplotlib.rc('axes', labelsize=16)
matplotlib.rc('xtick', labelsize=12)
matplotlib.rc('ytick', labelsize=12)
p = seaborn.FacetGrid(fracidents_df, col='Env')
(p.map(plt.hist, 'protein identity', color='gray', bins=12)
        .set(ylabel='number of sequences', yticks=[0, 5, 10, 15])
        .set_titles('to {col_name}', verticalalignment='top', size=14))
p.fig.suptitle('clade A protein identity at alignable sites',
        verticalalignment='bottom', fontsize=14)
p.savefig(identityplot)
showPDF(identityplot, width=500)
```

```
In full alignment, BG505 and BF520 proteins are 0.862 identical (721 of 836 sites)
In masked alignment, BG505 and BF520 proteins are 0.891 identical (549 of 616 sites)
```

### Phylogenetic analysis¶

Now we run phydms for each homolog.
This analysis allows us to compare an experimentally informed codon model (*ExpCM*) to traditional Goldman-Yang (denoted *YNGKP* here) models.
It also allows us to identify sites evolving faster or slower in nature than expected from the experiments.

First we create a directory for this analysis:

In [25]:

```
phydmsdir = os.path.join(resultsdir, 'phydms_analysis/')
if not os.path.isdir(phydmsdir):
    os.mkdir(phydmsdir)
```

#### Create masked preference sets¶

We will create an *ExpCM* with the **average** preferences among replicates for each homolog.
The preferences we have estimated above are in HXB2 numbering for all sites.
The phydms analyses require the alignment sites and preferences to both be in 1, 2, ... numbering.
Because we have only retained some sites after creating our masked alignment, we need to create preferences that sequentially number these masked sites.

In [26]:

```
maskedavgprefs = [os.path.join(phydmsdir, env + '.csv') 
        for env in homologs]

renumb_hxb2_to_mask = os.path.join(phydmsdir, 'renumb_hxb2_to_mask.csv')
(pandas.DataFrame({'original':unmaskedsites, 
                   'new':list(range(1, len(unmaskedsites) + 1))})
        .to_csv(renumb_hxb2_to_mask, index=False)
        )

dms_tools2.utils.renumberSites(
        renumbfile=renumb_hxb2_to_mask,
        infiles=[prefsfiles[env + '-avg'] for env in homologs],
        missing='drop',
        outfiles=maskedavgprefs
        )
```

#### Run `phydms`¶

Now we run phydms\_comprehensive.
Note that this requires that RAxML is installed at the path specified by `--raxml`.

We then print the results of the model comparison for all models.
This shows that the best model is the *ExpCM* with a gamma-distributed $\omega$

In [27]:

```
modelcomparefile = os.path.join(phydmsdir, 'modelcomparison.md')

print("Running phydms...")
if (use_existing != 'yes') or (not os.path.isfile(modelcomparefile)):
    log = !phydms_comprehensive \
            {phydmsdir}/ \
            {maskedalignmentfile} \
            {' '.join(maskedavgprefs)} \
            --raxml raxml \
            --ncpus {ncpus} \
            --gammaomega \
            --omegabysite

print("Here are the phydms model comparison results:")            
display(Markdown(modelcomparefile))
```

```
Running phydms...
Here are the phydms model comparison results:
```

| Model | deltaAIC | LogLikelihood | nParams | ParamValues |
| --- | --- | --- | --- | --- |
| ExpCM\_BF520\_gammaomega | 0.00 | -35218.83 | 7 | alpha\_omega=1.00, beta=2.82, beta\_omega=0.70, kappa=3.29 |
| ExpCM\_BG505\_gammaomega | 269.00 | -35353.33 | 7 | alpha\_omega=0.88, beta=2.14, beta\_omega=0.70, kappa=3.17 |
| YNGKP\_M5 | 3455.10 | -36941.38 | 12 | alpha\_omega=0.55, beta\_omega=0.70, kappa=3.29 |
| averaged\_ExpCM\_BF520\_gammaomega | 3544.84 | -36991.25 | 7 | alpha\_omega=0.57, beta=2.53, beta\_omega=0.70, kappa=3.22 |
| averaged\_ExpCM\_BG505\_gammaomega | 3552.56 | -36995.11 | 7 | alpha\_omega=0.57, beta=1.58, beta\_omega=0.70, kappa=3.22 |
| ExpCM\_BF520 | 3821.76 | -37130.71 | 6 | beta=3.27, kappa=3.12, omega=1.59 |
| ExpCM\_BG505 | 5430.28 | -37934.97 | 6 | beta=2.33, kappa=3.03, omega=1.32 |
| YNGKP\_M0 | 11721.58 | -41075.62 | 11 | kappa=3.04, omega=0.52 |
| averaged\_ExpCM\_BG505 | 11760.84 | -41100.25 | 6 | beta=1.70, kappa=3.13, omega=0.61 |
| averaged\_ExpCM\_BF520 | 11788.22 | -41113.94 | 6 | beta=2.42, kappa=3.14, omega=0.61 |

#### Substitution model parameters and diversifying selection¶

The substitition model comparison immediately above shows that the best model is the *ExpCM* with a gamma-distributed $\omega$ value.
For both homologs, the *ExpCM* with the gamma-distributed $\omega$ greatly outperforms the standard M5 version of the Goldman-Yang (*YNGKP M5*) model.
Models with a single $\omega$ value don't perform as well as ones with gamma-distributed $\omega$, but the *ExpCM* are still better than the comparable M0 Goldman-Yang (*YNGKP M0*) model.

We now perform some additional analyses of the models **with the gamma-distributed $\omega$**.
Specifically, we:

1. Make a nicely formatted table with just those models, and write to a file in LaTex format
2. Extract the stringency parameters for each homolog for these models. These stringency parameters are used to **re-scale the preferences in all subsequent analyses**.

In [28]:

```
# read model comparison to dataframe for gamma-omega models
modelcompare = (phydmslib.utils.modelComparisonDataFrame(
                modelcomparefile, splitparams=True)
        .assign(avgomega=lambda x: x.alpha_omega / x.beta_omega)
        [['Model', 'deltaAIC', 'LogLikelihood', 'nParams', 'beta', 
                'avgomega', 'alpha_omega', 'beta_omega']]
        .rename(columns={'beta':'stringency', 'deltaAIC':'$\Delta$AIC',
                'avgomega':'$\overline{\omega}$', 'kappa':'$\kappa$', 
                'alpha_omega':'$\omega_{\\alpha}$', 
                'beta_omega':'$\omega_{\\beta}$'})
        )

# get only selected models
selectedmodels = ['YNGKP_M5'] + ['ExpCM_' + env + '_gammaomega'
        for env in homologs]
modelcompare = modelcompare[modelcompare['Model'].isin(selectedmodels)]

# get sites where omega_r > or < 1 at given Q-value
qthreshold = 0.05
print("Calling sites with omega > or < 1 at FDR {0}".format(qthreshold))
omegasummary = (pandas.concat([
            pandas.read_csv(os.path.join(phydmsdir, model +
                    '_omegabysite.txt'), sep='\t', comment='#')
                    .assign(Model=model)
                    for model in selectedmodels])
            .query('Q < @qthreshold')
            .assign(omega_gt_1=lambda x: (x['omega'] > 1).astype('int'))
            .assign(omega_lt_1=lambda x: (x['omega'] < 1).astype('int'))
            .groupby('Model')
            [['omega_gt_1', 'omega_lt_1']]
            .sum()
            .reset_index()
            .rename(columns={
                    'omega_gt_1':'nsites $\omega_r > 1$',
                    'omega_lt_1':'nsites $\omega_r < 1$'})
            )
modelcompare = (modelcompare.set_index('Model').join(
            omegasummary.set_index('Model'))
        .reset_index()
        )

# prettify the model names
modelcompare.Model = (modelcompare.Model
        .str.replace('_gammaomega', '')
        .str.replace('YNGKP_M5', 'Goldman-Yang M5')
        .str.replace('_', ' ')
        )

gammamodelcomparefile = os.path.join(phydmsdir, 
        'gamma_omega_modelcomparison.tex')
print("Formatted comparison of models with gamma-distributed omega:")
display(HTML(modelcompare.to_html(index=False, 
        float_format=lambda x: '{0:.1f}'.format(x))))
print("Writing this table to {0}".format(gammamodelcomparefile))
modelcompare.to_latex(gammamodelcomparefile, index=False, escape=False,
        float_format=lambda x: '{0:.1f}'.format(x))

# extract stringency parameters
stringency = (modelcompare.replace(
            ['ExpCM ' + env for env in homologs], homologs)
        .set_index('Model')['stringency']
        [homologs]
        .to_dict()
        )
print("\nPreferences will be re-scaled by these stringency parameters:\n{0}"
        .format('\n'.join(['{0} = {1:.2f}'.format(env, stringency[env]) 
        for env in homologs])))
```

```
Calling sites with omega > or < 1 at FDR 0.05
Formatted comparison of models with gamma-distributed omega:
```

| Model | $\Delta$AIC | LogLikelihood | nParams | stringency | $\overline{\omega}$ | $\omega\_{\alpha}$ | $\omega\_{\beta}$ | nsites $\omega\_r > 1$ | nsites $\omega\_r < 1$ |
| --- | --- | --- | --- | --- | --- | --- | --- | --- | --- |
| ExpCM BF520 | 0.0 | -35218.8 | 7 | 2.8 | 1.4 | 1.0 | 0.7 | 66 | 35 |
| ExpCM BG505 | 269.0 | -35353.3 | 7 | 2.1 | 1.3 | 0.9 | 0.7 | 65 | 53 |
| Goldman-Yang M5 | 3455.1 | -36941.4 | 12 | nan | 0.8 | 0.6 | 0.7 | 14 | 211 |

```
Writing this table to ./results/phydms_analysis/gamma_omega_modelcomparison.tex

Preferences will be re-scaled by these stringency parameters:
BG505 = 2.14
BF520 = 2.82
```

#### Compare sites of diversifying selection among homologs.¶

We want to annotate sites by whether they are the Asn or Ser/Thr in N-linked glycosylation sites.
So first, we get all sites that are part of such a motif in **both** homologs:

In [29]:

```
nglycan = (pandas.concat([pandas.read_csv(renumbfile[env]) for env in homologs])
        .groupby('new')
        .min()
        .reset_index()
        [['new', 'N-glycan']]
        .rename(columns={'new':'site'})
        )

print("{0} sites are N-glycan Asn in all homologs."
        .format(len(nglycan[nglycan['N-glycan']])))
```

```
22 sites are N-glycan Asn in all homologs.
```

We create an `omegabysite.tsv` file for each homolog in HXB2 numbering.
We then create a single dataframe that merges the values for both homologs, and also indicates whether the sites is an N-linked glycosylated Asn.
We then plot the correlation between Q values between homologs.

In [30]:

```
# get omegabysite files in HXB2 numbering
omegabysitefiles = {}
omegabysite = []
for env in homologs:    
    origfile = os.path.join(phydmsdir, 'ExpCM_' + env + '_gammaomega_omegabysite.txt')
    df = pandas.read_csv(origfile, sep='\t', comment='#')
    df['site'] = df['site'].map(dict(enumerate(unmaskedsites, 1)))
    omegabysitefiles[env] = os.path.join(prefsdir, env + '_omegabysite.tsv')
    df.to_csv(omegabysitefiles[env], sep='\t', index=False)
    omegabysite.append(df.assign(Env=env).set_index('site'))

# single data frame with omegabysite values for both homologs in HXB2 numbering
# then create column that is is log10(Q) if omega < 1 and -log10(Q) if omega > 1.
omegabysite = (pandas.concat(omegabysite)
               .reset_index()
               .sort_values('site')
               .reset_index(drop=True)
               .assign(log10Q=lambda x: numpy.log10(x.Q))
               .assign(log10Qdir=lambda x: x.log10Q.where(x.omega < 1, -x.log10Q))
               )
omegabysite['site'] = omegabysite['site'].astype('str')

# add information on N-linked glycosylation sites
omegabysite = omegabysite.merge(nglycan, on='site')

# now plot the correlation of the Q-values between replicates
divselcorr = os.path.join(phydmsdir, 'diversifying_selection_corr.pdf')
if not os.path.isfile(divselcorr):
    df = (omegabysite[['site', 'Env', 'log10Qdir']]
            .pivot(index='site', columns='Env')
            )
    df.columns = df.columns.get_level_values(1)
    df = df.reset_index().merge(nglycan, on='site')
    assert len(homologs) == 2, "plotting needs to be refactored if > 2 homologs"

    seaborn.set_style({'legend.frameon':True})
    g = seaborn.lmplot(x=homologs[0], y=homologs[1], data=df, hue='N-glycan',
            fit_reg=False, scatter_kws={'s':22, 'alpha':0.75},
            palette={True:"#E69F00", False:"#56B4E9"})
    g.fig.get_children()[-1].set_bbox_to_anchor((0.95, 0.37, 0, 0))
    fig = plt.gcf()
    ax = plt.gca()
    seaborn.regplot(x=homologs[0], y=homologs[1], data=df, scatter=False, ax=ax,
            line_kws={'color':'gray', 'alpha':0.2})
    ax.set_aspect('equal')
    labelstr = '{\large $\omega_r < 1 \; \leftarrow \log_{10} Q \\rightarrow \; \omega_r > 1$}'
    plt.xlabel(labelstr + '\n' + homologs[0], fontsize=17)
    plt.ylabel(homologs[1] + '\n' + labelstr, fontsize=17)
    plt.tick_params(axis='both', which='major', labelsize=11, length=3, width=0.5, pad=2)
    (xmin, xmax) = plt.xlim()
    (ymin, ymax) = plt.ylim()
    plt.yticks([-10, 0, 10])
    plt.xticks([-10, 0, 10])
    plt.xlim(min([xmin, ymin]), max([xmax, ymax]))
    plt.ylim(min([xmin, ymin]), max([xmax, ymax]))
    plt.text(xmin + 0.03 * (xmax - xmin), ymax - 0.03 * (ymax - ymin), 
            'R = {0:.2f}'.format(scipy.stats.pearsonr(df[homologs[0]], df[homologs[1]])[0]),
            verticalalignment='top', horizontalalignment='left', fontsize=15)
    fig.set_size_inches(3.7, 3.7)
    fig.tight_layout()
    plt.savefig(divselcorr)
    plt.close()
showPDF(divselcorr, width=400)
```

#### Visualize phylogenetic tree¶

We draw an image of the phylogenetic tree, using the one inferred with the YNKGP M0 model.

In [31]:

```
treefile = os.path.join(phydmsdir, 'YNGKP_M0_tree.newick')
tree = Bio.Phylo.read(treefile, 'newick')
tree.root_at_midpoint()

treefigfile = os.path.join(phydmsdir, 'tree_plot.pdf')

if not os.path.isfile(treefigfile):
    Bio.Phylo.draw(tree,
            label_func=lambda x: str(x) if str(x) in homologs else '',
            label_colors=dict([(env, 'darkblue') for env in homologs]),
            do_show=False,
            )
    
    treefig = plt.gcf()
    treefig.set_size_inches(7, 11)
    ax = treefig.axes[0]
    ax.axis('off')

    # add scale bar
    (x0, x1, y0, y1) = plt.axis()
    xstart = x0 + 0.3 * (x1 - x0)
    barlen = 0.1
    yline = y0 - 0.05 * (y1 - y0)
    ytext = yline - 0.01 * (y1 - y0)
    ax.set_ylim(ytext, y1)
    plt.plot([xstart, xstart + barlen], [yline, yline], color='black', 
            linestyle='-', linewidth=2)
    plt.text(xstart + barlen / 2., ytext, 
            '{0:.1} codon substitutions / site'.format(barlen),
            horizontalalignment='center',
            verticalalignment='top',
            color='black',
            fontsize=17)

    treefig.tight_layout()
    treefig.savefig(treefigfile)
    
showPDF(treefigfile, width=300)
```

## Visualize amino-acid preferences¶

### Logo plots for entire Env¶

Now we use dms2\_logoplot to visualize the amino-acid preferences for each homolog in the form of logo plots.

These are the **average** preferences across replicates, **re-scaled** by the stringency parameter inferred in the phylogenetic analysis above.

We overlay the wildtype amino-acid at each site.

We also overlay the evidence that each site is under diversifying selection ($\omega\_r > 1$) or excess purifying selection ($\omega\_r < 1$) as inferred in the `phydms` analysis above.

We also overlay the breakdown of Env into different *regions* (gp120 variable loops, other parts of gp120, gp41), using the definitions (in HXB2 numbering) found in ./data/Env\_regions.csv.

In [32]:

```
# write stringency re-scaled average prefs to file
rescaled_prefsfiles = {}
for (prefsname, prefsfile) in prefsfiles.items():
    env = [env for env in homologs if env in prefsname]
    assert len(env) == 1
    beta = stringency[env[0]]
    rescaled_prefsfile = os.path.join(os.path.dirname(prefsfile), 
            'rescaled_' + os.path.basename(prefsfile))
    rescaled_prefsfiles[prefsname] = rescaled_prefsfile
    (dms_tools2.prefs.rescalePrefs(pandas.read_csv(prefsfile), beta)
            .to_csv(rescaled_prefsfile, index=False))
    
# create region overlay, removing missing sites, interpolating ones with letters
regionoverlay = {}
regions = pandas.read_csv('./data/Env_regions.csv').set_index('site')['R'].to_dict()
for env in homologs:
    regionoverlay[env] = os.path.join(prefsdir, env + '_regionoverlay.csv')
    overlay = []
    for r in pandas.read_csv(prefsfiles[env + '-avg'])['site']:
        if not r.isdigit():
            rnum = int(r[ : -1]) # remove trailing letter (e.g., '100a' -> '100')
        else:
            rnum = int(r)
        overlay.append((r, regions[rnum]))
    pandas.DataFrame(overlay, columns=['site', 'R']).to_csv(regionoverlay[env], index=False)

# create files for wildtype overlay
wildtypeoverlay = {}
for env in homologs:
    wildtypeoverlay[env] = os.path.join(prefsdir, env + '_wildtypeoverlay.csv')
    sites = pandas.read_csv(prefsfiles[env + '-avg'])['site'].values
    df = (pandas.read_csv(renumbfile[env])
            .rename(columns={'new':'site'})
            [['site', 'wildtype']]
            .query('site in @sites')
            .to_csv(wildtypeoverlay[env], index=False)
            )
    
# make logo plots
logofiles = {}
for env in homologs:
    print("\nLogo plot of rescaled average preferences for {0}".format(env))
    logofiles[env] = os.path.join(prefsdir, env + '_prefs.pdf')
    log = !dms2_logoplot \
            --prefs {rescaled_prefsfiles[env + '-avg']} \
            --name {env} \
            --outdir {prefsdir} \
            --nperline 84 \
            --numberevery 5 \
            --overlay1 {wildtypeoverlay[env]} wildtype wildtype \
            --overlay2 {omegabysitefiles[env]} omegabysite omegabysite \
            --overlay3 {regionoverlay[env]} R region \
            --overlaycolormap coolwarm
    showPDF(logofiles[env])
```

```
Logo plot of rescaled average preferences for BG505
```

```
Logo plot of rescaled average preferences for BF520
```

## Further analysis of diversifying / purifying selection¶

Now that we have made the logo plots, we do some further analysis of the diversifying and purifying selection.

### Logo plots of diversifying / purifying selection sites¶

We now use the `rplot` module defined as part of the dms\_tools2 Python API to plot the re-scaled preferences and the amino-acid frequencies in our clade A alignment for all sites where $\omega\_r$ is $>$ or $<$ 0 at a Q-value of $< 0.05$ for **both** homologs.
Note that `rplot` requires installation of rpy2.

In [33]:

```
# data frame of natural amino-acid freqs from alignment
aafreqs = dms_tools2.prefs.aafreqsFromAlignment(alignmentfile, codon_to_aa=True)
aafreqs['site'] = aafreqs['site'].astype('str') # pref sites are string

# read re-scaled average prefs into dataframes
pref_dfs = [pandas.read_csv(rescaled_prefsfiles[env + '-avg']).assign(stacklabel=env) 
        for env in homologs]

# now get shared sites among alignment and prefs
sharedsites = aafreqs['site']
for df in pref_dfs:
    sharedsites = numpy.intersect1d(sharedsites, df['site'])

# merge prefs and alignment freqs, keeping shared sites
logodata = pandas.concat(pref_dfs + [aafreqs.assign(stacklabel='alignment')])
logodata = logodata[logodata['site'].isin(sharedsites)]

# get sites significant for both homologs in same direction
sigsites = omegabysite.groupby('site').agg(['min', 'max'])
sigsites = sigsites[(sigsites['omega']['min'] > 1) == (sigsites['omega']['max'] > 1)]
sigsites = sigsites[sigsites['Q']['max'] < qthreshold]

selsiteplots = {}
ncol = 7
for (seltype, op) in [('diversifying', operator.gt), ('purifying', operator.lt)]:
    selsites = sigsites[op(sigsites['omega']['max'], 1)]['Q']['max'].sort_values()
    selsites.index = selsites.index.astype('str')
    facetlabels = dict([(site, 'site {0}\nQmax = {1:.2g}'.format(site, q))
            for (site, q) in selsites.iteritems()])
    print("\nPlotting the {0} sites under {1} selection in all homologs at Q < {2}"
            .format(len(selsites), seltype, qthreshold))
    selsiteplots[seltype] = os.path.join(prefsdir, '{0}_selsites.pdf'.format(seltype))
    dms_tools2.rplot.facetedGGSeqLogo(
            logodata=(logodata[logodata['site'].isin(selsites.index)]
                    .rename(columns={'site':'facetlabel'})
                    .replace({'facetlabel':facetlabels})
                    ),
            chars=AAS, 
            plotfile=selsiteplots[seltype], 
            width=1.8 * min(ncol, len(selsites)), 
            height=2.8 * math.ceil(len(selsites) / ncol), 
            ncol=ncol
            )
    showPDF(selsiteplots[seltype])
```

```
Plotting the 48 sites under diversifying selection in all homologs at Q < 0.05
```

```
Plotting the 26 sites under purifying selection in all homologs at Q < 0.05
```

In these plots, the diversifying selection logos are not particularly informative since the limited amount of evolutionary time means that the natural variation is still generally less than the variation in the preferences (perhaps this would look different with a much wider alignment).

However, for the purifying selection plot is quite informative -- we can see at all of these sites, the natural sequences tend to have a single conserved residue even though the preferences for both homologs suggest that a range of amino acids should be tolerated.

### Glycosylation sites¶

We plot the preferences and alignment frequencies for all N-linked glycans present in both BG505 and BF520.
From this plot, we can see that many of the N-linked glycans that are conserved among natural sequences are **not** required for the experiments used in our deep mutational scanning.

In [34]:

```
# get all three sites in N-glycans
glymotifsites = []
allsites = logodata.site.unique().tolist()
motif_d = {}
stack_d = {}
for r in nglycan[nglycan['N-glycan']].site:
    if r in allsites:
        assert isinstance(r, str)
        r2 = allsites[allsites.index(r) + 1]
        r3 = allsites[allsites.index(r) + 2]
        glymotifsites += [r, r2, r3]
        motif_d[r] = motif_d[r2] = motif_d[r3] = r
        stack_d[r] = 'N'
        stack_d[r2] = 'X'
        stack_d[r3] = 'S/T'
        
# get data to plot
glycandata = (logodata
              .query('site in @glymotifsites')
              .assign(motif=lambda x: [motif_d[r] for r in x.site])
              .assign(facetlabel=lambda x: x.motif + ', ' + x.stacklabel,
                      stacklabel=lambda x: [stack_d[r] for r in x.site],
                      motif=lambda x: x.motif.astype(int))
              .sort_values(['motif', 'facetlabel'])
              )

# make plot
glycanprefsplot = os.path.join(prefsdir, 'glycan_prefs.pdf')
ncol = 9
nstacks = len(glycandata) // 3
dms_tools2.rplot.facetedGGSeqLogo(
        logodata=glycandata,
        chars=AAS, 
        plotfile=glycanprefsplot, 
        width=1.5 * min(ncol, nstacks), 
        height=1.8 * math.ceil(nstacks / ncol), 
        ncol=ncol
        )
showPDF(glycanprefsplot)
```

### Solvent accessibility of sites of diversifying and purifying selection¶

We have used dssp to calculate the solvent accessibility of residues in the BG505 SOSIP trimer based on two crystal structures:

1. A hand-modified version of PDB structure 5FYL; the dssp results for this structure are in ./data/5FYL\_dssp.txt. This structure gives the "closed" pre-fusion conformation.
2. A hand-modified version of PDB structure 5VN3; the dssp results for this structure are in ./data/5VN3\_dssp.txt. This structure gives the CD4-bound conformation.

We calculate the relative-solvent accessibility (RSA) for each residue in this structure, and compare RSAs for all sites and the sites that are under diversifying and purifying selection in **both** homologs using a cutoff of Q < 0.05.
We categorize sites by whether they are an N-linked glycosylated Asn.
We then make box and swarm plots summarizing the results:

In [35]:

```
pdbs = ['5FYL', '5VN3']
pdb_description = {'5FYL':'closed pre-fusion (5FYL)',
                   '5VN3':"CD4-bound state (5VN3)"}

# chains for a gp120 and g41 monomer
pdb_chains = {'5FYL':['X', 'A'],
              '5VN3':['G', 'A']}

dsspfiles = dict([(pdb, './data/' + pdb + '_dssp.txt') for pdb in pdbs])

# make merged data frame with DSSP results 
full_dssp_df = pandas.concat([
               pandas.concat([dms_tools2.dssp.processDSSP(dsspfiles[pdb], chain=c)
                              for c in pdb_chains[pdb]])
                     .assign(pdb=pdb)
               for pdb in pdbs])
full_dssp_df['site'] = full_dssp_df['site'].astype('str')

# get sites for which omega determined, assign selection type and N-glycan status
dssp_df = full_dssp_df[full_dssp_df['site'].isin(omegabysite['site'])]
dssp_df['seltype'] = 'neither'
for (seltype, op) in [('diversifying', operator.gt), ('purifying', operator.lt)]:
    selsites = sigsites[op(sigsites['omega']['max'], 1)].index.astype('str')
    dssp_df.loc[dssp_df['site'].isin(selsites), 'seltype'] = seltype
dssp_df = dssp_df.merge(omegabysite, on='site')
print("Here are the number of sites with known RSA in each category:")
display(HTML(dssp_df.groupby(['seltype', 'Env', 'pdb', 'N-glycan']).size()
        .reset_index(name='nsites').to_html(index=False)))

# make plot
selsites_rsa_plot = os.path.join(phydmsdir, 'selsites_rsa.pdf')
(fig, axes) = plt.subplots(nrows=1, ncols=len(pdbs), sharey=True)
for (i, pdb) in enumerate(pdbs):
    seaborn.boxplot(x='seltype', y='RSA', data=dssp_df.query('pdb == @pdb'),
            color='gray', showfliers=False, ax=axes[i])
    seaborn.swarmplot(x='seltype', y='RSA', data=dssp_df.query('pdb == @pdb'),
            alpha=0.4, hue='N-glycan', palette={True:"#E69F00", False:"#56B4E9"},
            size=5, ax=axes[i])
    axes[i].set_xlabel('selection type ($Q < {0:.2f}$)'.format(qthreshold))
    if i == 0:
        axes[i].set_ylabel('relative solvent accessibility')
        axes[i].legend().set_visible(False)
    else:
        axes[i].set_ylabel('')
        axes[i].legend(title='N-glcyan', bbox_to_anchor=(1.05, 0.5), loc=2,
                       borderaxespad=0)
    axes[i].set_title(pdb_description[pdb])
fig.set_size_inches(9, 3.7)
fig.subplots_adjust(right=0.85, left=0.08, bottom=0.18)
fig.savefig(selsites_rsa_plot)
plt.close()
showPDF(selsites_rsa_plot, width=700)
```

```
Here are the number of sites with known RSA in each category:
```

| seltype | Env | pdb | N-glycan | nsites |
| --- | --- | --- | --- | --- |
| diversifying | BF520 | 5FYL | False | 45 |
| diversifying | BF520 | 5VN3 | False | 38 |
| diversifying | BG505 | 5FYL | False | 45 |
| diversifying | BG505 | 5VN3 | False | 38 |
| neither | BF520 | 5FYL | False | 464 |
| neither | BF520 | 5FYL | True | 14 |
| neither | BF520 | 5VN3 | False | 434 |
| neither | BF520 | 5VN3 | True | 12 |
| neither | BG505 | 5FYL | False | 464 |
| neither | BG505 | 5FYL | True | 14 |
| neither | BG505 | 5VN3 | False | 434 |
| neither | BG505 | 5VN3 | True | 12 |
| purifying | BF520 | 5FYL | False | 19 |
| purifying | BF520 | 5FYL | True | 3 |
| purifying | BF520 | 5VN3 | False | 19 |
| purifying | BF520 | 5VN3 | True | 3 |
| purifying | BG505 | 5FYL | False | 19 |
| purifying | BG505 | 5FYL | True | 3 |
| purifying | BG505 | 5VN3 | False | 19 |
| purifying | BG505 | 5VN3 | True | 3 |

## Compare preferences between homologs¶

We now compare the preferences between homologs using the `compareprefs` module of the dms\_tools2 Python API.

For these comparison, we first re-scale the preferences for each replicate by the stringency parameter inferred above by phydms using an ExpCM informed the amino-acid preferences for that homolog averaged across replicates.

### Comparison of the Env homologs to each other¶

#### Compute distances between preferences¶

First we compute the distances between the preferences of the two homologs at each site, using the *RMSDcorrected* metric to correct for noise of within-replicate measurements on the same homolog.
This approach is based on the strategy of Doud et al (2015).
Essentially it looks at the difference between replicates after subtracting off the noise within.
We use the implementation in the `dms_tools2.compareprefs.comparePrefs` function.

We read these distances into a dataframe, and also write them to a file.

In [36]:

```
prefsdistdir = os.path.join(resultsdir, 'prefsdist')
if not os.path.isdir(prefsdistdir):
    os.mkdir(prefsdistdir)

assert len(homologs) == 2, "plotting needs to be refactored if > 2 homologs"
prefs1 = [f for (name, f) in sorted(rescaled_prefsfiles.items()) if 
                re.search('^{0}\-\d+$'.format(homologs[0]), name)]
prefs2 = [f for (name, f) in sorted(rescaled_prefsfiles.items()) if 
                re.search('^{0}\-\d+$'.format(homologs[1]), name)]

prefsdist = dms_tools2.compareprefs.comparePrefs(prefs1, prefs2)
prefsdistfile = os.path.join(prefsdistdir, '{0}_to_{1}_prefs_dist.csv'.format(*homologs))
prefsdist.to_csv(prefsdistfile, index=False)
```

#### Plot example sites that illustrate the principle of the preferences comparison¶

We now plot the replicate data for sites with high and low distance (`RMSDcorrected`) to illustrate the principle of how these calculations work.
The sites plotted here are hand chosen to illustrate the range of data that are observed.
They show the preferences measured for each replicate, and then the difference between the replicate-average preferences for each homolog scaled to the total distance (*RMSDcorrected*):

In [37]:

```
examplesites = list(map(str, [598, 528, 288, 512]))

# print distance statistics for example sites
display(HTML(prefsdist.query('site in @examplesites')
        .drop(AAS, axis=1).to_html(index=False)))

# plot prefs for each replicate for each homolog for example sites
exampleprefs = (
        pandas.concat(
            [pandas.read_csv(f).assign(homolog=homologs[i], replicate=j + 1) for
            (i, iprefs) in enumerate([prefs1, prefs2]) for (j, f) in enumerate(iprefs)])
        .query('site in @examplesites')
        .assign(stacklabel=lambda x: x['replicate'],
                facetlabel=lambda x: x['homolog'] + '\nsite ' + x['site'].astype(str),
                homolog=lambda x: pandas.Categorical(x['homolog'], homologs),
                site=lambda x: pandas.Categorical(x['site'], examplesites))
        .sort_values(['site', 'homolog'])
        [['facetlabel', 'stacklabel'] + AAS]
        )
exampleprefsplot = os.path.join(prefsdistdir, 'exampleprefs.pdf')
dms_tools2.rplot.facetedGGSeqLogo(exampleprefs, AAS,
        exampleprefsplot, width=2.2, height=1.8 * len(examplesites), 
        ncol=2, xlabelsrotate=False)

# plot prefs dist for example sites
exampleprefsdist = (
        prefsdist
        .query('site in @examplesites')
        [['site'] + AAS]
        .assign(stacklabel='',
                facetlabel=lambda x: 'diff\n' + x['site'].astype(str),
                site=lambda x: pandas.Categorical(x['site'], examplesites))
        .sort_values('site')
        [['facetlabel', 'stacklabel'] + AAS]
        )
exampleprefsdistplot = os.path.join(prefsdistdir, 'exampleprefsdist.pdf')
dms_tools2.rplot.facetedGGSeqLogo(exampleprefsdist, AAS,
        exampleprefsdistplot, width=0.8, height=1.8 * len(examplesites),
        ncol=1)

showPDF([exampleprefsplot, exampleprefsdistplot], width=250)
```

| site | RMSDcorrected | RMSDbetween | RMSDwithin |
| --- | --- | --- | --- |
| 288 | 0.458152 | 0.727987 | 0.269835 |
| 512 | 0.520506 | 0.702348 | 0.181843 |
| 528 | 0.158997 | 0.351500 | 0.192503 |
| 598 | 0.012085 | 0.040193 | 0.028107 |

#### Collect information about wildtype amino acids¶

We want to do some comparisons specifically at sites that changed between the homologs.
We therefore add to our data frame of distances information on the wildtype amino acid at each site in each homolog:

In [38]:

```
# add a column holding the wildtype amino acid for each homolog
for env in homologs:
    df = (pandas.read_csv(renumbfile[env])
            .rename(columns={'new':'site', 'wildtype':env})
            [['site', env]]
            )
    prefsdist = prefsdist.merge(df)

# indicate whether wildtype residue is variable or conserved
prefsdist['conservation'] = prefsdist.apply(lambda x:
        'conserved' if x[homologs[0]] == x[homologs[1]] else 'variable', axis=1)
```

#### Logo plot of scaled differences between homologs at each site¶

Now we use dms2\_logoplot to plot the difference between homologs at each site.
The plots are scaled so that the height of the letter stack in each direction is proportional to *RMSDcorrected*, and the height of each letter is proportional to the difference in the replicate-average preference measurements for that homolog.

We include an overlay that indicates whether that residue differs between BG505 and BF520.

In [39]:

```
prefsdistlogoname = '{0}-to-{1}'.format(*homologs)
prefsdistlogo = os.path.join(prefsdistdir, prefsdistlogoname + '_diffprefs.pdf')
conservationoverlay = os.path.join(prefsdistdir, 'conservation.csv')
(prefsdist[['site', 'conservation']]
          .rename(columns={'conservation':'V/C'})
          .to_csv(conservationoverlay, index=False)
          )
log = !dms2_logoplot \
        --diffprefs {prefsdistfile} \
        --outdir {prefsdistdir} \
        --name {prefsdistlogoname} \
        --nperline 84 \
        --numberevery 5 \
        --overlay1 {conservationoverlay} V/C variable/conserved \
        --overlaycolormap binary \
        --ignore_extracols yes
        
showPDF(prefsdistlogo)
```

### Analyze distribution of preference differences¶

We compare the observed preference differences for the Env homologs to various other relevant distributions (e.g., a randomization-generated null and non-homologous proteins).

#### Compute distributions of preference differences¶

We compute the distribution of *RMSDcorrected* values for all sites shared between the Env homologs, and compare this distribution to several things:

- The distribution generated by randomly assigning 3 replicates per homolog for all such assignments. This gives the expected distribution of distances if there is no true signal, and everything is experimental noise.
- The distribution of differences between the 3 replicates of each Env homolog and the three replicates of H1 influenza HA as measured by Doud and Bloom (2016). Since HA and Env are not homologous (at least not in a plausibly alignable fashion), the sites are just put in a 1:1 correspondance based on sequence numbering. This gives an idea of what is expected if the preferences are "maximally diverged" and everything has shifted. The preferences for HA are in the following files:

  - data/Doud2016\_HA\_replicate-1\_prefs.csv
  - data/Doud2016\_HA\_replicate-2\_prefs.csv
  - data/Doud2016\_HA\_replicate-3\_prefs.csv

  These preferences are **not** yet re-scaled, but as described in the Doud2016 dms\_tools2 example, they should be re-scaled by a stringency parameter of $\beta = 2.05$.

In [40]:

```
# the actual distances between the Env homologs
actual_dists = prefsdist['RMSDcorrected'].tolist()

# distances for all randomizations of 3 replicates per homolog
rand_dists = []
assert len(prefs1) == len(prefs2) == 3, "code expects 3 replicates / homologs"
for set1 in itertools.combinations(prefs1 + prefs2, 3):
    set2 = [iprefs for iprefs in prefs1 + prefs2 if iprefs not in set1]
    rand_dists += (dms_tools2.compareprefs.comparePrefs(set1, set2)
                      ['RMSDcorrected'].tolist())
    
# get re-scaled HA prefs
ha_stringency = 2.05
ha_prefs = []
for r in [1, 2, 3]:
    unscaled = './data/Doud2016_HA_replicate-{0}_prefs.csv'.format(r)
    rescaled = os.path.join(prefsdistdir, 'HA_rescaled_{0}.csv'.format(r))
    (dms_tools2.prefs.rescalePrefs(pandas.read_csv(unscaled), ha_stringency)
            .to_csv(rescaled, index=False))
    ha_prefs.append(rescaled)

# distances from each homolog to HA prefs
ha_dist = {}
for (homolog, homologprefs) in zip(homologs, [prefs1, prefs2]):
    ha_dist[homolog] = (dms_tools2.compareprefs.comparePrefs(ha_prefs, homologprefs)
                        ['RMSDcorrected'].tolist())
```

#### Estimate significant differences¶

Now we want to estimate a significance threshold for when distances (*RMSDcorrected*) are significant for our actual Env homologs.
We define a *P*-value as the fraction of the replicate-randomized distribution that is $\ge$ each distance observed for the real homolog-homolog comparison.
We then using the Benjamini-Hochberg procedure with an FDR of 0.1 to find sites where the observed difference is greater than that in the null.

In [41]:

```
# compute P-values
rand_dists = numpy.array(rand_dists)
actual_dists = sorted(actual_dists, reverse=True)
n = float(len(rand_dists))
pvals = [numpy.greater_equal(rand_dists, d).sum() / n for d in actual_dists]
assert pvals == sorted(pvals), "pvals should be sorted if actual_dists sorted"

# get minimum distance at which we reject null at FDR of 0.1
fdr = 0.1
reject = statsmodels.stats.multitest.multipletests(
          pvals, alpha=fdr, method='fdr_bh')[0].tolist()
assert any(reject), "no significants sites at FDR, rest of code won't work"
min_dist = actual_dists[reject.index(False) - 1]
print("At FDR of {0}, we consider homolog-to-homolog diffs significant if they "
      "exceed {1:.3f}. There are {2} such sites out of {3} total.".format(
      fdr, min_dist, sum(reject), len(actual_dists)))
```

```
At FDR of 0.1, we consider homolog-to-homolog diffs significant if they exceed 0.221. There are 30 such sites out of 659 total.
```

#### Plot distributions of shifts¶

Now we use joypy to make joy plots showing the distributions of shifts in preferences as quantified by the *RMSDcorrected* values.

In [42]:

```
distplot = os.path.join(prefsdistdir, 'distance_distribution.pdf')

if not os.path.isfile(distplot):
    # the data to plot
    joyplot_data = collections.OrderedDict([
                ('{0} vs HA'.format(homologs[0]), ha_dist[homologs[0]]),
                ('{0} vs HA'.format(homologs[1]), ha_dist[homologs[1]]),
                ('randomized\n{0} vs {1}'.format(*homologs), rand_dists),
                ('{0} vs {1}'.format(*homologs), actual_dists * 20),
                ])
    # colors for each comparison: same color for the two HA comparisons
    cmap = matplotlib.colors.ListedColormap(["#009E73", "#009E73", "#56B4E9", "#E69F00"])

    # make the joyplot
    joypy.joyplot(joyplot_data,
              grid='x',
              overlap=1,
              linewidth=1.5,
              figsize=(6, 4.8),
              colormap = cmap,
             )
    plt.xlabel('shift in preferences ($RMSD_{corrected}$)', size=13)

    # draw line indicating sites "significant" at FDR defined above
    (ymin, ymax) = (0.06, 0.24)
    plt.axvline(x=min_dist, ymin=ymin, ymax=ymax, ls='dotted', lw=1.5, color='black')
    plt.text(min_dist + 0.02, ymin + (ymax - ymin) / 2, 
            'shifts $\ge {0:.2f}$ significant\nat FDR of {1:.1f}'.format(min_dist, fdr),
            horizontalalignment='left', verticalalignment='center', size=13)
    xticks = [-0.2, 0, 0.2, 0.4, 0.6, 0.8, 1.0]
    plt.xticks(xticks, ['{0:.1f}'.format(x) for x in xticks])

    plt.tight_layout()
    plt.savefig(distplot)
    plt.close()
    
showPDF(distplot, width=600)
```

#### Plot preferences for sites with significant shifts¶

For all sites with significant shifts, we plot the replicate-average preferences for each homolog.

In [43]:

```
shiftlogodata = (pandas.concat(pref_dfs)
                 .assign(site=lambda x: x.site.astype(str))
                 .merge(prefsdist[['site', 'RMSDcorrected'] + homologs])
                 .sort_values(['RMSDcorrected', 'stacklabel'], ascending=False)
                 .query('RMSDcorrected >= @min_dist')
                 .assign(facetlabel=lambda x: 'site ' + x['site'] + '\nshift ' + 
                         x['RMSDcorrected'].map('{0:.2f}'.format) + '\n' +
                         x[homologs[0]] + '     ' + x[homologs[1]])
                 )

nsites = len(shiftlogodata['site'].unique())
print("Plotting the {0} sites with shifts greater than {1:.2f}".format(nsites, min_dist))

shiftedsitesplot = os.path.join(prefsdistdir, 'shifted_sites.pdf')
ncol=15
dms_tools2.rplot.facetedGGSeqLogo(
        logodata=shiftlogodata,
        chars=AAS, 
        plotfile=shiftedsitesplot, 
        width=1.1 * min(ncol, nsites), 
        height=2.6 * math.ceil(nsites / ncol), 
        ncol=ncol
        )
showPDF(shiftedsitesplot)
```

```
Plotting the 30 sites with shifts greater than 0.22
```

### Analyze features of sites with shifted preferences¶

Now we look at the properties of sites that have shifted in preferences.
For instance, are these sites disproportionately likely to be ones that have substituted during the divergence of the Env homologs?
Do they have certain structural features?

#### Aggregate information on shifts and conservation¶

First we create a dataframe that gives the magnitude of the shift (*RMSDcorrected*), whether this shift is *significant* by the criterion above, and whether the site substituted between the homologs.

In [44]:

```
shifts = (prefsdist.drop(AAS + ['conservation'], axis=1)
          .assign(significant_shift=lambda x: x.RMSDcorrected >= min_dist,
                  substituted=lambda x: x[homologs[0]] != x[homologs[1]])
          )
```

#### Get structural information (distance matrix)¶

One of the things that will examine is the structural properties of shifted sites.
So we add key structural information to our data frame storing information on the shifts.

We also want a distance matrix giving the distance between all pairs of residues.
Again, we do this using two structures and the following PDBs:

1. The hand-modified version of PDB structure 5FYL found in ./data/5FYL\_Env\_trimer\_rmTER.pdb. Exactly how this file was created is described in the analysis README file. Essentially, it shows the full "closed" pre-fusion trimer with the gp120 chains labeled as *X*, *Y*, and *Z*, and the gp41 chains labeled as *A*, *B*, and *C*.
2. The hand modified version of PDB structure 5VN3 found in ./data/5VN3\_Env\_trimer.pdb. Exactly how this file was obtained is described in the analysis README file. Essentially, it shows the CD4-bound trimer with the gp120 chains labeled as *G*, *I*, and *J*, and the gp41 chains labeled as *A*, *B*, and *D*.

We compute the distances using the `protstruct.distMatrix` function that is part of the dms\_tools2 Python API.
We compute the distances between the closest atoms in each residue, not just computing inter-monomer distances but rather the closest distance to any equivalent chain in the structure:

In [45]:

```
pdbfiles = {'5FYL':'./data/5FYL_Env_trimer_rmTER.pdb',
            '5VN3':'./data/5VN3_Env_trimer.pdb'}
equivchains = {'5FYL':{'A':['B', 'C'], 'X':['Y', 'Z']},
               '5VN3':{'A':['B', 'D'], 'G':['I', 'J']}}

dist_matrix = {}
dist_residues = {}
for pdb in pdbs:
    # read dist matrix from pickle file if possible as it takes time to compute
    dist_matrix_pickle = os.path.join(prefsdistdir, pdb + '_dist_matrix.pickle')
    if os.path.isfile(dist_matrix_pickle) and use_existing == 'yes':
        with open(dist_matrix_pickle, 'rb') as f:
            (dist_residues[pdb], dist_matrix[pdb]) = pickle.load(f)
    else:
        (dist_residues[pdb], dist_matrix[pdb]) = dms_tools2.protstruct.distMatrix(
            pdbfile=pdbfiles[pdb],
            chains=pdb_chains[pdb],
            dist_type='any',
            equivchains=equivchains[pdb])
        with open(dist_matrix_pickle, 'wb') as f:
            pickle.dump((dist_residues[pdb], dist_matrix[pdb]), f)
        
    # ignore contacts of residues with themselves
    numpy.fill_diagonal(dist_matrix[pdb], numpy.nan)
        
    print("Computed intra-residue distances for {0} sites in {1}.".format(
            pdb, len(dist_residues[pdb])))
```

```
Computed intra-residue distances for 5FYL sites in 579.
Computed intra-residue distances for 5VN3 sites in 525.
```

Now we add columns to our dataframe on the shifts that gives the closest distance of each residue to some **other** residue that has substituted between homologs, and the closest distance of each residue to some **other** residue with a significant shift.
We also create a columns variable specifying whether the distance is short enough to represent a "contact", defining a contact as any heavy atoms with 3.5 angstroms.

In [46]:

```
dist_cutoff = 3.5

# name for minimum of PDBs
pdb_min = 'min_{0}'.format('_'.join(pdbs))
pdbs_with_min = pdbs + [pdb_min]
pdb_description[pdb_min] = 'min distance {0}'.format(', '.join(pdbs))
dist_residues[pdb_min] = set.union(*map(set, dist_residues.values()))

for siteprop in ['substituted', 'significant_shift']:
    dist_df = []
    distname = '{0}_mindist'.format(siteprop)
    selsites = shifts[shifts[siteprop]].site
    for pdb in pdbs:
        indices = [j for (j, res) in enumerate(dist_residues[pdb]) if res in selsites.values]
        mindist = numpy.nanmin(dist_matrix[pdb][:, indices], 1)
        dist_df.append(pandas.DataFrame({
                'site':dist_residues[pdb],
                distname:mindist,
                'pdb':[pdb] * len(dist_residues[pdb])
                }))
    dist_df = pandas.concat(dist_df)
    
    # get minimum for the PDBs
    dist_df = pandas.concat([dist_df,
                             dist_df.groupby('site')
                                [distname]
                                .min()
                                .to_frame()
                                .reset_index()
                                .assign(pdb=pdb_min)
                            ])
        
    shifts = shifts.merge(dist_df, how='left')
    contactname = '{0}_contact'.format(siteprop)
    shifts[contactname] = shifts[distname] < dist_cutoff
    
    for pdb in pdbs_with_min:
        print('For {0}, {1} of {2} sites within {3} angstroms of a {4} site'.format(
            pdb, shifts.query('pdb == @pdb')[contactname].sum(), 
            len(shifts.site.unique()),
            dist_cutoff, siteprop))
```

```
For 5FYL, 260 of 659 sites within 3.5 angstroms of a substituted site
For 5VN3, 186 of 659 sites within 3.5 angstroms of a substituted site
For min_5FYL_5VN3, 281 of 659 sites within 3.5 angstroms of a substituted site
For 5FYL, 126 of 659 sites within 3.5 angstroms of a significant_shift site
For 5VN3, 110 of 659 sites within 3.5 angstroms of a significant_shift site
For min_5FYL_5VN3, 150 of 659 sites within 3.5 angstroms of a significant_shift site
```

#### Shifts and residue / residue-neighbor conservation¶

We now examine the shifts for three groups of residues:

1. Residues that have substituted between the two homologs
2. Residues that have not substituted but contact residues that have substituted
3. All other residues.

The goal is to see whether shifts are more frequency at substituted residues or residues that contact substituted residues.
As shown by the plot below, there is **not** a significant trend for greater shifts at sites of substitutions, or sites that contact substitutions.

In [47]:

```
conservation_shifts_plot = os.path.join(prefsdistdir, 'conservation_vs_shifts.pdf')

(fig, axes) = plt.subplots(nrows=1, ncols=len(pdbs_with_min), sharey=True)

ymax = shifts.RMSDcorrected.max()

for (i, pdb) in enumerate(pdbs_with_min):
    # categorize sites by conservation
    pdb_dist_residues = dist_residues[pdb]
    df = (shifts.query('pdb == @pdb')
          .assign(conservation=lambda x: 
                numpy.where(x.substituted, 'substitution', 
                numpy.where(x.substituted_contact, 'contacts\nsubstitution',
                'not near\nsubstitution')))
          .query('site in @pdb_dist_residues')
          )
    
    # indicate number of sites in each category, make categorical to plot in order
    col_order = ['substitution', 'contacts\nsubstitution', 'not near\nsubstitution']
    nsites_d = dict([(tup[0], '{0}\n({1} sites)'.format(*tup)) for tup 
                in df.groupby('conservation').count()['site'].items()])
    cats = [nsites_d[col] for col in col_order]
    df['conservation'] = pandas.Categorical(df['conservation'].replace(nsites_d),
                categories=cats)

    # make plot
    seaborn.boxplot(
            x='conservation', 
            y='RMSDcorrected',
            data=df, 
            showfliers=True,
            color='gray',
            ax=axes[i],
            )
    if i == 0:
        axes[i].set_ylabel('shift in preferences')
    else:
        axes[i].set_ylabel('')
    axes[i].set_xlabel('conserved in homologs?')
    axes[i].set_title(pdb_description[pdb])

    # add P values
    cat2 = cats[-1] # compare to last category
    x2 = cats.index(cat2) 
    yextend = 0.05
    for (x1, cat1) in enumerate(cats[ : -1]): # compare other categories to last one
        p = scipy.stats.mannwhitneyu(
                df[df.conservation == cat1].RMSDcorrected, 
                df[df.conservation == cat2].RMSDcorrected
                )[1]
        y = ymax * (1 + yextend * (4 * x1 + 1))
        axes[i].plot([x1, x1, x2, x2], [y, (1 + yextend) * y, (1 + yextend) * y, y],
                c='black', lw=1)
        axes[i].text((x1 + x2) / 2, y * (1 + yextend), '$P = {0:.2f}$'.format(p),
                ha='center', va='bottom', size=12)
    axes[i].set_ylim(top=y * (1 + yextend * 3))

# save plot
fig.set_size_inches(9.5, 3.9)
fig.tight_layout()
fig.savefig(conservation_shifts_plot)
plt.close()
showPDF(conservation_shifts_plot, width=700)
```

However, there is a borderline significant trend for the sites of significant shifts to have been ones that underwent substitutions, as shown by the table immediately below:

In [48]:

```
shifts_vs_subs_table = os.path.join(prefsdistdir, 'shifts_vs_subs_table.csv')

# make dataframe that just has site specific 
shifts_justsites = shifts[['site', 'significant_shift', 'substituted']].drop_duplicates()

tab = pandas.crosstab(shifts_justsites.significant_shift, 
                      shifts_justsites.substituted)
display(HTML(tab.to_html()))
p = scipy.stats.fisher_exact(tab)[1]
print("Fisher's exact test P-value: {0:.3f}".format(p))

tab.to_csv(shifts_vs_subs_table)
```

| substituted | False | True |
| --- | --- | --- |
| significant\_shift |  |  |
| False | 545 | 84 |
| True | 22 | 8 |

```
Fisher's exact test P-value: 0.055
```

#### Shifts and residue / residue neighbor conservation, take two¶

The analysis above considers *substituted* sites as a different category than sites that do and do not contact substitutions.
An alternative way to do the analysis would be to just group sites by whether they contact a substitutions **regardless** of whether or not they have substituted.

That analysis is done below.
We see that the results are unchanged -- sites that contact substitutions are no more likely to shift.

In [49]:

```
conservation_shifts_plot_2 = os.path.join(prefsdistdir, 'conservation_vs_shifts_2.pdf')

(fig, axes) = plt.subplots(nrows=1, ncols=len(pdbs_with_min), sharey=True)

for (i, pdb) in enumerate(pdbs_with_min):
    # categorize sites by conservation
    pdb_dist_residues = dist_residues[pdb]
    df = (shifts.query('pdb == @pdb')
                .assign(conservation=lambda x: 
                    numpy.where(x.substituted_contact, 'contacts\nsubstitution',
                    'not near\nsubstitution'))
                .query('site in @pdb_dist_residues')
                )

    # indicate number of sites in each category and make categorical to plot in order
    col_order = ['contacts\nsubstitution', 'not near\nsubstitution']
    nsites_d = dict([(tup[0], '{0}\n({1} sites)'.format(*tup)) for tup 
                in df.groupby('conservation').count()['site'].items()])
    cats = [nsites_d[col] for col in col_order]
    df['conservation'] = pandas.Categorical(df['conservation'].replace(nsites_d),
                categories=cats)

    # make plot

    seaborn.boxplot(
        x='conservation', 
        y='RMSDcorrected',
        data=df, 
        showfliers=True,
        color='gray',
        ax=axes[i],
        )
    if i == 0:
        axes[i].set_ylabel('shift in preferences')
    else:
        axes[i].set_ylabel('')
    axes[i].set_xlabel('conserved in homologs?')
    axes[i].set_title(pdb_description[pdb])

    # add P values
    cat2 = cats[-1] # compare to last category
    x2 = cats.index(cat2) 
    yextend = 0.05
    for (x1, cat1) in enumerate(cats[ : -1]): # compare other categories to last one
        p = scipy.stats.mannwhitneyu(
                df[df.conservation == cat1].RMSDcorrected, 
                df[df.conservation == cat2].RMSDcorrected
                )[1]
        y = ymax * (1 + yextend * (4 * x1 + 1))
        axes[i].plot([x1, x1, x2, x2], [y, (1 + yextend) * y, (1 + yextend) * y, y],
                c='black', lw=1)
        axes[i].text((x1 + x2) / 2, y * (1 + yextend), '$P = {0:.2f}$'.format(p),
                ha='center', va='bottom', size=12)
    axes[i].set_ylim(top=y * (1 + yextend * 3))

# save plot
fig.set_size_inches(8.4, 3.9)
fig.tight_layout()
fig.savefig(conservation_shifts_plot_2)
plt.close()
showPDF(conservation_shifts_plot_2, width=700)
```

#### Shifts and structural location¶

Next we look to see whether the sites of significant shifts tend to be surface-exposed or buried.

We have already read in the dssp-computed relative solvent accessibility (RSA) of all sites earlier in this notebook, so we just extract that information and add it to a dataframe that is keeping track of information on the shifts.

We first do this by plotting their distributions of relative solvent accessibilities (RSAs).
Shifted sites tend to buried more than exposed, but this trend is **not** significant (as evaluated by a Mann-Whitney test.

In [50]:

```
shifts_rsa = (
        prefsdist.drop(AAS + ['conservation'], axis=1)
                 .assign(significant_shift=lambda x: x.RMSDcorrected >= min_dist,
                         substituted=lambda x: x[homologs[0]] != x[homologs[1]])
        )

rsa = full_dssp_df[['site', 'pdb', 'RSA']].drop_duplicates()
print("We have RSA values for this many sites:")
print(rsa.groupby('pdb').count()[['site']])

shifts_rsa = shifts_rsa.merge(rsa, how='left')

rsa_shifts_plot = os.path.join(prefsdistdir, 'rsa_vs_shifts.pdf')

(fig, axes) = plt.subplots(nrows=1, ncols=len(pdbs), sharey=True)

ymax = shifts_rsa.RSA.max()

for (i, pdb) in enumerate(pdbs):
    # categorize sites by shifts
    pdb_dist_residues = dist_residues[pdb]
    df = (shifts_rsa
            .query('pdb == @pdb')
            .assign(shifted=lambda x: numpy.where(x.significant_shift, 'shifted', 
                    'not shifted'))
            .query('site in @pdb_dist_residues')
            )
    
    # indicate number of sites in each category and make categorical to plot in order
    col_order = ['shifted', 'not shifted']
    nsites_d = dict([(tup[0], '{0}\n({1} sites)'.format(*tup)) for tup 
                in df.groupby('shifted').count()['site'].items()])
    df['shifted'] = pandas.Categorical(df['shifted'].replace(nsites_d),
                         categories=[nsites_d[col] for col in col_order])

    # make plot
    seaborn.boxplot(
            x='shifted', 
            y='RSA',
            data=df, 
            showfliers=True,
            color='gray',
            ax=axes[i]
            )
    axes[i].set_xlabel('significantly shifted?')
    axes[i].set_title(pdb_description[pdb])
    if i == 0:
        axes[i].set_ylabel('relative solvent accessibility')
    else:
        axes[i].set_ylabel('')

    # add P values 
    p = scipy.stats.mannwhitneyu(
            df.query('significant_shift').RSA, 
            df.query('not significant_shift').RSA
            )[1]
    (x1, x2) = (0, 1)
    yextend = 1.05
    y = ymax * yextend
    axes[i].plot([x1, x1, x2, x2], [y, yextend * y, yextend * y, y], c='black', lw=1)
    axes[i].text((x1 + x2) / 2, y * yextend, '$P = {0:.2f}$'.format(p),
            ha='center', va='bottom', size=12)
    axes[i].set_ylim(top=y*(yextend**3))

# show and save plot
fig.set_size_inches(5.7, 3.9)
fig.tight_layout()
fig.savefig(rsa_shifts_plot)
plt.close()
showPDF(rsa_shifts_plot, width=600)
```

```
We have RSA values for this many sites:
      site
pdb       
5FYL   579
5VN3   525
```

We now continue the analysis by plotting the nearest distance of shifted sites to other shifted sites.

In [51]:

```
shifts_proximity_plot = os.path.join(prefsdistdir, 'shifts_proximity.pdf')

(fig, axes) = plt.subplots(nrows=1, ncols=len(pdbs_with_min), sharey=True)

ymax = shifts.significant_shift_mindist.max()

for (i, pdb) in enumerate(pdbs_with_min):
    pdb_dist_residues = dist_residues[pdb]
    df = (shifts
            .query('pdb == @pdb')
            .assign(shifted=lambda x: numpy.where(x.significant_shift, 'shifted', 
                    'not shifted'))
            .query('site in @pdb_dist_residues')
            )
    
    # indicate number of sites in each category and make categorical to plot in order
    col_order = ['shifted', 'not shifted']
    nsites_d = dict([(tup[0], '{0}\n({1} sites)'.format(*tup)) for tup 
                in df.groupby('shifted').count()['site'].items()])
    df['shifted'] = pandas.Categorical(df['shifted'].replace(nsites_d),
                         categories=[nsites_d[col] for col in col_order])
    
    # make plot
    seaborn.boxplot(
            x='shifted', 
            y='significant_shift_mindist',
            data=df, 
            showfliers=True,
            color='gray',
            ax = axes[i]
            )
    axes[i].set_ylim(bottom=0)
    axes[i].set_xlabel('significantly shifted?')
    axes[i].set_title(pdb_description[pdb])
    if i == 0:
        axes[i].set_ylabel('closest shifted site (\mbox{\\normalfont\AA})')
    else:
        axes[i].set_ylabel('')

    # add P values
    p = scipy.stats.mannwhitneyu(
            df.query('significant_shift').significant_shift_mindist, 
            df.query('not significant_shift').significant_shift_mindist
            )[1]
    (x1, x2) = (0, 1)
    yextend = 1.05
    y = ymax * yextend
    axes[i].plot([x1, x1, x2, x2], [y, yextend * y, yextend * y, y], c='black', lw=1)
    axes[i].text((x1 + x2) / 2, y * yextend, '$P = {0:.1g}$'.format(p),
            ha='center', va='bottom', size=12)
    axes[i].set_ylim(top=y*(yextend**3))

# show / save plot
fig.set_size_inches(8.2, 3.9)
fig.tight_layout()
fig.savefig(shifts_proximity_plot)
plt.close()
showPDF(shifts_proximity_plot, width=700)
```

### Examine "entrenchment"¶

One idea that has been studied recently (see Shah (2015) and Starr (2017)) is that differences that accumulate among homologs become "entrenched", and are more unfavorable to revert than they were to make in the first place.

We can test whether entrenchment is occurring by examining the average effect of mutating every site that differs between BG505 to BF520 to the identity in the other homolog.
If there is entrenchment, in generally these mutations should be unfavorable because each homolog will tend to have the residue that is more preferred at the site.

We quantify the mutational effects as the log base2 of ratio of the mutant to wildtype preference for the replicate-average values for each homolog (see dms\_tools2.prefs.prefsToMutEffects).
Negative values indicate unfavorable mutations, and positive ones indicate favorable mutations.
We then use joypy to make joy plots showing the distribution of mutational effects for mutations that differ between homologs, and for all mutations to each homolog.

From the joy plot below, we can see that there is a bit of entrenchment: the effect of mutation each homolog to the identity in the other is more often deleterious than not.
However, the magnitude of this entrenchment is still much smaller than the typical effect of a mutation, as the distribution of effects of these homolog-to-homolog mutations is much closer to being centered around zero than the distribution of the effects of *all* mutations to each homolog.

In [52]:

```
# get data for joy plot of distribution of mutational effects
joyplot_data = collections.OrderedDict()

for (env1, env2) in itertools.permutations(homologs, 2):
    
    # mutation effects in first homolog
    muts = (dms_tools2.prefs.prefsToMutEffects(
                pandas.read_csv(rescaled_prefsfiles[env1 + '-avg']), AAS)
            .assign(ismut=lambda x: x.initial != x.final) # is it a mutation?
            .query('ismut') # only keep mutations
            .merge(shifts[['site', 'substituted'] + homologs].drop_duplicates()) 
            .assign(from_wt=lambda x: x[env1] == x.initial) # is from wildtype
            .query('from_wt') # only keep mutations from wildtype
            .assign(homologmut=lambda x: (x[env2] == x.final) & x.substituted) # diff in homologs
            )
    
    print("Found mutational effects for {0} mutations to {1}, {2} of which are "
          "diffs with {3}".format(len(muts), env1, len(muts.query('homologmut')), env2))
    
    joyplot_data['{0}\nall mutations'.format(env1)] = muts.log2effect
    joyplot_data['{0}$\\rightarrow${1}'.format(env1, env2)] = muts.query('homologmut').log2effect

# name of the joy plot
entrenchmentplot = os.path.join(prefsdistdir, 'entrenchment.pdf')

# colors: one for differences, another for all mutations
cmap = matplotlib.colors.ListedColormap(["#56B4E9", "#E69F00"] * len(homologs))

# get xmin / xmax as extent of between-homolog mutations, or 1% / 99% of all mutations
xmin = min(map(lambda tup: tup[1].quantile(0.01) if 'all' in tup[0] else tup[1].min(),
        joyplot_data.items()))
xmax = max(map(lambda tup: tup[1].quantile(0.99) if 'all' in tup[0] else tup[1].max(),
        joyplot_data.items()))
xmin -= 0.15 * (xmax - xmin)
xmax += 0.15 * (xmax - xmin)

if not os.path.isfile(entrenchmentplot):
    # make the joyplot
    joypy.joyplot(joyplot_data,
              grid='x',
              overlap=0.6,
              linewidth=1.5,
              figsize=(6, 4.8),
              colormap=cmap,
              x_range=(xmin, xmax)
             )
    plt.xlabel('mutational effect', size=13)

    # only label integer xticks
    plt.xticks(plt.xticks()[0], 
            map(lambda x: '{0}'.format(int(x)) if x.is_integer() else '',  plt.xticks()[0]))

    # draw line at zero (neutral mutation)
    plt.axvline(x=0, ymin=0, ymax=1, ls='dotted', lw=1.5, color='black')

    # save and show
    plt.tight_layout()
    plt.savefig(entrenchmentplot)
    
showPDF(entrenchmentplot, width=600)
```

```
Found mutational effects for 12521 mutations to BG505, 92 of which are diffs with BF520
Found mutational effects for 12521 mutations to BF520, 92 of which are diffs with BG505
```

## Copy files to paper figures directory¶

We copy files that will be directly included in the paper into the figures subdirectory.

In [ ]:

```
import shutil

figsdir = '../paper/figures/'

# copy files that do not need name changes
for f in [treefigfile, identityplot, alignmentfile, maskfile, modelcomparefile, 
                gammamodelcomparefile, divselcorr, allprefscorr, glycanprefsplot,
                selsiteplots['purifying'], selsites_rsa_plot, allprefscorrcontour,
                exampleprefsplot, exampleprefsdistplot, distplot, shiftedsitesplot,
                conservation_shifts_plot, shifts_proximity_plot, rsa_shifts_plot,
                shifts_vs_subs_table, entrenchmentplot] + \
        prefcorrs + list(logofiles.values()) + \
        [rescaled_prefsfiles[env + '-avg'] for env in homologs] + \
        list(renumbfile.values()) + list(omegabysitefiles.values()):
    shutil.copy(f, figsdir)
    
# copy files that need name changes
for (f, newf) in [(countsplotprefix[env] + 'codonmuttypes.pdf', env + '_avgmutfreqs.pdf') 
                    for env in homologs]:
    shutil.copy(f, os.path.join(figsdir, newf))
    
# write data frames
corr_mutfreqs.to_csv(os.path.join(figsdir, 'avgmutfreqs.csv'), index=False)
omegabysite.to_csv(os.path.join(figsdir, 'merged_omegabysite.csv'), index=False)
shifts.to_csv(os.path.join(figsdir, 'BG505_to_BF520_prefs_dist.csv'), index=False)

# write prefs files
!zip {os.path.join(figsdir, 'all_prefs_unscaled.zip')} {' '.join(prefsfiles.values())}
```
