# Supplementary material for: Mapping mutational effects along the evolutionary landscape of HIV envelope
Source: eLife. 2018 Mar 28;7:e34420. doi: 10.7554/eLife.34420 (PMC5910023; doi:10.7554/eLife.34420)
Supplement: Supplementary file 1. — Specifically, this file contains a Jupyter notebook that performs the analysis, all required input data, and all reasonably sized output files. The Jupyter notebook downloads the deep sequencing data, processes it with the dms_tools2 software (Bloom, 2015, https://jbloomlab.github.io/dms_tools2/), and also performs a variety of downstream analyses that generate most of the figures for this paper. [file elife-34420-supp1.zip › analysis_code/results/phydms_analysis/selsites_rsa.pdf]

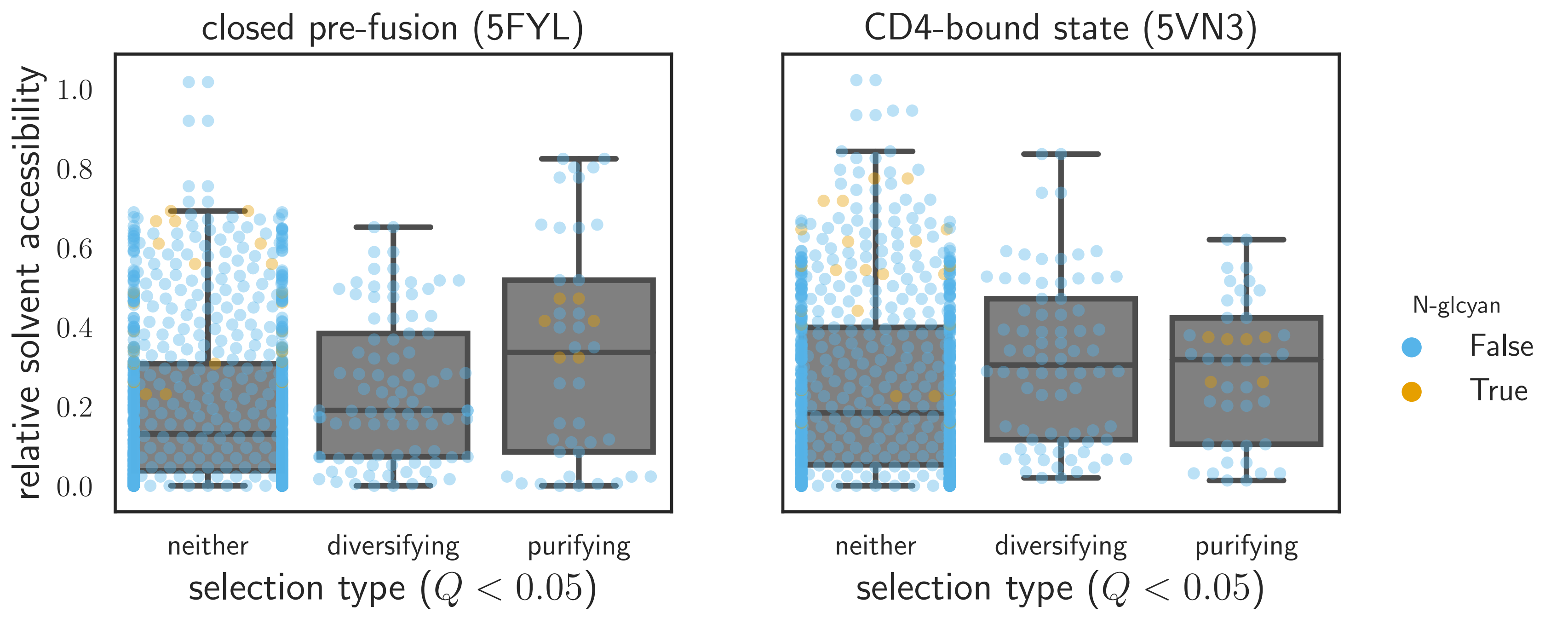

closed pre-fusion (5FYL)
CD4-bound state (5VN3)
relative solvent accessibility
1.0
0.8
0.6
0.4
0.2
0.0
neither
diversifying
purifying
selection type ($Q < 0.05$)
N-glcyan
False
True
