# Supplementary material for: Mapping mutational effects along the evolutionary landscape of HIV envelope
Source: eLife. 2018 Mar 28;7:e34420. doi: 10.7554/eLife.34420 (PMC5910023; doi:10.7554/eLife.34420)
Supplement: Supplementary file 1. — Specifically, this file contains a Jupyter notebook that performs the analysis, all required input data, and all reasonably sized output files. The Jupyter notebook downloads the deep sequencing data, processes it with the dms_tools2 software (Bloom, 2015, https://jbloomlab.github.io/dms_tools2/), and also performs a variety of downstream analyses that generate most of the figures for this paper. [file elife-34420-supp1.zip › analysis_code/results/prefs/allprefscorr.pdf]

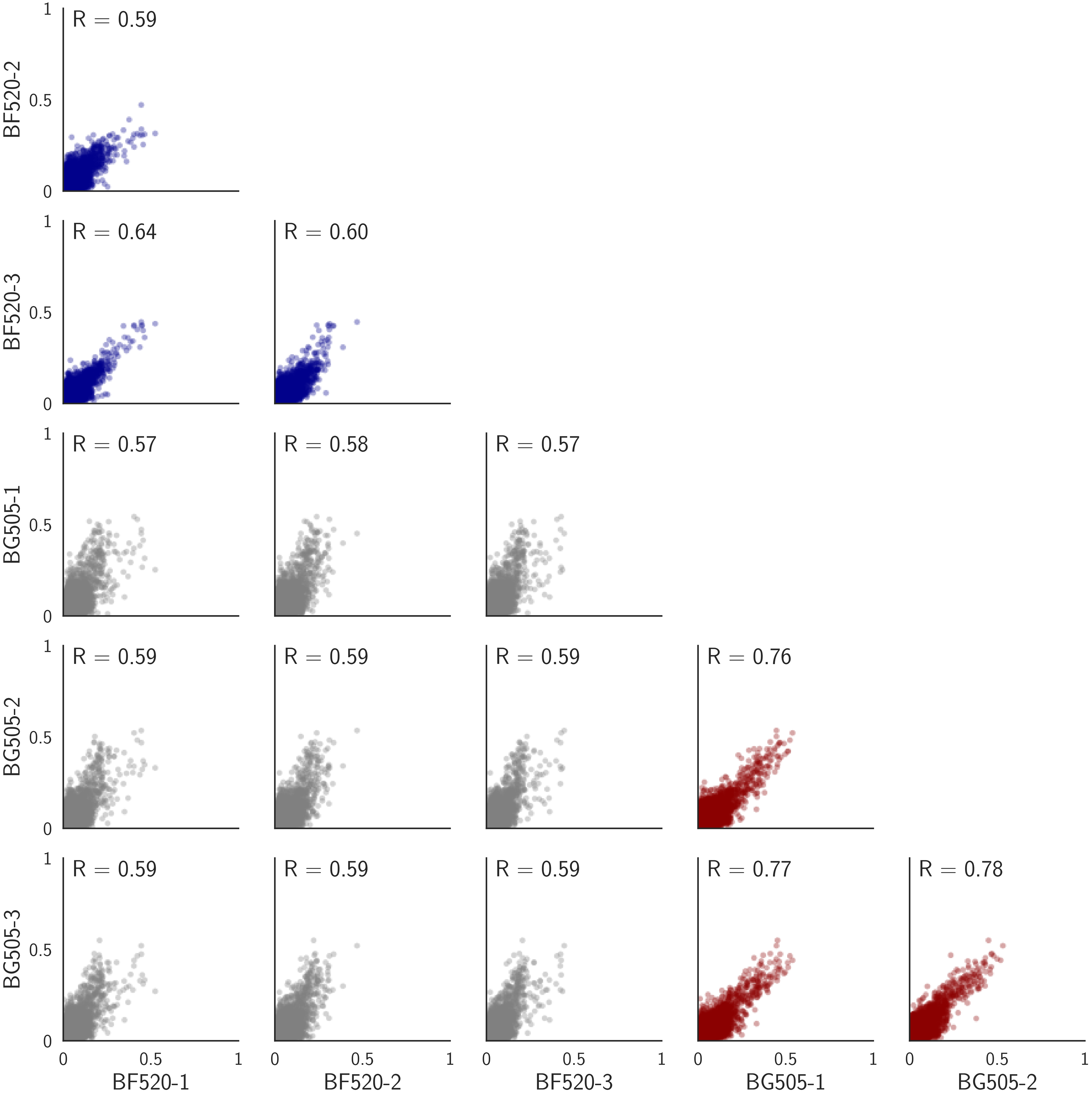

R = 0.59
R = 0.64
R = 0.60
R = 0.57
R = 0.58
R = 0.57
R = 0.59
R = 0.59
R = 0.59
R = 0.76
R = 0.59
R = 0.59
R = 0.59
R = 0.77
R = 0.78
BF520-2
BF520-3
BG505-1
BG505-2
BG505-3
BF520-1
BF520-2
BF520-3
BG505-1
BG505-2
0
0.5
1
