# Supplementary material for: Mapping mutational effects along the evolutionary landscape of HIV envelope
Source: eLife. 2018 Mar 28;7:e34420. doi: 10.7554/eLife.34420 (PMC5910023; doi:10.7554/eLife.34420)
Supplement: Supplementary file 1. — Specifically, this file contains a Jupyter notebook that performs the analysis, all required input data, and all reasonably sized output files. The Jupyter notebook downloads the deep sequencing data, processes it with the dms_tools2 software (Bloom, 2015, https://jbloomlab.github.io/dms_tools2/), and also performs a variety of downstream analyses that generate most of the figures for this paper. [file elife-34420-supp1.zip › analysis_code/results/prefs/diversifying_selsites.pdf]

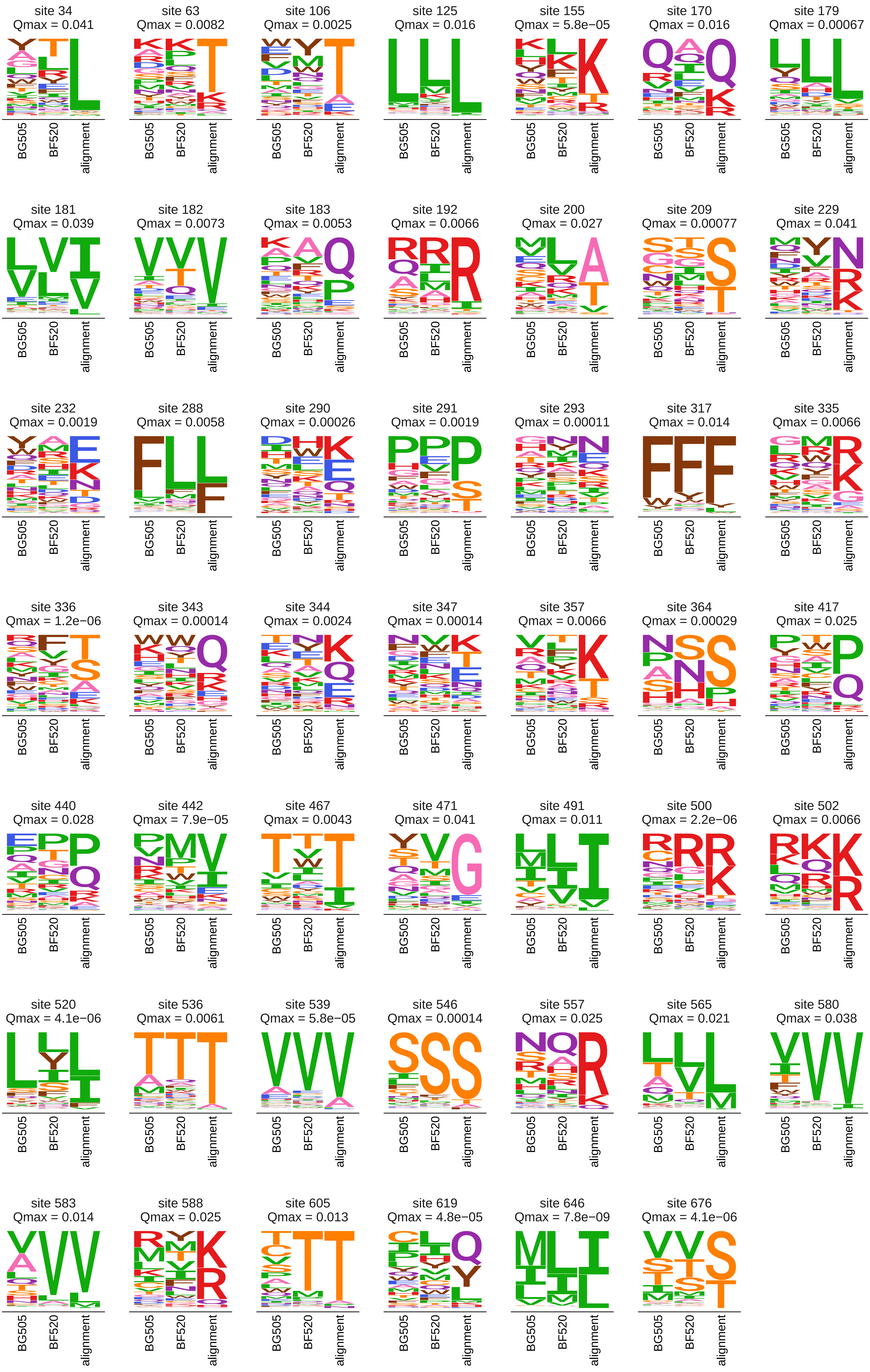
site 34
Qmax = 0.041
site 63
Qmax = 0.0082
site 106
Qmax = 0.0025
site 125
Qmax = 0.016
site 155
Qmax = 5.8e−05
site 170
Qmax = 0.016
site 179
Qmax = 0.00067
site 181
Qmax = 0.039
site 182
Qmax = 0.0073
site 183
Qmax = 0.0053
site 192
Qmax = 0.0066
site 200
Qmax = 0.027
site 209
Qmax = 0.00077
site 229
Qmax = 0.041
site 232
Qmax = 0.0019
site 288
Qmax = 0.0058
site 290
Qmax = 0.00026
site 291
Qmax = 0.0019
site 293
Qmax = 0.00011
site 317
Qmax = 0.014
site 335
Qmax = 0.0066
site 336
Qmax = 1.2e−06
site 343
Qmax = 0.00014
site 344
Qmax = 0.0024
site 347
Qmax = 0.00014
site 357
Qmax = 0.0066
site 364
Qmax = 0.00029
site 417
Qmax = 0.025
site 440
Qmax = 0.028
site 442
Qmax = 7.9e−05
site 467
Qmax = 0.0043
site 471
Qmax = 0.041
site 491
Qmax = 0.011
site 500
Qmax = 2.2e−06
site 502
Qmax = 0.0066
site 520
Qmax = 4.1e−06
site 536
Qmax = 0.0061
site 539
Qmax = 5.8e−05
site 546
Qmax = 0.00014
site 557
Qmax = 0.025
site 565
Qmax = 0.021
site 580
Qmax = 0.038
site 583
Qmax = 0.014
site 588
Qmax = 0.025
site 605
Qmax = 0.013
site 619
Qmax = 4.8e−05
site 646
Qmax = 7.8e−09
site 676
Qmax = 4.1e−06
BG505
BF520
alignment
