# Supplementary material for: Mapping mutational effects along the evolutionary landscape of HIV envelope
Source: eLife. 2018 Mar 28;7:e34420. doi: 10.7554/eLife.34420 (PMC5910023; doi:10.7554/eLife.34420)
Supplement: Supplementary file 1. — Specifically, this file contains a Jupyter notebook that performs the analysis, all required input data, and all reasonably sized output files. The Jupyter notebook downloads the deep sequencing data, processes it with the dms_tools2 software (Bloom, 2015, https://jbloomlab.github.io/dms_tools2/), and also performs a variety of downstream analyses that generate most of the figures for this paper. [file elife-34420-supp1.zip › analysis_code/results/prefs/glycan_prefs.pdf]

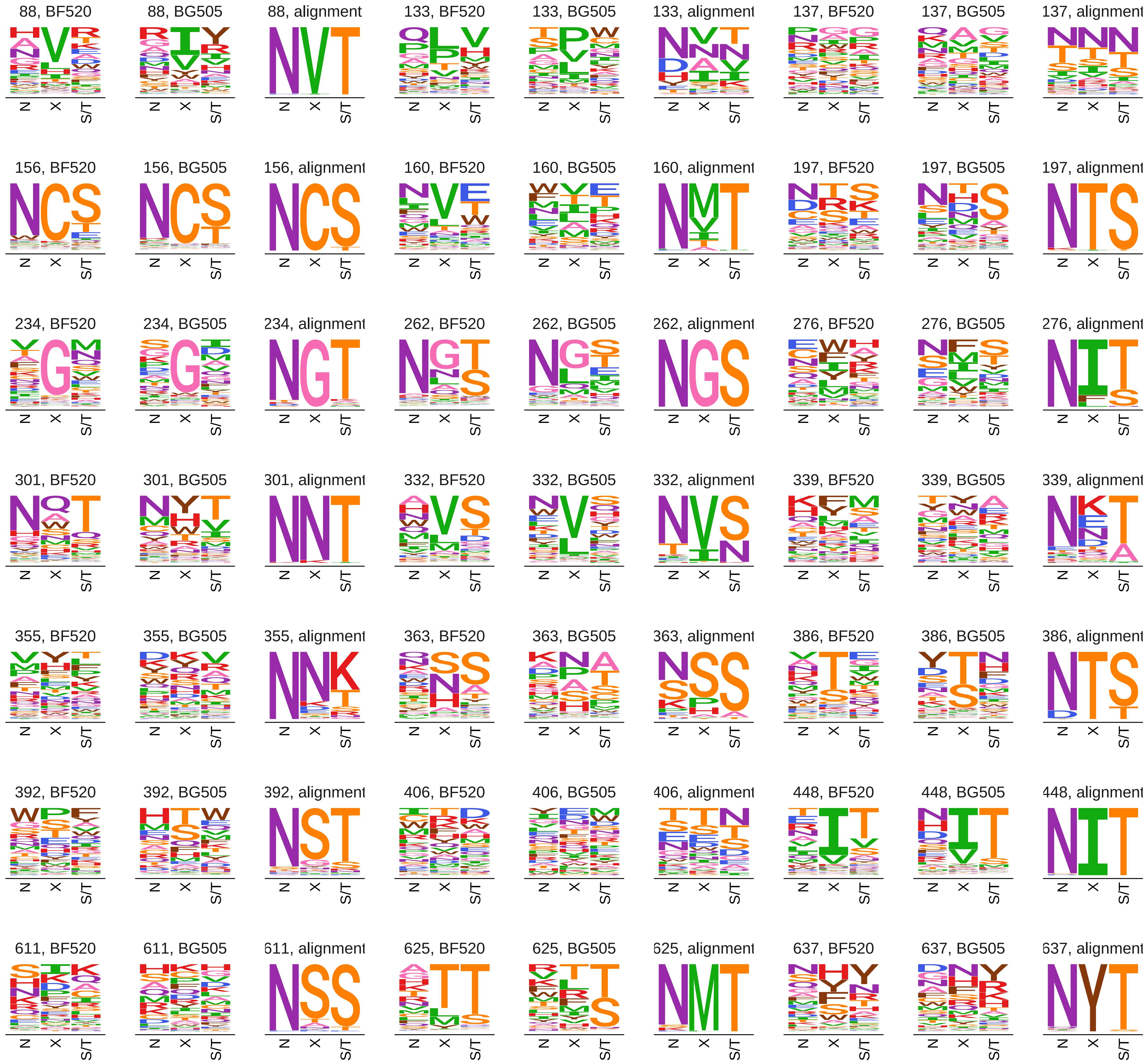
88, BF520
88, BG505
88, alignment
133, BF520
133, BG505
133, alignment
137, BF520
137, BG505
137, alignment
156, BF520
156, BG505
156, alignment
160, BF520
160, BG505
160, alignment
197, BF520
197, BG505
197, alignment
234, BF520
234, BG505
234, alignment
262, BF520
262, BG505
262, alignment
276, BF520
276, BG505
276, alignment
301, BF520
301, BG505
301, alignment
332, BF520
332, BG505
332, alignment
339, BF520
339, BG505
339, alignment
355, BF520
355, BG505
355, alignment
363, BF520
363, BG505
363, alignment
386, BF520
386, BG505
386, alignment
392, BF520
392, BG505
392, alignment
406, BF520
406, BG505
406, alignment
448, BF520
448, BG505
448, alignment
611, BF520
611, BG505
611, alignment
625, BF520
625, BG505
625, alignment
637, BF520
637, BG505
637, alignment
N
X
S/T
