# Supplementary material for: Mapping mutational effects along the evolutionary landscape of HIV envelope
Source: eLife. 2018 Mar 28;7:e34420. doi: 10.7554/eLife.34420 (PMC5910023; doi:10.7554/eLife.34420)
Supplement: Supplementary file 1. — Specifically, this file contains a Jupyter notebook that performs the analysis, all required input data, and all reasonably sized output files. The Jupyter notebook downloads the deep sequencing data, processes it with the dms_tools2 software (Bloom, 2015, https://jbloomlab.github.io/dms_tools2/), and also performs a variety of downstream analyses that generate most of the figures for this paper. [file elife-34420-supp1.zip › analysis_code/results/prefs/purifying_selsites.pdf]

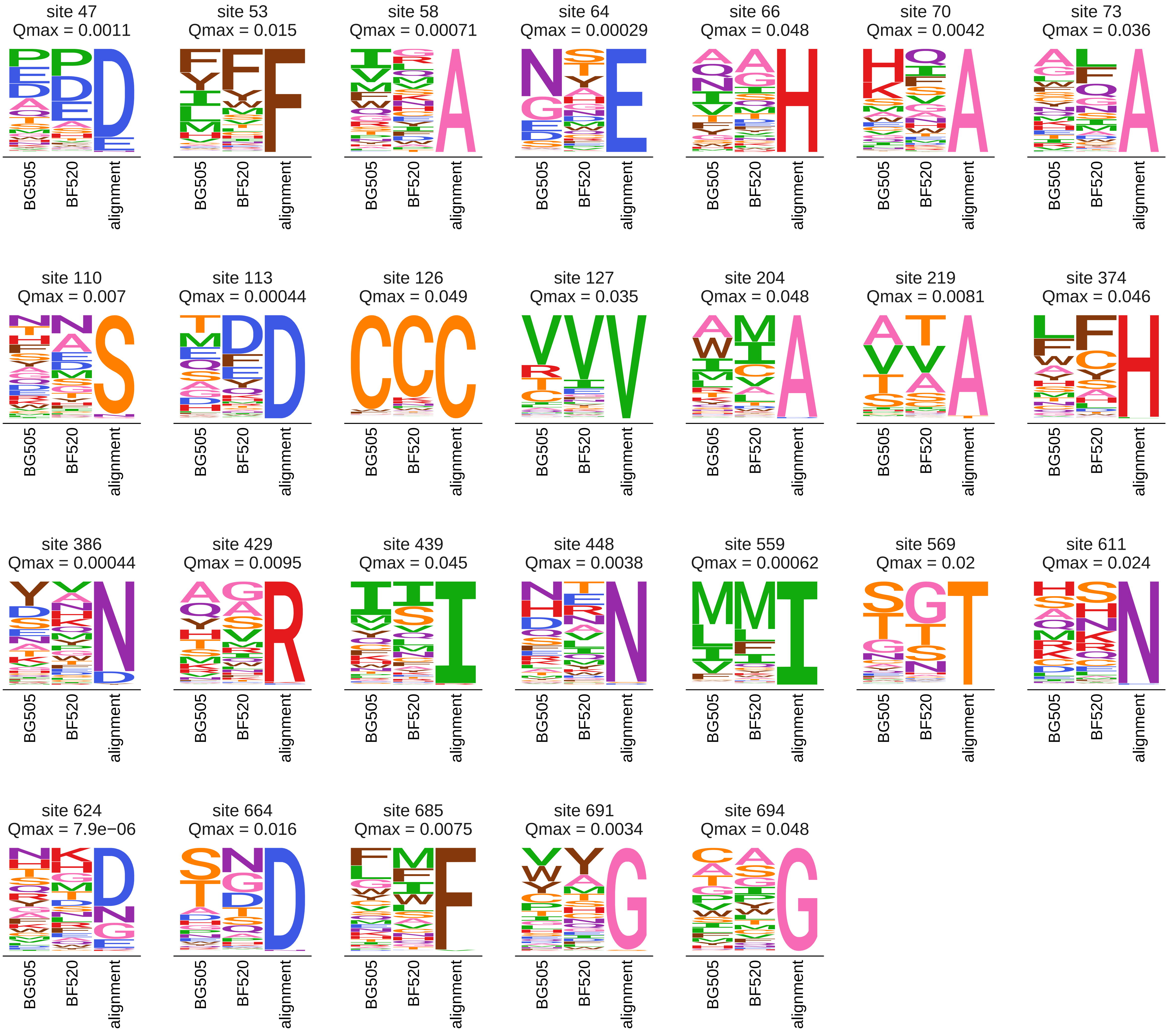
site 47
Qmax = 0.0011
site 53
Qmax = 0.015
site 58
Qmax = 0.00071
site 64
Qmax = 0.00029
site 66
Qmax = 0.048
site 70
Qmax = 0.0042
site 73
Qmax = 0.036
site 110
Qmax = 0.007
site 113
Qmax = 0.00044
site 126
Qmax = 0.049
site 127
Qmax = 0.035
site 204
Qmax = 0.048
site 219
Qmax = 0.0081
site 374
Qmax = 0.046
site 386
Qmax = 0.00044
site 429
Qmax = 0.0095
site 439
Qmax = 0.045
site 448
Qmax = 0.0038
site 559
Qmax = 0.00062
site 569
Qmax = 0.02
site 611
Qmax = 0.024
site 624
Qmax = 7.9e−06
site 664
Qmax = 0.016
site 685
Qmax = 0.0075
site 691
Qmax = 0.0034
site 694
Qmax = 0.048
BG505
BF520
alignment
