# Supplementary material for: Mapping mutational effects along the evolutionary landscape of HIV envelope
Source: eLife. 2018 Mar 28;7:e34420. doi: 10.7554/eLife.34420 (PMC5910023; doi:10.7554/eLife.34420)
Supplement: Supplementary file 1. — Specifically, this file contains a Jupyter notebook that performs the analysis, all required input data, and all reasonably sized output files. The Jupyter notebook downloads the deep sequencing data, processes it with the dms_tools2 software (Bloom, 2015, https://jbloomlab.github.io/dms_tools2/), and also performs a variety of downstream analyses that generate most of the figures for this paper. [file elife-34420-supp1.zip › analysis_code/results/prefsdist/BG505-to-BF520_diffprefs.pdf]

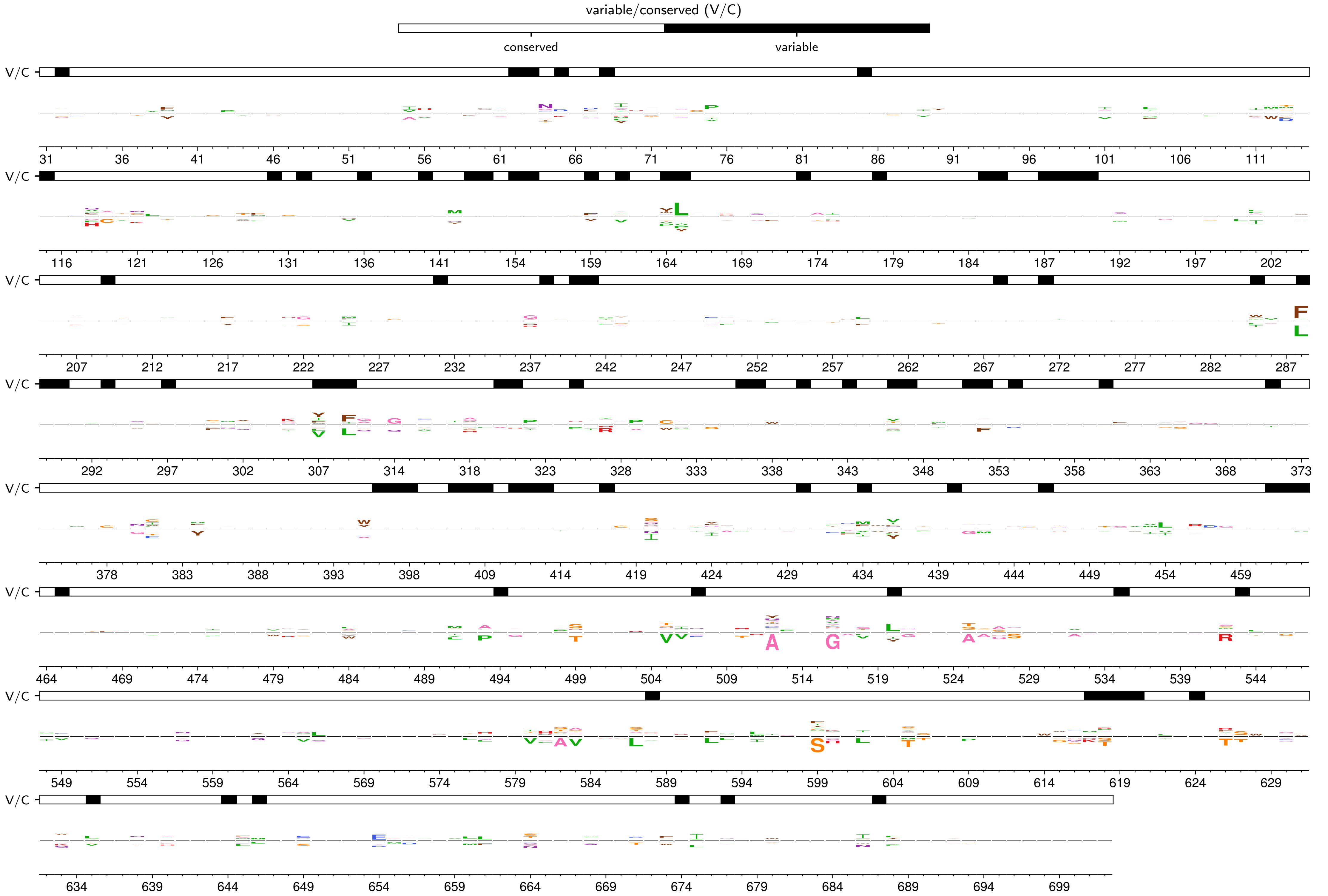
variable/conserved (V/C)
conserved
variable
V/C
31 36 41 46 51 56 61 66 71 76 81 86 91 96 101 106 111
116 121 126 131 136 141 154 159 164 169 174 179 184 187 192 197 202
207 212 217 222 227 232 237 242 247 252 257 262 267 272 277 282 287
292 297 302 307 314 318 323 328 333 338 343 348 353 358 363 368 373
378 383 388 393 398 409 414 419 424 429 434 439 444 449 454 459
464 469 474 479 484 489 494 499 504 509 514 519 524 529 534 539 544
549 554 559 564 569 574 579 584 589 594 599 604 609 614 619 624 629
634 639 644 649 654 659 664 669 674 679 684 689 694 699
