# Supplementary material for: Mapping mutational effects along the evolutionary landscape of HIV envelope
Source: eLife. 2018 Mar 28;7:e34420. doi: 10.7554/eLife.34420 (PMC5910023; doi:10.7554/eLife.34420)
Supplement: Supplementary file 1. — Specifically, this file contains a Jupyter notebook that performs the analysis, all required input data, and all reasonably sized output files. The Jupyter notebook downloads the deep sequencing data, processes it with the dms_tools2 software (Bloom, 2015, https://jbloomlab.github.io/dms_tools2/), and also performs a variety of downstream analyses that generate most of the figures for this paper. [file elife-34420-supp1.zip › analysis_code/results/prefsdist/conservation_vs_shifts.pdf]

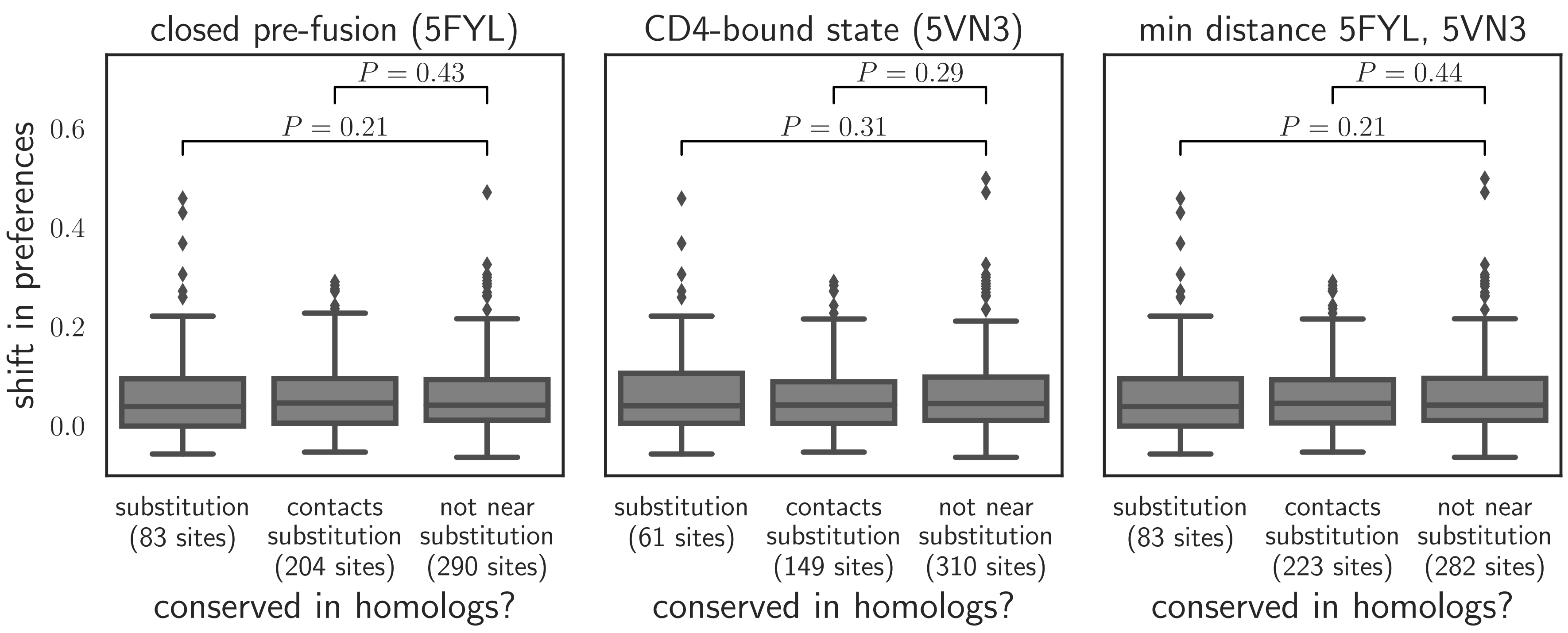
closed pre-fusion (5FYL)
CD4-bound state (5VN3)
min distance 5FYL, 5VN3
shift in preferences
0.0
0.2
0.4
0.6
P = 0.43
P = 0.21
P = 0.29
P = 0.31
P = 0.44
P = 0.21
substitution (83 sites)
contacts substitution (204 sites)
not near substitution (290 sites)
substitution (61 sites)
contacts substitution (149 sites)
not near substitution (310 sites)
substitution (83 sites)
contacts substitution (223 sites)
not near substitution (282 sites)
conserved in homologs?
conserved in homologs?
conserved in homologs?
