# Supplementary material for: Mapping mutational effects along the evolutionary landscape of HIV envelope
Source: eLife. 2018 Mar 28;7:e34420. doi: 10.7554/eLife.34420 (PMC5910023; doi:10.7554/eLife.34420)
Supplement: Supplementary file 1. — Specifically, this file contains a Jupyter notebook that performs the analysis, all required input data, and all reasonably sized output files. The Jupyter notebook downloads the deep sequencing data, processes it with the dms_tools2 software (Bloom, 2015, https://jbloomlab.github.io/dms_tools2/), and also performs a variety of downstream analyses that generate most of the figures for this paper. [file elife-34420-supp1.zip › analysis_code/results/prefsdist/conservation_vs_shifts_2.pdf]

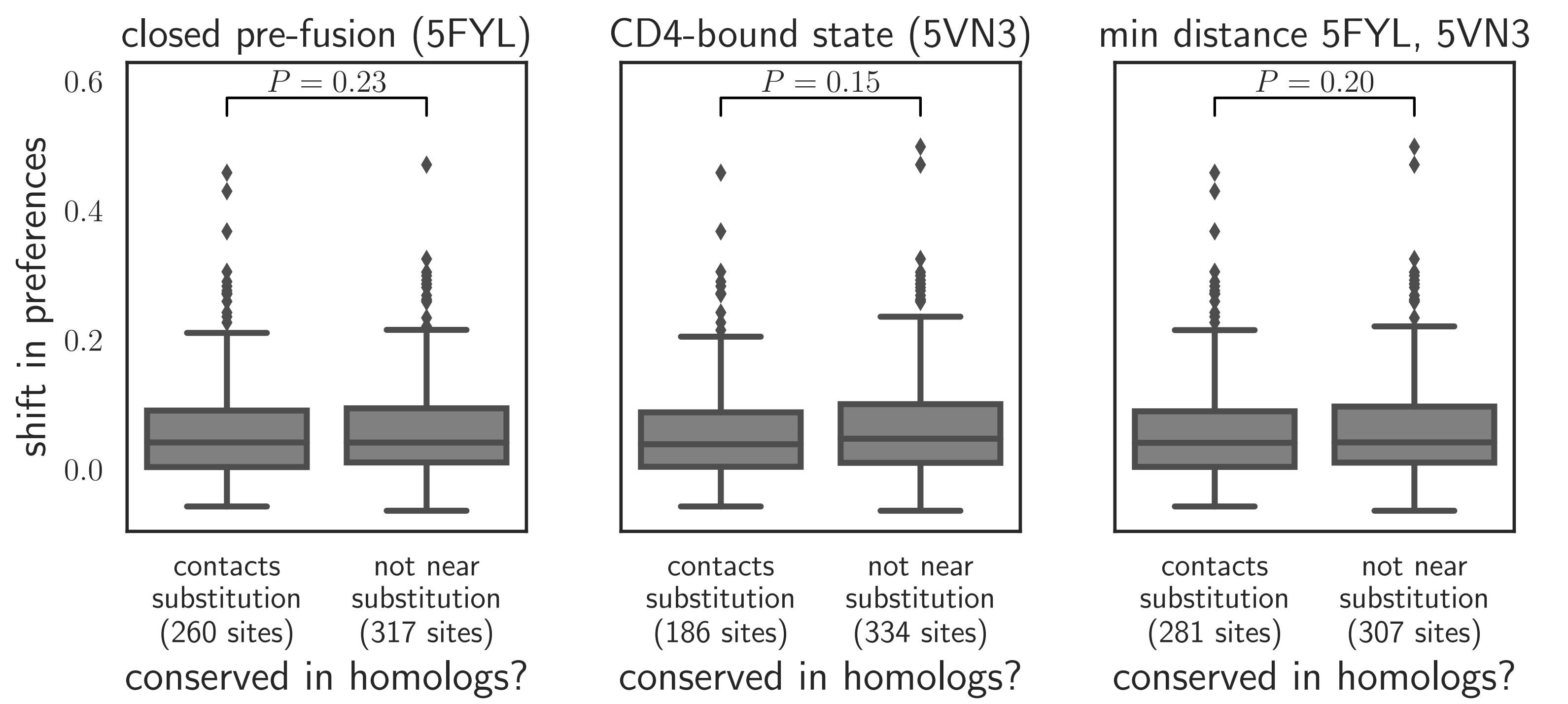
closed pre-fusion (5FYL)
CD4-bound state (5VN3)
min distance 5FYL, 5VN3
$P = 0.23$
$P = 0.15$
$P = 0.20$
shift in preferences
0.6
0.4
0.2
0.0
contacts substitution (260 sites)
not near substitution (317 sites)
contacts substitution (186 sites)
not near substitution (334 sites)
contacts substitution (281 sites)
not near substitution (307 sites)
conserved in homologs?
conserved in homologs?
conserved in homologs?
