# Supplementary material for: Mapping mutational effects along the evolutionary landscape of HIV envelope
Source: eLife. 2018 Mar 28;7:e34420. doi: 10.7554/eLife.34420 (PMC5910023; doi:10.7554/eLife.34420)
Supplement: Supplementary file 1. — Specifically, this file contains a Jupyter notebook that performs the analysis, all required input data, and all reasonably sized output files. The Jupyter notebook downloads the deep sequencing data, processes it with the dms_tools2 software (Bloom, 2015, https://jbloomlab.github.io/dms_tools2/), and also performs a variety of downstream analyses that generate most of the figures for this paper. [file elife-34420-supp1.zip › analysis_code/results/prefsdist/entrenchment.pdf]

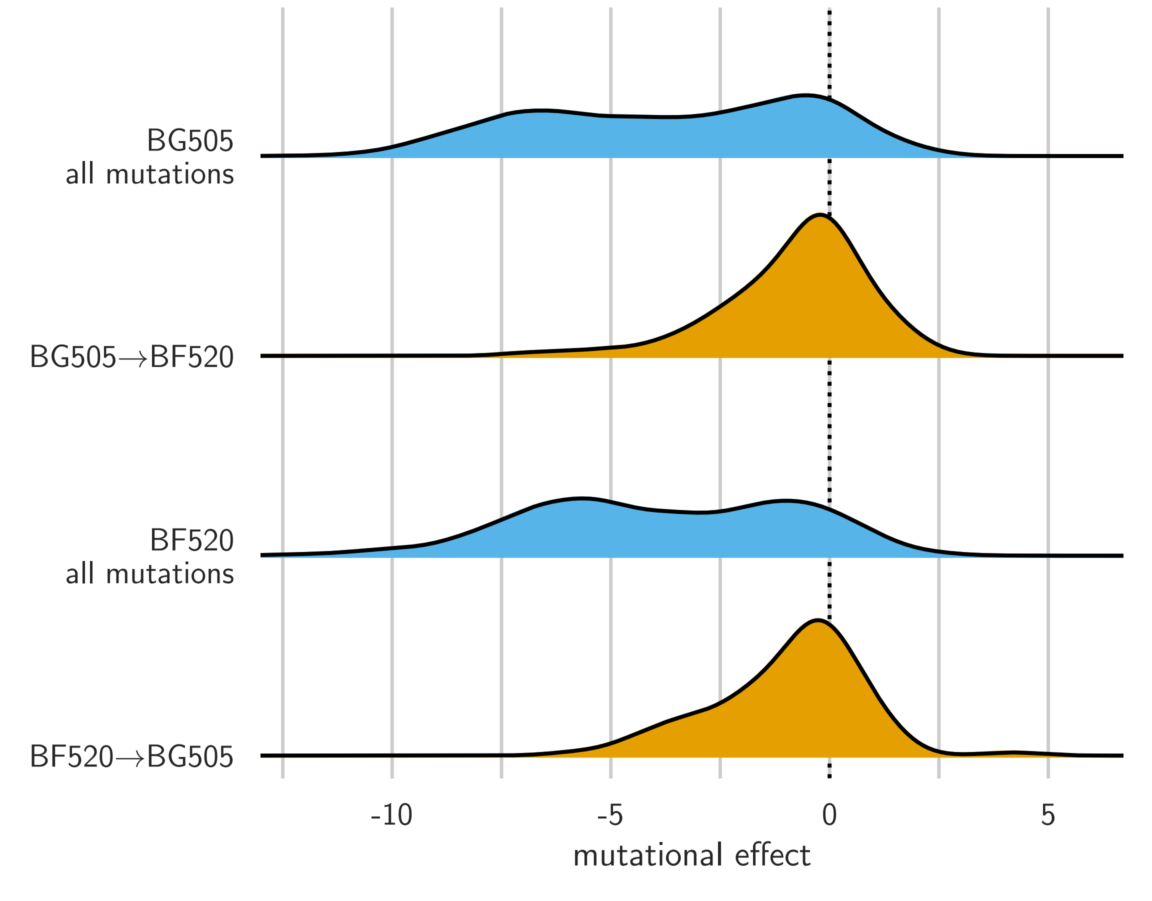

BG505
all mutations
BG505→BF520
BF520
all mutations
BF520→BG505
-10
-5
0
5
mutational effect
