# Supplementary material for: Mapping mutational effects along the evolutionary landscape of HIV envelope
Source: eLife. 2018 Mar 28;7:e34420. doi: 10.7554/eLife.34420 (PMC5910023; doi:10.7554/eLife.34420)
Supplement: Supplementary file 1. — Specifically, this file contains a Jupyter notebook that performs the analysis, all required input data, and all reasonably sized output files. The Jupyter notebook downloads the deep sequencing data, processes it with the dms_tools2 software (Bloom, 2015, https://jbloomlab.github.io/dms_tools2/), and also performs a variety of downstream analyses that generate most of the figures for this paper. [file elife-34420-supp1.zip › analysis_code/results/prefsdist/shifted_sites.pdf]

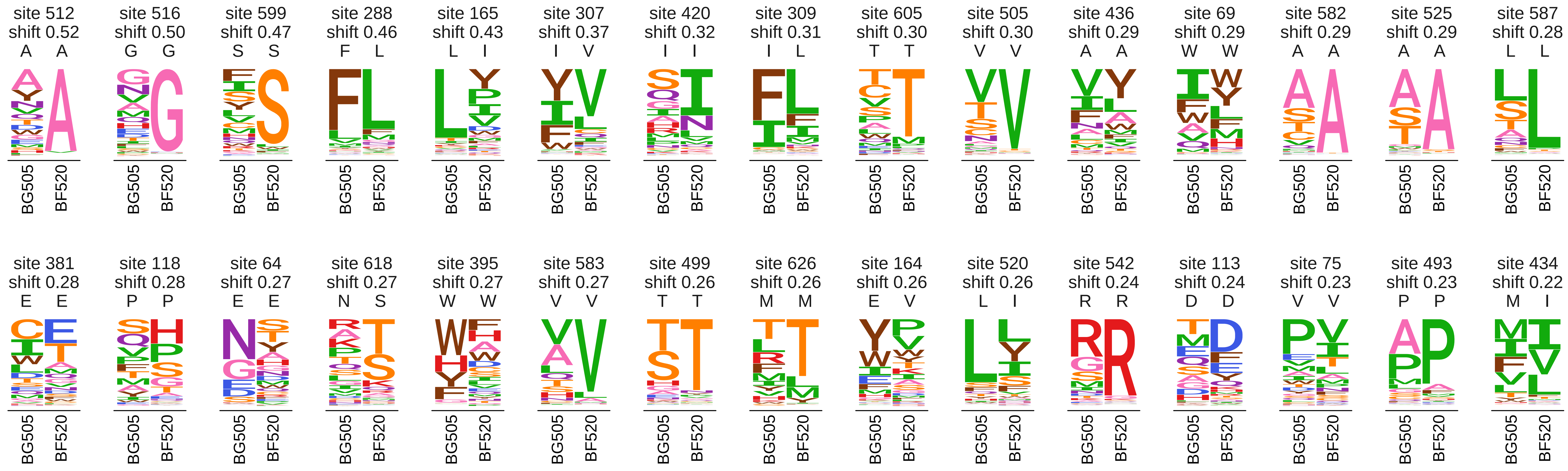
site 512
shift 0.52
A A
site 516
shift 0.50
G G
site 599
shift 0.47
S S
site 288
shift 0.46
F L
site 165
shift 0.43
L I
site 307
shift 0.37
I V
site 420
shift 0.32
I I
site 309
shift 0.31
I L
site 605
shift 0.30
T T
site 505
shift 0.30
V V
site 436
shift 0.29
A A
site 69
shift 0.29
W W
site 582
shift 0.29
A A
site 525
shift 0.29
A A
site 587
shift 0.28
L L
site 381
shift 0.28
E E
site 118
shift 0.28
P P
site 64
shift 0.27
E E
site 618
shift 0.27
N S
site 395
shift 0.27
W W
site 583
shift 0.27
V V
site 499
shift 0.26
T T
site 626
shift 0.26
M M
site 164
shift 0.26
E V
site 520
shift 0.26
L I
site 542
shift 0.24
R R
site 113
shift 0.24
D D
site 75
shift 0.23
V V
site 493
shift 0.23
P P
site 434
shift 0.22
M I
BG505
BF520
